# CalScope: methodology and lessons learned for conducting a remote statewide SARS-CoV-2 seroprevalence study in California using an at-home dried blood spot collection kit and online survey

**DOI:** 10.1186/s12874-024-02245-y

**Published:** 2024-05-27

**Authors:** Esther Lim, Megha L. Mehrotra, Katherine Lamba, Amanda Kamali, Kristina W. Lai, Erika Meza, Stephanie Bertsch-Merbach, Irvin Szeto, Catherine Ley, Andrew B. Martin, Julie Parsonnet, Peter Robinson, David Gebhart, Noemi Fonseca, Cheng-ting Tsai, David Seftel, Allyx Nicolici, David Melton, Seema Jain

**Affiliations:** 1 California Department of Public Health, Epidemiology, Surveillance, and Modeling Section, COVID-19 Response, Richmond, CA USA; 2 Department of Medicine, Stanford University School of Medicine, Stanford, CA USA; 3 Research IT, School of Medicine, Stanford University, Palo Alto, CA USA; 4 Enable Biosciences, South San Francisco, CA USA

**Keywords:** Population-based surveillance, SARS-CoV-2, Health Department, Remote, At home testing

## Abstract

**Background:**

To describe the methodology for conducting the CalScope study, a remote, population-based survey launched by the California Department of Public Health (CDPH) to estimate SARS-CoV-2 seroprevalence and understand COVID-19 disease burden in California.

**Methods:**

Between April 2021 and August 2022, 666,857 randomly selected households were invited by mail to complete an online survey and at-home test kit for up to one adult and one child. A gift card was given for each completed survey and test kit. Multiple customized REDCap databases were used to create a data system which provided task automation and scalable data management through API integrations. Support infrastructure was developed to manage follow-up for participant questions and a communications plan was used for outreach through local partners.

**Results:**

Across 3 waves, 32,671 out of 666,857 (4.9%) households registered, 6.3% by phone using an interactive voice response (IVR) system and 95.7% in English. Overall, 25,488 (78.0%) households completed surveys, while 23,396 (71.6%) households returned blood samples for testing. Support requests (*n* = 5,807) received through the web-based form (36.3%), by email (34.1%), and voicemail (29.7%) were mostly concerned with the test kit (31.6%), test result (26.8%), and gift card (21.3%).

**Conclusions:**

Ensuring a well-integrated and scalable data system, responsive support infrastructure for participant follow-up, and appropriate academic and local health department partnerships for study management and communication allowed for successful rollout of a large population-based survey. Remote data collection utilizing online surveys and at-home test kits can complement routine surveillance data for a state health department.

**Supplementary Information:**

The online version contains supplementary material available at 10.1186/s12874-024-02245-y.

## Background

In April 2021, the California Department of Public Health (CDPH) partnered with Stanford University to launch CalScope for estimating the proportion of Californians with evidence of SARS-CoV-2 antibodies from prior infection or vaccination [1]. CalScope was adapted from the CA-FACTS [2] study and designed to address three main challenges as part of CDPH’s enhanced surveillance efforts to better monitor the COVID-19 pandemic over time and inform public health action: the high proportion of cases not being reported to the state surveillance database due to limited testing capacity and mild or asymptomatic cases, a population of nearly 40 million residents spread throughout a large and diverse state, and stay-at-home orders which limited in-person study administration.

The objective of this paper is to describe the procedures for conducting CalScope, including the study design, data and support systems, and communication plan.

## Methods

CalScope used random address-based sampling over three collection periods to invite households from seven counties in California, which were chosen to facilitate efficient sampling of the entire state, focus community-based outreach efforts to smaller geographic areas, and allow for region-specific estimates of seroprevalence. Up to one adult (18 + years old) and/or one child in each randomly selected household were invited to participate by completing an anonymous online survey and providing a self-administered, at-home SARS-CoV-2 antibody blood sample via a test kit with dried blood spot (DBS) card. Further details on the sampling strategy and methods have been described previously [1].

### Study process

**Fig. 1:**
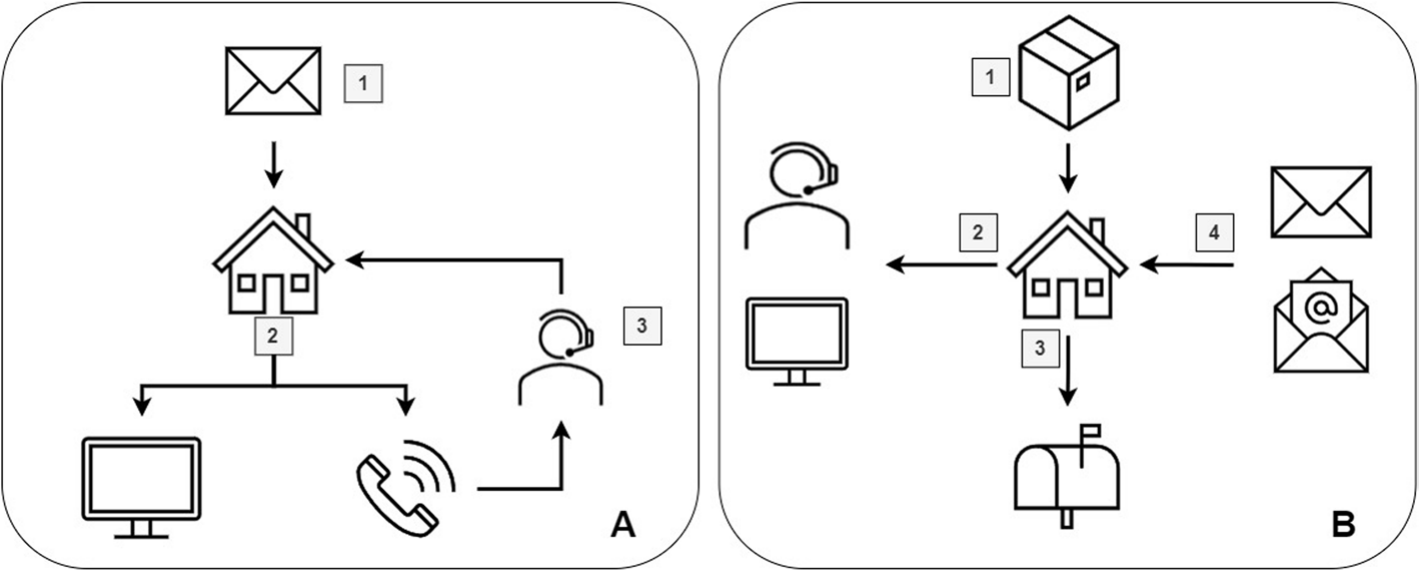
Participation schema. (**A**) Registration and Survey only: 1. Household receives mailed letter or postcard invitation. 2. Adult registers online or by phone. 3. Study team contacts survey-only households without internet to complete survey over the phone. (**B**) Survey and Test Kit: 1. Household receives test kit by mail. 2. Adult activates the test kit online or calls study team for assistance. 3. Household mails back completed test kit. 4. Household receives test results by mail. Gift cards are delivered by mail or email

Each selected household received a mailed invitation letter and follow-up postcard with a unique, address-linked, 8-digit code with instructions on how to register for the study online or over the phone (Fig. 1A). For participants without internet access, an interactive voice response (IVR) system (automated telephone system using pre-recorded messages) accepted registrations over the phone.

The registration form required an adult (aged 18+) from each household to enter the number of eligible adults and children in residence. Based on this enumeration, households had three options for study participation: (1) complete the adult survey-only option without ordering any test kits, (2) complete one adult survey with test kit and a child survey-only option (or vice versa), or (3) complete surveys with test kits for both an adult and child. Full participation in the study was defined as completion of both a survey and test kit per participant. Households without children could order only one adult survey with test kit or complete the adult survey-only option. Households with multiple adults and/or children were instructed to randomize participation by enrolling the member with the next upcoming birthday.

Registered households were mailed at-home SARS-CoV-2 antibody test kit(s) with instructions for completing the online survey, collecting a finger-prick DBS sample, and returning completed test kits in pre-addressed envelopes within two weeks. Upon return of test kits, DBS cards with adequate collections of blood samples were tested for the presence or absence of antibodies against the spike (S1) and nucleocapsid protein of SARS-CoV-2 [3]. The S1 results were mailed back to participants with no personally identifiable information, differentiating results by sample type (adult or child), collection date, and result date only. The letter also included information on how to interpret the results. Two $20 gift cards were disbursed to each participant, once at the completion of the online survey and again after the return of a completed test kit with DBS samples.

### Study surveys

All data collection materials were administered online using REDCap [4, 5]. Respondents completed forms for language selection, registration, adult or child surveys, and gift card preference. The language selection form supported English, Spanish, Filipino/Tagalog, and simplified Chinese, the four most common languages in the seven selected counties [6]. Participants who did not have internet access or had difficulty completing the online survey were asked to request help through IVR so that they could complete the survey with a study staff member over the phone.

The registration form included questions on number of adults and children in the household, choice of survey and/or at-home test kit orders, and optional contact information for study updates and reminders. The adult survey included questions on demographics, income, occupation, medical history, COVID-19 vaccination history, and risk factors for COVID-19 disease at both the household and individual level. The child survey included questions on individual demographics, medical history, COVID-19 vaccination history, and risk factors for COVID-19 disease. A gift card form at the end of each survey asked participants for their preferred method of delivery (mail or email).

### At-Home SARS-CoV-2 antibody test kits

Antibody test kits were mailed to registered households with a unique 6-character alphanumeric code for activation, which allowed participants to access the online survey and instructional video for blood collection (Fig. 1B). Each kit box included: (1) a 1-page letter outlining the steps for accessing the online survey and collecting the blood sample; (2) an instructional booklet on how to collect a DBS sample; (3) materials for blood collection (i.e. lancet, gauze, alcohol wipes, DBS card); and (4) a pre-paid United States Postal Service (USPS) mailer addressed to the testing laboratory (Enable Biosciences, South San Francisco, CA). After receipt at the laboratory, collected blood samples were tested for SARS-CoV-2 antibodies.

### Data system

**Fig. 2:**
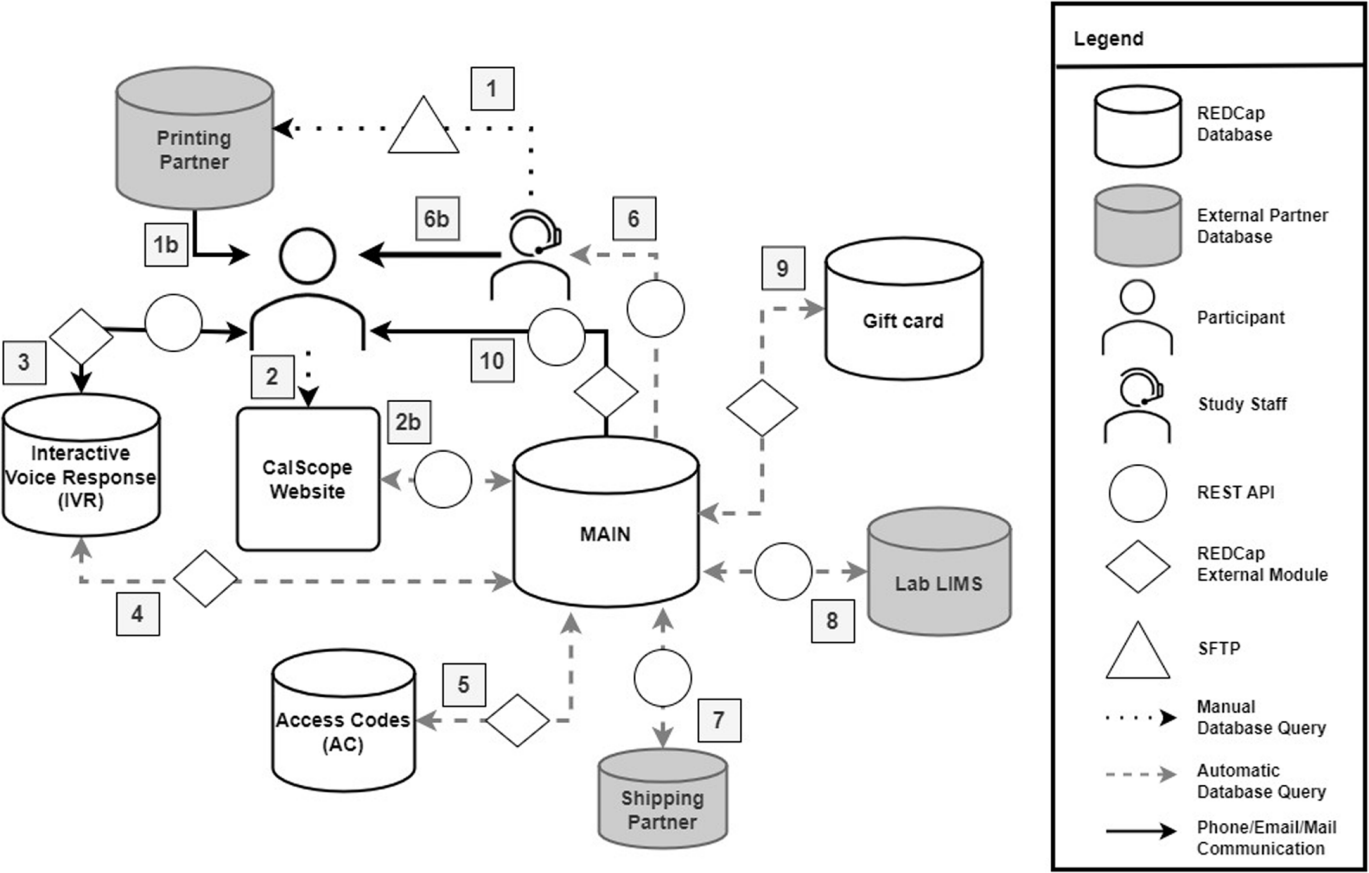
Data flow diagram for study information between various databases. The *Main* REDCap database was the primary repository communicating via REST API, updating survey, kit shipment, and laboratory results on a daily schedule. Numbered pathways show the connections involved with data management: (1) The sampling frame linked with the unique access codes (AC) were saved to *AC* and transferred to the printing partner through a web secure file transfer protocol (sFTP) client. (1b) Address-linked ACs were mailed out to households. (2) Adult participant entered in access and ZIP code combination into website, or (3) by phone, facilitated via Twilio’s API. (4) Scheduled task checked for new IVR registrations against existing records in *Main.* (5) *Main* queried *AC* to look up and verify entered combinations. If valid and unique, new record was created in *Main* to open the registration form and for *IVR*, the order was copied over to a new *Main* record. (6-6b) Survey-only registrations by IVR were pulled to generate a follow-up list. (7) Information on orders and shipments (USPS tracking number, activation code, and DBS codes) updated through API between *Main* and *Shipping Partner*. USPS API tracked shipment deliveries and returns. (8) Kit shipments (DBS codes, tracking numbers) received from the *Shipping Partner* and *Main* record ID were relayed to the laboratory by API. (2-2b) Activation and zip code combinations on test kits were entered into the website, which located the *Main* record by API and displayed personalized survey link(s) for the test kit. DBS completion steps were viewable at completion of at least 1 survey. (8) Processing and result information for returned test kits were pulled from the *Lab* into *Main* using API. (9) When compensation criteria were met in *Main*, an external module (EM) assigned the next available *Gift Card* link to the *Main* record. (10) *Main* sent email/text for reminders, confirmations, and compensation based on programmed logic

Four REDCap databases were used to manage study data with external modules [7–10] and communicated with external databases via Application Programming Interface (API) to update information on test kit shipment, return, and lab results on a daily schedule (Fig. 2). Each address was linked with the unique access code that was printed on the invitation. Printed invitations were delivered by USPS, and households could register online using the access and ZIP codes on the letter or postcard. If the entered combination was valid and unused, a new registration form could be filled out, and the address associated with the access code was displayed to make sure a registration was being completed by the intended household. Alternatively, registrations could be completed by phone utilizing the IVR system to collect the same information as the online form, but the address validity check was performed after submission before an order was processed. Confirmation emails and text messages were sent out if contact information was provided during registration, allowing participants to confirm that the correct order information was received as well.

Once a registration form was approved, the appropriate order information (including mailing address and test kit type) was sent to the shipping partner (ALOM, Fremont, CA). Upon fulfillment, information including the USPS tracking number, activation code, and DBS card ID numbers were saved to the household record and sent to the laboratory in preparation for accessioning once test kits were returned by participants.

When participants received their test kits, they were instructed to enter the printed activation and ZIP codes into the study website. When the correct combination was entered, the adult and/or child survey link(s) for the household was displayed along with instructions on collection and return of the blood sample. After the laboratory tested the samples, results were returned to participants by mail. Gift cards were then sent to participants using their specified delivery method.

Data from each REDCap database were exported daily and cleaned via an automated program using SAS version 9.4 (SAS Institute Inc., Cary, NC). Study staff used these datasets to create reports, track enrollment, monitor data quality, flag errors, and ensure that all data exchanges occurred correctly throughout the study period.

### Support requests and follow-up

Six to ten members of the CDPH California Connected contact tracing team provided follow-up support for participants over the phone and by email. Experienced with conducting contact tracing interviews, this support team was trained with a call center protocol and phone script. A CalScope study member was also available to provide technical support during follow-up. To follow up with participants who needed support in languages other than English, an interpreter service was utilized.

**Fig. 3:**
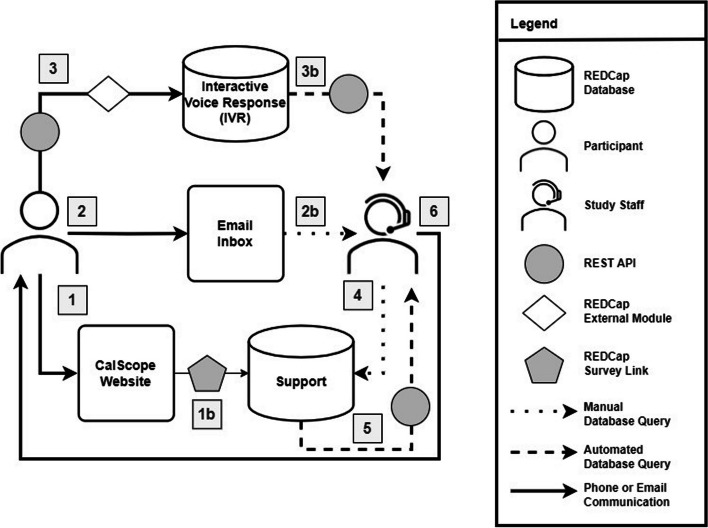
Data flow diagram for support and tracking follow-up activity. The Support REDCap database was the primary data repository for support requests where all participant communication received through the website, email, or voicemail was consolidated for follow-up documentation and tracking. Numbered pathways show the databases and connections involved with the management of support and follow-up activities: (1-1b) Participant submitted a web-based support form through the survey link labeled “Contact Us” on the study website to create a new request. (2-2b) Participant sent an email to the study inbox which was manually checked by study staff. (3) Participant left a voicemail by utilizing the IVR system enabled by Twilio’s API and an external module (EM). (3b) Automatic transcriptions generated by the EM were sent to study staff by email. (4) Study staff manually transferred incoming email and voicemail into *Support*, creating new support requests (5). Daily reports were generated for study staff to review and track open support requests. (8) Study staff followed-up with support requests by contacting participants through phone or email

Participants could submit support requests to the study team via: (1) a web-based “Contact Us” support form on the study website, (2) direct emails to the study inbox, or (3) a voicemail utilizing the IVR system (Fig. 3). Study staff consolidated requests and created daily reports to review and track open requests. Each request was assigned a number which was used for tracking as staff sent out emails, documented notes, and updated the resolution status for each follow-up event.

### Communication materials and community outreach

A web-based communications toolkit was developed to increase study awareness and community outreach, which contained a study FAQ, a participation infographic, outreach flyers, social media messages and graphics, templates for a press release, and radio scripts. Virtual presentations were regularly scheduled with local health departments in participating counties to provide study progress updates and review communication materials and outreach strategies for increasing study engagement and utilization of the toolkit. To promote general awareness, CDPH shared study information through social media messages and COVID-19 media updates. Local health departments were also encouraged to partner with community-based organizations to increase local visibility of the CalScope study.

## Results

### Registration

CalScope recruited participants through three separate samplings: 200,000 households in wave 1 (W1; 04/20/2021-06/16/2021), 200,000 households in wave 2 (W2; 10/15/2021-12/17/2022) and 266,857 in wave 3 (W3; 04/01/2022-07/29/2022) with test kit return deadlines on 08/01/2021, 02/11/2022, and 08/26/2022 respectively. The number of invited households overall was increased by 66,857 in W3 to adjust for decreasing participation rates between the first two waves.

Registration was highest in W1 with 5.6% of invited households responding to the invitation and decreased in subsequent waves (Table 1). The number of households that did not complete the registration form or opted out of both the kit and survey at the end of registration was 3.0% (344/11,286) in W1, 3.5% (318/9,019) in W2, and 4.2% (515/12,366) in W3. Across waves, 30.7% of registered households reported having at least 1 child in residence (W1 30.1%; W2 32.1%; W3 30.0%).

**Table 1:** Study participation across all three waves at the household and individual adult and child participant level

	Wave 1 (*n* = 200,000)		Wave 2 (*n* = 200,000)		Wave 3^a^ (*n* = 266,857)		Overall (*n* = 666,857)	
	n	(%)	n	(%)	n	(%)	n	(%)
Total registrations	**11,286**	**5.6**	**9019**	**4.5**	**12,366**	**4.6**	**32,671**	**4.9**
Survey and test kit orders^b^								
Survey-only	***881***	***7.8***	***384***	***4.3***	***728***	***5.9***	***1993***	***6.1***
* Adult*	*502*	*4.4*	*236*	*2.6*	*407*	*3.3*	*1145*	*3.5*
* Child*	*379*	*3.4*	*148*	*1.6*	*321*	*2.6*	*848*	*2.6*
Test kit	**10,444**	**92.5**	**8466**	**93.9**	**11,448**	**92.6**	**30,358**	**92.9**
* Adult*	*10,435*	*92.5*	*8465*	*93.8*	*11,442*	*92.5*	*30,342*	*92.9*
* Child*	*2656*	*23.5*	*2502*	*27.7*	*3043*	*24.6*	*8201*	*25.1*
Surveys completed								
Survey-only	**825**	**93.6**	**337**	**87.8**	**654**	**89.8**	**1816**	**91.1**
* Adult*	*468*	*93.2*	*204*	*86.4*	*370*	*90.9*	*1042*	*91.0*
* Child*	*357*	*94.2*	*133*	*89.9*	*284*	*88.5*	*774*	*91.3*
Test kit surveys^c^	**7863**	**75.3**	**7118**	**84.1**	**9318**	**81.4**	**24,299**	**80.0**
* Adult*	*7852*	*75.2*	*7115*	*84.1*	*9305*	*81.3*	*24,272*	*80.0*
* Child*	*1656*	*62.3*	*1903*	*76.1*	*2141*	*70.4*	*5700*	*69.5*
Test kits completed								
Test kits returned	**7846**	**75.1**	**6875**	**81.2**	**8866**	**77.4**	**23,587**	**77.7**
* Adult*	*7842*	*75.2*	*6874*	*81.2*	*8863*	*77.5*	*23,579*	*77.7*
* Child*	*1686*	*63.5*	*1857*	*74.2*	*2047*	*67.3*	*5590*	*68.2*
Adequate samples^d^	**7763**	**74.3**	**6834**	**80.7**	**8799**	**76.9**	**23,396**	**77.1**
* Adult*	*7753*	*74.3*	*6830*	*80.7*	*8793*	*76.8*	*23,376*	*77.0*
* Child*	*1438*	*54.1*	*1533*	*61.3*	*1632*	*53.6*	*4603*	*56.1*
Test kit returned with adequate sample and survey completed								
Full participation^e^	**7495**	**66.4**	**6792**	**75.3**	**8757**	**70.8**	**23,044**	**70.5**
* Adult*	*7484*	*66.3*	*6788*	*75.3*	*8750*	*70.8*	*23,022*	*70.5*
* Child*	*1383*	*12.3*	*1522*	*16.9*	*1621*	*13.1*	*4526*	*13.9*

^a^ The number of sampled households was increased by 66,857 for Wave 3 in anticipation of lowered interest in study participation and response rate based on Wave 2 enrollment totals

^b^ Households with children may have selected both the survey-only and test kit option for the household, resulting in a double counting of households between survey-only and test kit

^c^ The surveys used for the survey-only and test kit survey options were identical

^d^ Adequate samples were defined as DBS cards with sufficient volume of blood collected for testing

^e^ A participant was considered to have completed full participation when both an online survey was completed and a test kit with an adequate blood sample was returned for testing

### Overall study participation

Out of 666,857 households invited across three waves, 32,671 (4.9%) completed a registration form and a total of 31,496 (4.7%) completed an antibody test kit and/or online survey (Table 1). Among households that selected the survey-only option, the survey completion rate was 91.1% (1,816/1,993), with 91.0% (1,042/1,145) of adult and 91.3% (774/848) of child surveys completed. Among households that selected the survey and test-kit orders, the survey completion rate was 80.0% (24,299/30,358), with 80.0% (27,272/30,342) of adult and 69.5% (5,700/8,201) of child test kit surveys completed. Withdrawals from the study occurred only among households that had ordered test kits, with 0.1% (15/10,444) withdrawn in W1, 0.2% (14/8,466) in W2, and 0.4% (44/11,448) in W3.

Across three waves, 77.7% (23,587/30,358) of household test kits were returned. About 99.2% (23,396/23,587) of these households provided adequate samples on the DBS cards for testing (23,376 adult; 4,603 child). Sample rejections were more common among returned child kits (12.0%) compared to adult kits (0.7%). In total, 70.5% (23,043/32,671) of registered households (23,022 adult; 4,526 child) completed the survey and returned adequate blood samples for testing. In the end, 74.3% (24,270/32,671) of registered households completed partial or full participation in the study through the survey-only option (5.1%), the test kit-only option (92.5%), or a combination of the survey-only and test kit option (2.4%).

### Participation by IVR-registered households

Registrations completed through IVR accounted for 6.3% (2,065/32,671) of all registrations (W1 4.1%; W2 7.5%; W3 7.5%). Compared to 78.9% (24,136/30,606) of online-registered households that completed surveys, only 65.5% (1,352/2,065) of households that registered through IVR completed surveys. The percentage of test kits returned was also higher among online-registered households (78.5%), compared to IVR-registered households (66.2%). A smaller proportion of IVR households completed full participation in the study with 60.4% (1,247/2,065) returning adequate samples with a completed survey, compared to 71.2% (21,797/30,606) of online-registered households.

### Participation by language

Out of 32,671 total households registered, the majority completed registrations in English (95.7%), followed by Spanish (2.9%) and simplified Chinese (0.8%). Survey completion was higher in households that registered in English (78.9%) or simplified Chinese (78.3%), compared to households that registered in Filipino/Tagalog (66.7%) or Spanish (62.6%). In terms of full participation, 71.5% of households that registered in English (22,356), 66.7% simplified Chinese (172/258), 66.7% Filipino/Tagalog (6/9), and 53.9% Spanish (510/947) submitted adequate samples along with a complete survey.

### Support requests and follow-up

A total of 5,807 support requests were submitted to the study for follow-up: 2,476 (42.6%) in W1, 1,626 (28.0%) in W2, and 1,705 (29.4%) in W3. On average, support requests were followed-up by study staff within 1–2 business days and were resolved within 3 maximum follow-up attempts. In total, participants submitted 2,106 (36.3%) web-based forms, 1,978 (34.1%) emails, and 1,723 (29.7%) voicemail support requests. For method of contact, 3,890 (67.0%) requested follow-up by email, while 1,917 (33.0%) requested a follow-up phone call. The majority of requests (95.8%) were submitted in English, followed by Spanish (3.3%), simplified Chinese (0.9%), and only 1 request was submitted in Tagalog/Filipino.

Table 2 shows submitted participant questions by support type. Across waves, the highest number of support requests was for the test kit (1,836), which included participants requesting assistance with the test kit survey (30.6%), replacements for lost/missing kits (19.2%), and confirmation that the laboratory received the test kit (18.7%). Questions about test results (1,557) were the second highest reason for support requests, with most participants asking about their results or requesting re-delivery (94.5%).

**Table 2:** Study participant questions by support type

	Wave 1		Wave 2		Wave 3		Overall	
	(*n* = 2,476)		(*n* = 1,626)		(*n* = 1,705)		(*n* = 5,807)	
	**n**	**%**	**n**	**%**	**n**	**%**	**n**	**%**
General	**192**	**7.8**	**105**	**6.5**	**99**	**5.8**	**396**	**6.8**
Eligibility	57	31.8	15	14.3	24	24.2	96	24.2
Test Information	20	11.2	10	9.5	6	6.1	36	9.1
Feedback	15	7.3	6	5.7	10	10.1	31	7.8
Study procedures	78	33.0	51	48.6	51	51.5	180	45.5
Legitimacy	7	3.4	2	1.9	4	4.0	13	3.3
All others	15	13.4	21	20.0	4	4.0	40	10.1
Study sign-up	**325**	**13.1**	**182**	**11.2**	**274**	**16.1**	**781**	**13.4**
Access code	58	16.7	41	22.5	25	9.1	124	15.9
Registration procedures	155	47.7	79	43.4	145	52.9	379	48.5
Additional kits	45	13.9	37	20.3	49	17.9	131	16.8
Postcard^a^	20	6.2	9	5.0	17	6.2	46	5.9
Order confirmation	21	6.5	10	5.5	32	11.7	63	8.1
Website issues	23	7.1	2	1.1	2	0.7	27	3.5
All others	3	0.9	4	2.2	4	1.5	11	1.4
Test Kit	**853**	**34.5**	**457**	**28.1**	**526**	**30.9**	**1836**	**31.6**
Activation code	159	18.6	26	5.7	28	5.3	213	11.6
Missing/Wrong item	20	2.3	6	1.3	15	2.9	41	2.2
Survey	234	27.4	127	27.8	200	38.0	561	30.6
Lost or missing kit	159	18.6	95	20.8	99	18.8	353	19.2
Total or partial withdrawal	9	1.1	20	4.4	25	4.8	54	2.9
Sample collection issues	63	7.4	25	5.5	34	6.5	122	6.6
Return confirmation	133	15.6	125	27.4	86	16.4	344	18.7
Deadline	15	1.8	20	4.4	20	3.8	55	3.0
All others	61	7.2	13	2.8	19	3.6	93	5.1
Test Results	**626**	**25.3**	**572**	**35.2**	**359**	**21.1**	**1557**	**26.8**
Not received	597	95.4	542	94.8	332	92.5	1471	94.5
Different address	11	1.8	14	2.5	16	4.5	41	2.6
More testing information	7	1.1	5	0.9	7	2.0	19	1.2
Need more explanation	9	1.4	6	1.1	3	0.8	18	1.2
All others	2	0.3	5	0.9	1	0.3	8	0.5
Gift Card	**480**	**19.4**	**310**	**19.1**	**447**	**26.2**	**1237**	**21.3**
Not received (complete)	346	72.1	196	63.2	322	72.0	864	69.8
Not received (incomplete)^b^	94	19.6	70	22.6	45	10.1	209	16.9
Non-functional link	2	0.4	9	2.9	8	1.8	19	1.5
Redemption difficulty	28	5.8	27	8.7	56	12.5	111	9.0
All others	10	2.1	8	2.6	16	3.6	34	2.7

^a^Invitation letters were followed-up by reminder postcards printed with the same access code. In a few cases, participants mistook the postcard as a new invitation and reached out to ask whether their household could register for the study a second time

^b^Some participants mistook the registration form for the online survey and reached out to ask about the delivery of the study gift card(s)

### Communication materials and community outreach

A total of 14 communication materials were developed and refined with the input and feedback of the participating counties’ local health departments. Materials were available in all four study languages and could be further tailored to better meet the needs of target populations in each county. The CalScope study team also partnered with local health departments and engaged with 54 trusted community-based organizations and local partners to disseminate study outreach materials and increase study awareness and support. Community partners included COVID-19 equity task forces and local libraries. Some examples of unique outreach approaches included hosting virtual presentations, creating radio public service announcement scripts, and filming video testimonials with previous participants that could be shared on public health websites and social media platforms.

## Discussion

CalScope was the first state sponsored study undertaken at CDPH to estimate SARS-CoV-2 seroprevalence in California. The study was designed to collect representative survey data to complement the state’s existing surveillance efforts, reaching households which may have had less access to testing resources as well as the pediatric population. Over the course of three waves between April 2021 and August 2022, a total of 32,671 (4.9%) households registered for the study, with 25,488 (78.0%) households completing 25,314 adult and 6,474 child surveys. In addition, 23,396 (71.6%) households provided 23,376 adult and 4,603 child blood samples for testing using an entirely home-based test kit without any in-person contact between participants and study staff.

The greatest loss to follow-up occurred among households that ordered a test kit, with 22.3% failing to return the test kit. Moreover, 31.8% of households who ordered a child test kit failed to return the test kit, which was higher than the 22.3% loss to follow-up seen for adult test kits. Among test kits that were returned, almost 12.1% of child test kits had an inadequate blood sample collected, which suggests some persistent difficulties with blood collection among child participants. However, testing for a child within the household seemed widely acceptable, as 83.5% of households with children opted to order a child test kit and 46.1% had a child complete full participation in the study.

Several study design considerations were integral to the overall success of CalScope. First, a scalable and customizable data system designed through REDCap helped to manage this statewide study with a relatively small number of core staff. The security, use, and adaptability of REDCap for data collection and management is well documented [11, 12]. For CalScope, API integration created an enhanced participant experience by facilitating automated data exchanges with the network of vendor and partner systems. The ability to program logic for customized email and text messages also greatly reduced staff burden for sending communications and reminders.

Study accessibility for non-English speakers or those without internet access was addressed by using the IVR system, interpreters, and translated materials. IVR was a particularly valuable registration method, utilized by 4–8% of households for registration per wave. However, the 13.4% difference in survey completion and 10.8% difference in full participation between IVR and online-registered households indicates that the two options may not have provided equal participant experiences. Overall low utilization of non-English forms to complete registrations (3.7%), surveys (3.1%), and support requests (4.2%) also indicate that despite the inclusion of multiple considerations to increase accessibility, participation for non-English speaking households was lower than expected based on population demographics.

The infrastructure for tracking and follow-up of support requests allowed staff to quickly respond to participant questions, keeping them engaged and more likely to complete the study. Systematically documenting the types of support also allowed staff to continuously analyze common issues and concerns, resulting in improved study materials and processes between waves. Furthermore, having support staff directly reach participants by phone was especially helpful for survey completion reminders and troubleshooting.

Finally, recruiting participants from seven selected counties instead of the entire state helped to focus the communication and outreach around study participation. CalScope relied on partnerships with trusted community-based organizations and local health departments to disseminate tailored messaging about the study and encourage participation from invited residents. CalScope was featured in press releases, local news stories, and social media posts from official government accounts, which likely increased participants’ confidence around the legitimacy of the study. As a result, CalScope was able to meet study registration targets for most counties, with improvements seen even within counties where response rates were lower than expected.

## Conclusions

Despite some limitations, public health departments can enhance existing routine surveillance systems with remotely collected surveys and at-home serological testing data. Such data can improve population-based estimates of novel diseases and conditions that may be underreported. Data from such studies can also be used to improve forecast models and identify health disparities among specific populations that may benefit from increased attention and better resource allocation. Although these methods are not universally applicable, diseases with higher true prevalence and longer immunological responses that can be tested with DBS samples may benefit from adapting the study methods presented.

### Supplementary Information

Supplementary Material 1.

## Data Availability

The datasets used and/or analyzed during the current study are available from the corresponding author on reasonable request.

## Abbreviations

AC: Access Codes
API: Application Programming Interface
CDPH: California Department of Public Health
DBS: Dried Blood Spot
EM: External Module
IVR: Interactive Voice Response
sFTP: Secure File Transfer Protocol
S1: Spike 1
USPS: United States Postal Service
W1: Wave 1
W2: Wave 2
W3: Wave 3
