# Supplementary material for: CalScope: methodology and lessons learned for conducting a remote statewide SARS-CoV-2 seroprevalence study in California using an at-home dried blood spot collection kit and online survey
Source: BMC Med Res Methodol. 2024 May 27;24:120. doi: 10.1186/s12874-024-02245-y (PMC11131314; doi:10.1186/s12874-024-02245-y)
Supplement: Supplementary file 1 — Supplementary Material 1. [file 12874_2024_2245_MOESM1_ESM.zip › A. Invitation materials.pdf]

CA Department of Public Health
PO Box 997377 | MS 0500 | Sacramento, CA 95899-7377

California Department of PublicHealth

NON-PROFIT
ORGANIZATION
U.S. POSTAGE
PAID
PERMIT #1234
CITY, ST

Your household has been selected

Current Resident
<Address Line 1>
<Address Line 2>
<City>, <ST> <12345-6789>

Help combat COVID-19. Ayude a combatir el COVID-19 帮助抗击新型冠状病

CalScope

California Department of Public Health

You can help us learn more about COVID-19 in California!

Register at CalScope.org today using your unique access code:

<12345678>

Your household is invited to take part in a free, at-home, and anonymous COVID-19 antibody study. This will help the California Department of Public Health better understand the spread of the virus.

Many cases of COVID-19 go uncounted because people don't always have symptoms or get tested.

By testing for COVID-19 antibodies, this study will help us learn how many people in California have been infected with the virus in the past. It does not test for current COVID-19 infections.

Who can join: Up to 1 adult and 1 child (6 months to 17 years old) from your household.

How to take part: Just answer a survey online and take a finger-prick blood test at home.

To register for this study, just enter the unique access code below at **calscope.org**. You will then complete the quick online survey to order your test kit(s). Mail the kit back to the lab using the pre-paid mailer and get your test results by mail in 3–4 weeks.

You will get a $20 gift card for finishing the survey and a $20 gift card for the test kit that you send back. Get up to $40 per adult and $40 per child in a household.

All information you provide will be kept confidential.

*Know that your household was randomly picked from all addresses in your county. Personal information (like your name and birthday) will **not** be collected for this study. Please do not give this invitation to someone else — the access code on this letter is for your address only. If you want to join the study but don't want to take the blood test, you can just complete the online survey and get a $20 gift card.*

Get a free test for COVID-19 antibodies. Sign up at calscope.org or call 1-833-580-1333.
Questions? Call 1-833-580-1333 or email calscope@cdph.ca.gov

To join this study, please use your unique access code and **sign up within 5 days** after getting this letter.

Stanford Medicine · Alameda County Public Health Department · El Dorado County · Kern County Public Health Services Department · County of Los Angeles Public Health · County of Monterey Health Department · County of San Diego HHSA Live Well San Diego · Shasta County Health & Human Services Agency · gauss

Help combat COVID-19. Ayude a combatir el COVID-19 帮助抗击新型冠状病

CalScope

California Department of PublicHealth

¡Usted puede ayudarnos a obtener más información sobre la COVID-19 en California!

Invitamos a su hogar a participar en un estudio sobre los anticuerpos contra la COVID-19 **gratuito, en casa y anónimo.** Esto ayudará al Departamento de Salud Pública de California a comprender mejor la propagación del virus.

Muchos casos de COVID-19 no se cuentan porque las personas no siempre tienen síntomas o no se realizan la prueba de detección.

Como se realizarán pruebas de detección de los anticuerpos contra la COVID-19, este estudio nos permitirá saber cuántas personas en California tuvieron el virus anteriormente. No se detectan casos actuales de COVID-19.

Quiénes pueden participar: Hasta 1 adulto y 1 menor (6 meses a 17 años) de su hogar.

Cómo participar: Smplemente responda una encuesta en línea y hágase una prueba en casa, que consiste en extraer sangre mediante un pinchazo en el dedo.

Para registrarse en este estudio, simplemente ingrese el código de acceso único que se encuentra en la primera página, en **calscope.org**. Tendrá que completar una breve encuesta en línea para solicitar los kits de prueba. Envíe el kit de regreso al laboratorio con el sobre prepagado y reciba los resultados de sus pruebas por correo en tres o cuatro semanas.

Recibirá una tarjeta de regalo de $20 por completar la encuesta y una tarjeta de regalo de $20 por el kit de prueba que envíe de regreso. Reciba hasta $40 por adulto y $40 por menor en un hogar.

Se mantendrá la confidencialidad de toda la información que proporcione.

Tenga en cuenta que su hogar se eligió al azar entre todas las direcciones de su condado. ***No*** *se recopilará información personal (como su nombre y fecha de nacimiento) para este estudio. No le entregue esta invitación a otra persona. El código de acceso de esta carta es solo para su dirección. Si desea participar en el estudio, pero no quiere hacerse un análisis de sangre, puede completar la encuesta en línea y obtener una tarjeta de regalo de $20.*

Hágase una prueba gratuita de anticuerpos contra la COVID-19.
Regístrese en calscope.org o llame al 1-833-580-1333.
¿Preguntas? Llame al 1-833-580-1333 o escriba a calscope@cdph.ca.gov.

Para participar en este estudio, use su código de acceso único y **regístrese dentro de los cinco días** de haber recibido esta carta.

Stanford MEDICINE · Alameda County Public Health Department · El Dorado County California · Kern County Public Health Services Department · County of Los Angeles Public Health · County of Monterey Health Department · HHSA Live Well San Diego · Shasta County Health & Human Services Agency · gauss

Help combat COVID-19. Ayude a combatir el COVID-19 帮助抗击新型冠状病

CalScope

California Department of PublicHealth

Maaari kang makatulong sa dagdag na pag-aaral tungkol sa COVID-19 sa California!

Ang iyong sambahayan ay inaanyayahan na makisali sa isang **libre**, **sa loob-ng-tahanan**, at **walang pagkakakilanlan** na pag-aaral sa COVID-19 antibody. Ito ay makakatulong sa Departamento ng Pampublikong Pangkalusugan ng California na maintindihan nang mas mabuti ang pagkalat ng virus.

Maraming mga kaso ng COVID-19 ay hindi nabibilang dahil ang mga tao ay laging walang sintomas o hindi nagpasuri.

Sa pamamagitan ng pagpapasuri sa COVID-19 antibodies, makakatulong ang pag-aaral na ito upang malaman kung ilang mga tao sa California ang nahawaan ng virus sa nakaraan. Hindi ito nagsusuri ng kasalukuyang impeksyon ng COVID-19.

Sino ang maaaring sumali: Hanggang sa 1 adulto at 1 bata (6 na buwan hanggang 17 na taong gulang) mula sa iyong sambahayan.

Paano makilahok: Sagutin lamang ang online survey at kumuha ng sample ng dugo sa pamamagitan ng pagturok ng daliri na maaaring gawin sa loob ng bahay.

Upang mag-register para sa pagsusuring ito, ipasok lamang ang access code sa unang page, sa **calscope.org**. Kukumpletuhin mo ang online survey upang ma-order ang iyong (mga) test kit. Ipadala sa koreo ang kit pabalik sa lab gamit ang pre- paid mailer at kunin ang iyong resulta ng pagsusuri mula sa koreo sa loob ng 3-4 na linggo.

Ikaw ay makakuha ng isang $20 gift card para sa pagtapos ng survey at isang $20 gift card para sa test kit na ibinalik. Maaaring makakuha sa hanggang $40 kada adulto at $40 kada sambahayan.

Lahat ng impormasyon na iyong ibinigay ay maitatagong kumpidensyal.

*Alamin na ang iyong sambahayan ay napili ng sapalaran mula sa mga address sa iyong county. Ang personal na impormasyon (tulad ng iyong pangalan at kapanganakan) ay **hindi** makokolekta sa pag-aaral na ito. Huwag ibigay ang impormasyon na ito sa ibang tao – ang access code sa liham na ito ay para sa iyong address lamang. Kung nais na sumali sa pag-aaral pero hindi ninyo gusto na kumuha ng blood rest, maaaring kumpletuhin ang online survey at makakuha ng $20 gift card.*

Kumuha ng libreng pagsusuri ng COVID-19 antibodies.
Magpalista sa calscope.org o tumawag sa 1-833-580-1333.
May katanungan? Tumawag sa 1-833-580-1333 o email sa calscope@cdph.ca.gov.

Upang makasali sa pag-aaral na ito, gamitin ang iyong kakaibang access code at **magpalista sa loob ng 5 araw** pagkatapos na makuha ang liham na ito.

Stanford MEDICINE · Alameda County Public Health Department · El Dorado County · Kern County Public Health Services Department · County of Los Angeles Public Health · County of Monterey Health Department · HHSA Live Well San Diego · Shasta County Health & Human Services Agency · gauss

Help combat COVID-19. Ayude a combatir el COVID-19 帮助抗击新型冠状病

CalScope

California Department of PublicHealth

您可以帮助我们进一步了解有关加州 COVID–19 的更多信息！

特邀您的家庭参与一项免费的在家匿名 COVID–19 抗体研究。这将有助于加州公共卫生厅更好地了解病毒的传播。

许多 COVID–19 病例无法计数，因为患者并不总是有症状或接受检测。

这项研究将检测 COVID–19 抗体，能帮助我们了解过去有多少加州人感染过病毒，但不会检测现有的 COVID–19 感染。

合格参与者：每个家庭最多可有 1 个成人和 1 个孩子（6 个月至 17 岁）参与。

参与方式：只需在线回答一项调查并在家做指尖点刺验血即可。

要注册参与这项研究，只需在 **calscope.org** 输入第一页上的唯一访问代码。用邮资已付的回邮袋将试剂盒寄回检验室，检测结果会
在 3–4 周内寄回。

完成调查后将得到一张 20 美元的礼品卡，寄回检测试剂盒后将得到另一张 20 美元的礼品卡。家中每个成人和每个孩子各可最多得到 40 美元。

所有提供的信息都将保密。

*每个家庭都是从所在县的所有地址中随机挑选。这项研究**不会**收集个人信息（如姓名和生日）。切勿将此邀请信交给其他人，信中的访问码仅用于您的地址。如想参与研究但不想验血，可以只填写在线调查，并获得一张 20 美元的礼品卡。*

免费获得 COVID–19 抗体检测。在 calscope.org 网站上或致电 1–833–580–1333 注册
有疑问吗？致电 1–833–580–1333 或电邮 calscope@cdph.ca.gov

要参与这项研究，请使用您的唯一访问码在收到此信后 **5 天内注册.**

Stanford MEDICINE · Alameda County Public Health Department · El Dorado County California · Kern County Public Health Services Department · County of Los Angeles Public Health · County of Monterey Health Department · HHSA Live Well San Diego · Shasta County Health & Human Services Agency · gauss

CalScope

You can help us learn more about COVID-19 in California!

Get started today at calscope.org

Access code / Código de acceso / 访问代号:

<12345678>

California Department of PublicHealth

Stanford MEDICINE

gauss

CA Department of Public Health
PO Box 997377
MS 0500
Sacramento, CA 95899-7377

STANDARD
U.S. POSTAGE
PAID
CITY, ST
PERMIT NO. XXXX

Your household has been selected

Current Resident
<Address Line 1>
<Address Line 2>
<City>, <ST> <12345-6789>

Your household invite to join a statewide COVID-19 antibody study is pending.

Take a free test for COVID-19 antibodies using an at-home test kit and online survey. We will mail your results back in 3–4 weeks.

You will also get a $20 gift card for each survey and each test kit that is returned, or up to $80 per household.

La invitación para participar en un estudio estatal sobre los anticuerpos contra la COVID-19 está pendiente.

Hágase una prueba gratuita de anticuerpos contra la COVID-19 con un kit de prueba en casa y una encuesta en línea. Le enviaremos sus resultados por correo dentro de 3-4 semanas.

También recibirá una tarjeta de regalo de $20 por cada encuesta y cada kit de prueba que se entreguen, o hasta $80 por hogar.

Ang imbitasyon ng iyong sambahayan sa pagsali sa buong estadong pag-aaral ng COVID-19 antibody ay nakabinbin.

Kumuha ng libreng pagsusuri para sa COVID-19 antibodies gamit ang isang at-home test kit at online survey. Ipapadala namin ang mga resulta ng 3-4 na linggo.

Makakakuha ka rin ng isang $20 gift card para sa bawat survey at bawat test kit na naibalik, o hanggang sa $80 kada sambahayan.

期待您的家庭参与一项全州 COVID–19 抗体研究。

使用家用检测试剂盒和在线调查免费接受一项 COVID–19 抗体检测。检测结果将在 3–4 周内寄回。

每填写一个调查和寄一个检测试剂盒都将获得一张 20 美元的礼品卡，每家最多可得 80 美元。

Register today at CalScope.org using the access code <12345678> or call 1-833-580-1333.
