# Supplementary material for: CalScope: methodology and lessons learned for conducting a remote statewide SARS-CoV-2 seroprevalence study in California using an at-home dried blood spot collection kit and online survey
Source: BMC Med Res Methodol. 2024 May 27;24:120. doi: 10.1186/s12874-024-02245-y (PMC11131314; doi:10.1186/s12874-024-02245-y)
Supplement: Supplementary file 1 — Supplementary Material 1. [file 12874_2024_2245_MOESM1_ESM.zip › B. Test result letter.pdf]

CalScope

CDPH California Department of Public Health

<R1_header>

Collection Date/Fecha de colección: **<R1_collection_date>**

Result Date/Fecha de resultados: **<R1_result_date>**

**The results of your COVID-19 antibody test are below.**	**Los resultados de su prueba de anticuerpos para COVID-19 están a continuación.**
Please read the attached information sheet for details about the meaning of these tests, or go to www.calscope.org for more information.<br><br>**YOU SHOULD NOT CHANGE YOUR BEHAVIOR BASED ON THE RESULTS OF THIS TEST. PLEASE CONTINUE TO FOLLOW THE RECOMMENDATIONS OF YOUR COUNTY HEALTH DEPARTMENT.**<br><br>*Thank you for participating in CalScope. By taking part in this study, you have helped us better understand how COVID-19 has affected your community and California.*	Lea la hoja de información adjunta para conocer la importancia de estas pruebas, o visite www.calscope.org para más información.<br><br>**NO DEBE CAMBIAR SU COMPORTAMIENTO BASÁNDOSE EN LOS RESULTADOS DE ESTA PRUEBA. CONTINÚE SIGUIENDO LAS RECOMENDACIONES DEL DEPARTAMENTO DE SALUD DE SU CONDADO.**<br><br>*Gracias por participar en el estudio de CalScope. Al participar en este estudio, nos ha ayudado a entender mejor cómo elCOVID-19 ha afectado a su comunidad y a California.*

**YOUR TEST RESULTS**	**RESULTADOS DE SU PRUEBA**
**Antibodies present**<br><br>*Possible past infection, vaccination, or exposure to the virus that causes COVID-19 (SARS-CoV-2).*	**Presencia de anticuerpos**<br><br>*Posible infección pasada, vacunación o exposición al virus que causa el COVID-19 (SARS-CoV-2).*

**ADAP-SARS-CoV-2 Total Antibody Assay tests for antibodies against the spike (S1) protein of the SARS-CoV-2 virus. For more information, visit Enable Biosciences https://www.enablebiosciences.com.*

**El ensayo de anticuerpos totales ADAP-SARS-CoV-2 prueba para anticuerpos contra la proteína de pico (S1) del virus SARSCoV-2. Para obtener más información, visite Enable Biosciences https://www.enablebiosciences.com.*

Alameda County Public Health Department · El Dorado County California · Kern County Public Health Services Department · County of Los Angeles Public Health · County of Monterey Health Department · County of San Diego HHSA Live Well San Diego · Shasta County Health & Human Services Agency

DAP

CalScope

California Department of Public Health

<R1_header>

Collection Date/Fecha de colección: **<R1_collection_date>**

Result Date/Fecha de resultados: **<R1_result_date>**

**The results of your COVID-19 antibody test are below.**	**Los resultados de su prueba de anticuerpos para COVID-19 están a continuación.**
Please read the attached information sheet for details about the meaning of these tests, or go to www.calscope.org for more information.<br><br>**YOU SHOULD NOT CHANGE YOUR BEHAVIOR BASED ON THE RESULTS OF THIS TEST. PLEASE CONTINUE TO FOLLOW THE RECOMMENDATIONS OF YOUR COUNTY HEALTH DEPARTMENT.**<br><br>*Thank you for participating in CalScope. By taking part in this study, you have helped us better understand how COVID-19 has affected your community and California.*	Lea la hoja de información adjunta para conocer la importancia de estas pruebas, o visite www.calscope.org para más información.<br><br>**NO DEBE CAMBIAR SU COMPORTAMIENTO BASÁNDOSE EN LOS RESULTADOS DE ESTA PRUEBA. CONTINÚE SIGUIENDO LAS RECOMENDACIONES DEL DEPARTAMENTO DE SALUD DE SU CONDADO.**<br><br>*Gracias por participar en el estudio de CalScope. Al participar en este estudio, nos ha ayudado a entender mejor cómo el COVID-19 ha afectado a su comunidad y a California.*

**YOUR TEST RESULTS**	**RESULTADOS DE SU PRUEBA**
**Antibodies absent**<br><br>*No evidence of past infection, vaccination, or exposure to the virus that causes COVID-19 (SARS-CoV-2).*	**Ausencia de anticuerpos**<br><br>*No hay evidencia de infección, vacunación, o exposición pasada al virus que causa el COVID-19 (SARS-CoV-2).*

**ADAP-SARS-CoV-2 Total Antibody Assay tests for antibodies against the spike (S1) protein of the SARS-CoV-2 virus. For more information, visit Enable Biosciences https://www.enablebiosciences.com.*

**El ensayo de anticuerpos totales ADAP-SARS-CoV-2 prueba para anticuerpos contra la proteína de pico (S1) del virus SARSCoV-2. Para obtener más información, visite Enable Biosciences https://www.enablebiosciences.com.*

Alameda County Public Health Department · El Dorado County California · Kern County Public Health Services Department · County of Los Angeles Public Health · County of Monterey Health Department · HHSA Live Well San Diego · Shasta County Health & Human Services Agency

<R1_header>

Collection Date/Fecha de colección: <R1_collection_date>

Result Date/Fecha de resultados: <R1_result_date>

The results of your COVID-19 antibody test are below.

Please read the attached information sheet for details about the meaning of these tests, or go to www.calscope.org for more information.

YOU SHOULD NOT CHANGE YOUR BEHAVIOR BASED ON THE RESULTS OF THIS TEST. PLEASE CONTINUE TO FOLLOW THE RECOMMENDATIONS OF YOUR COUNTY HEALTH DEPARTMENT.

Thank you for participating in CalScope. By taking part in this study, you have helped us better understand how COVID-19 has affected your community and California.

Los resultados de su prueba de anticuerpos para COVID-19 están a continuación.

Lea la hoja de información adjunta para conocer la importancia de estas pruebas, o visite www.calscope.org para más información.

NO DEBE CAMBIAR SU COMPORTAMIENTO BASÁNDOSE EN LOS RESULTADOS DE ESTA PRUEBA. CONTINÚE SIGUIENDO LAS RECOMENDACIONES DEL DEPARTAMENTO DE SALUD DE SU CONDADO.

Gracias por participar en el estudio de CalScope. Al participar en este estudio, nos ha ayudado a entender mejor cómo el COVID-19 ha afectado a su comunidad y a California.

YOUR TEST RESULTS

Indeterminate

Test result was unclear because of poor sample quality, too little sample, or other issue.

RESULTADOS DE SU PRUEBA

Indeterminado

El resultado de la prueba no fue claro porque la cantidad de la muestra era insuficiente, su calidad no era buena o hubo otro problema.

**ADAP-SARS-CoV-2 Total Antibody Assay tests for antibodies against the spike (S1) protein of the SARS-CoV-2 virus. For more information, visit Enable Biosciences https://www.enablebiosciences.com.*

**El ensayo de anticuerpos totales ADAP-SARS-CoV-2 prueba para anticuerpos contra la proteína de pico (S1) del virus SARSCoV-2. Para obtener más información, visite Enable Biosciences https://www.enablebiosciences.com.*

DAI

CalScope

CDPH California Department of Public Health

<R1_header>

Collection Date/Petsa ng Koleksyon: <R1_collection_date>

Result Date/Petsa ng Resulta: <R1_result_date>

The results of your COVID-19 antibody test are below.	Ang mga resulta ng iyong COVID-19 antibody test ay sa ibaba.
Please read the attached information sheet for details about the meaning of these tests, or go to www.calscope.org for more information.<br><br>**YOU SHOULD NOT CHANGE YOUR BEHAVIOR BASED ON THE RESULTS OF THIS TEST. PLEASE CONTINUE TO FOLLOW THE RECOMMENDATIONS OF YOUR COUNTY HEALTH DEPARTMENT.**<br><br>*Thank you for participating in CalScope. By taking part in this study, you have helped us better understand how COVID-19 has affected your community and California.*	Pakibasa ang nakalakip na pliyego ng impormasyon para sa mga detalye tungkol sa ibig sabihin ng mga pagsusuri na ito, o pumunta sa www.calscope.org para sa karagdagang impormasyon.<br><br>**HINDI MO DAPAT BAGUHIN ANG IYONG GAWI BASE SA MGA RESULTA NG PAGSUSURING ITO. PATULOY NA SUNDIN ANG MGA REKOMENDASYON NG IYONG DEPARTAMENTO NG PANGKALASUGAN NG COUNTY.**<br><br>*Salamat sa pakikilahok sa CalScope. Sa pamamagitan ng pakikibahagi sa pag-aaral na ito, natulungan mo kami na maintindihan nang mas mabuti kung paano nakakaapekto ang COVID-19 sa komunidad at sa California.*

YOUR TEST RESULTS	ANG IYONG RESULTA NG PAGSUSURI
**Antibodies present**<br><br>*Possible past infection, vaccination, or exposure to the virus that causes COVID-19 (SARS-CoV-2).*	**Mayroong antibodies**<br><br>*Posibleng ngkaroon ng impeksyon, bakunasyon o may pagkakalantad sa virus na sanhi ng COVID-19 (SARS-CoV-2).*

**ADAP-SARS-CoV-2 Total Antibody Assay tests for antibodies against the spike (S1) protein of the SARS-CoV-2 virus. For more information, visit Enable Biosciences https://www.enablebiosciences.com.*

**Ang ADAP-SARS-CoV-2 Total Antibody Assay na pagsusuri para sa antibodies laban sa spike (S1) protein ng SARS-CoV-2 na virus. Para sa karagdagang impormasyon, bumisita sa Enable Biosciences https://www.enablebiosciences.com.*

Alameda County Public Health Department · El Dorado County California · Kern County Public Health Services Department · County of Los Angeles Public Health · County of Monterey Health Department, Nationally Accredited for Providing Quality Health Services · County of San Diego HHSA, Live Well San Diego · Shasta County Health & Human Services Agency

TAP

CalScope

California Department of Public Health

<R1_header>

Collection Date/Petsa ng Koleksyon: **<R1_collection_date>**

Result Date/Petsa ng Resulta: **<R1_result_date>**

The results of your COVID-19 antibody test are below.

Ang mga resulta ng iyong COVID-19 antibody test ay sa ibaba.

Please read the attached information sheet for details about the meaning of these tests, or go to www.calscope.org for more information.<br><br>**YOU SHOULD NOT CHANGE YOUR BEHAVIOR BASED ON THE RESULTS OF THIS TEST. PLEASE CONTINUE TO FOLLOW THE RECOMMENDATIONS OF YOUR COUNTY HEALTH DEPARTMENT.**<br><br>*Thank you for participating in CalScope. By taking part in this study, you have helped us better understand how COVID-19 has affected your community and California.*	Pakibasa ang nakalakip na pliyego ng impormasyon para sa mga detalye tungkol sa ibig sabihin ng mga pagsusuri na ito, o pumunta sa www.calscope.org para sa karagdagang impormasyon.<br><br>**HINDI MO DAPAT BAGUHIN ANG IYONG GAWI BASE SA MGA RESULTA NG PAGSUSURING ITO. PATULOY NA SUNDIN ANG MGA REKOMENDASYON NG IYONG DEPARTAMENTO NG PANGKALASUGAN NG COUNTY.**<br><br>*Salamat sa pakikilahok sa CalScope. Sa pamamagitan ng pakikibahagi sa pag-aaral na ito, natulungan mo kami na maintindihan nang mas mabuti kung paano nakakaapekto ang COVID-19 sa komunidad at sa California.*

YOUR TEST RESULTS	ANG IYONG RESULTA NG PAGSUSURI
**Antibodies absent**<br><br>*No evidence of past infection, vaccination, or exposure to the virus that causes COVID-19 (SARS-CoV-2).*	**Walang antibodies**<br><br>*Walang ebidensya ng nakaraang impeksyon, bakunasyon, o pagkalantad sa virus na sanhi ng COVID-19 (SARS-CoV-2).*

**ADAP-SARS-CoV-2 Total Antibody Assay tests for antibodies against the spike (S1) protein of the SARS-CoV-2 virus. For more information, visit Enable Biosciences https://www.enablebiosciences.com.*

**Ang ADAP-SARS-CoV-2 Total Antibody Assay na pagsusuri para sa antibodies laban sa spike (S1) protein ng SARS-CoV-2 na virus. Para sa karagdagang impormasyon, bumisita sa Enable Biosciences https://www.enablebiosciences.com.*

Alameda County Public Health Department · El Dorado County California · Kern County Public Health Services Department · County of Los Angeles Public Health · County of Monterey Health Department, Nationally Accredited for Providing Quality Health Services · County of San Diego HHSA, Live Well San Diego · Shasta County Health & Human Services Agency

TAA

CalScope

CDPH California Department of Public Health

<R1_header>

Collection Date/Petsa ng Koleksyon: **<R1_collection_date>**

Result Date/Petsa ng Resulta: **<R1_result_date>**

**The results of your COVID-19 antibody test are below.**	**Ang mga resulta ng iyong COVID-19 antibody test ay sa ibaba.**
Please read the attached information sheet for details about the meaning of these tests, or go to www.calscope.org for more information.<br><br>**YOU SHOULD NOT CHANGE YOUR BEHAVIOR BASED ON THE RESULTS OF THIS TEST. PLEASE CONTINUE TO FOLLOW THE RECOMMENDATIONS OF YOUR COUNTY HEALTH DEPARTMENT.**<br><br>*Thank you for participating in CalScope. By taking part in this study, you have helped us better understand how COVID-19 has affected your community and California.*	Pakibasa ang nakalakip na pliyego ng impormasyon para sa mga detalye tungkol sa ibig sabihin ng mga pagsusuri na ito, o pumunta sa www.calscope.org para sa karagdagang impormasyon.<br><br>**HINDI MO DAPAT BAGUHIN ANG IYONG GAWI BASE SA MGA RESULTA NG PAGSUSURING ITO. PATULOY NA SUNDIN ANG MGA REKOMENDASYON NG IYONG DEPARTAMENTO NG PANGKALASUGAN NG COUNTY.**<br><br>*Salamat sa pakikilahok sa CalScope. Sa pamamagitan ng pakikibahagi sa pag-aaral na ito, natulungan mo kami na maintindihan nang mas mabuti kung paano nakakaapekto ang COVID-19 sa komunidad at sa California.*

**YOUR TEST RESULTS**	**ANG IYONG RESULTA NG PAGSUSURI**
**Indeterminate**<br><br>*Test result was unclear because of poor sample quality, too little sample, or other issue.*	**Intermedya**<br><br>*Ang resulta ng pagsusuri ay hindi malinaw dahil sa mababang kalidad ng sample, masyadong maliit na sample, o ibang isyu.*

**ADAP-SARS-CoV-2 Total Antibody Assay tests for antibodies against the spike (S1) protein of the SARS-CoV-2 virus. For more information, visit Enable Biosciences https://www.enablebiosciences.com.*

**Ang ADAP-SARS-CoV-2 Total Antibody Assay na pagsusuri para sa antibodies laban sa spike (S1) protein ng SARS-CoV-2 na virus. Para sa karagdagang impormasyon, bumisita sa Enable Biosciences https://www.enablebiosciences.com.*

Alameda County Public Health Department · El Dorado County California · Kern County Public Health Services Department · County of Los Angeles Public Health · County of Monterey Health Department — Nationally Accredited for Providing Quality Health Services · County of San Diego HHSA — Live Well San Diego · Shasta County Health & Human Services Agency

<R1_header>

Collection Date/采血日期: **<R1_collection_date>**

Result Date/结果日期: **<R1_result_date>**

The results of your COVID-19 antibody test are below.

您的 COVID–19 抗体检测结果如下。

Please read the attached information sheet for details about the meaning of these tests, or go to www.calscope.org for more information.<br><br>**YOU SHOULD NOT CHANGE YOUR BEHAVIOR BASED ON THE RESULTS OF THIS TEST. PLEASE CONTINUE TO FOLLOW THE RECOMMENDATIONS OF YOUR COUNTY HEALTH DEPARTMENT.**<br><br>*Thank you for participating in CalScope. By taking part in this study, you have helped us better understand how COVID-19 has affected your community and California.*	请阅读附件中的信息表，以获取有关这些检测含义的详细信息，或访问 www.calscope.org 获取更多信息。<br><br>**不应因检测结果而改变行为。应继续遵循所在县卫生部门的建议。**<br><br>*感谢您参与 CalScope 研究。您参与这项研究的奉献有助于更好地了解 COVID–19 如何影响您所在社区和整个加州。*

YOUR TEST RESULTS	您的检测结果
**Antibodies present**	**有抗体**
Possible past infection, vaccination, or exposure to the virus that causes COVID-19 (SARS-CoV-2).	*过去可能感染过,疫苗种过,或接触过引起 COVID–19 (SARS–CoV–2) 的病毒。*

*ADAP-SARS-CoV-2 Total Antibody Assay tests for antibodies against the spike (S1) protein of the SARS-CoV-2 virus. For more information, visit Enable Biosciences https://www.enablebiosciences.com.

*针对 SARS–CoV–2 病毒刺突 (S1) 蛋白抗体的 ADAP–SARS–CoV–2 Total Antibody Assay 检测。如需更多信息，请访问 Enable Biosciences https://www.enablebiosciences.com。

CAP

<R1_header>

Collection Date/采血日期: **<R1_collection_date>**

Result Date/结果日期: **<R1_result_date>**

The results of your COVID-19 antibody test are below.

您的 COVID–19 抗体检测结果如下。

Please read the attached information sheet for details about the meaning of these tests, or go to www.calscope.org for more information.<br><br>**YOU SHOULD NOT CHANGE YOUR BEHAVIOR BASED ON THE RESULTS OF THIS TEST. PLEASE CONTINUE TO FOLLOW THE RECOMMENDATIONS OF YOUR COUNTY HEALTH DEPARTMENT.**<br><br>*Thank you for participating in CalScope. By taking part in this study, you have helped us better understand how COVID-19 has affected your community and California.*	请阅读附件中的信息表，<br>以获取有关这些检测含义的详细信息，<br>或访问 www.calscope.org 获取更多信息。<br><br>**不应因检测结果而改变行为。**<br>**应继续遵循所在县卫生部门的建议。**<br><br>*感谢您参与 CalScope 研究。*<br>*您参与这项研究的奉献*<br>*有助于更好地了解 COVID–19*<br>*如何影响您所在社区和整个加州。*

**YOUR TEST RESULTS**	**您的检测结果**
**Antibodies absent**	**没有抗体**
No evidence of past infection, vaccination, or exposure to the virus that causes COVID-19 (SARS-CoV-2).	*没有证据表明过去感染, 疫苗种过,或接触过引起 COVID–19 (SARS–CoV–2) 的病毒。*

**ADAP-SARS-CoV-2 Total Antibody Assay tests for antibodies against the spike (S1) protein of the SARS-CoV-2 virus. For more information, visit Enable Biosciences https://www.enablebiosciences.com.*

**针对 SARS–CoV–2 病毒刺突 (S1) 蛋白抗体的 ADAP–SARS–CoV–2 Total Antibody Assay 检测。如需更多信息，请访问 Enable Biosciences https://www.enablebiosciences.com。*

<R1_header>

Collection Date/采血日期: <R1_collection_date>

Result Date/结果日期: <R1_result_date>

The results of your COVID-19 antibody test are below.

您的 COVID–19 抗体检测结果如下。

Please read the attached information sheet for details about the meaning of these tests, or go to www.calscope.org for more information.<br><br>**YOU SHOULD NOT CHANGE YOUR BEHAVIOR BASED ON THE RESULTS OF THIS TEST. PLEASE CONTINUE TO FOLLOW THE RECOMMENDATIONS OF YOUR COUNTY HEALTH DEPARTMENT.**<br><br>*Thank you for participating in CalScope. By taking part in this study, you have helped us better understand how COVID-19 has affected your community and California.*	请阅读附件中的信息表，<br>以获取有关这些检测含义的详细信息，<br>或访问 www.calscope.org 获取更多信息。<br><br>**不应因检测结果而改变行为。**<br>**应继续遵循所在县卫生部门的建议。**<br><br>*感谢您参与 CalScope 研究。*<br>*您参与这项研究的奉献*<br>*有助于更好地了解 COVID–19*<br>*如何影响您所在社区和整个加州。*

**YOUR TEST RESULTS**	**您的检测结果**
**Indeterminate**	**不定**
Test result was unclear because of poor sample quality, too little sample, or other issue.	*由于样品质量差、样品太少或其他问题，检测结果不清楚。*

**ADAP-SARS-CoV-2 Total Antibody Assay tests for antibodies against the spike (S1) protein of the SARS-CoV-2 virus. For more information, visit Enable Biosciences https://www.enablebiosciences.com.*

**针对 SARS–CoV–2 病毒刺突 (S1) 蛋白抗体的 ADAP–SARS–CoV–2 Total Antibody Assay 检测。如需更多信息，请访问 Enable Biosciences https://www.enablebiosciences.com。*

CAI
