# Supplementary material for: CalScope: methodology and lessons learned for conducting a remote statewide SARS-CoV-2 seroprevalence study in California using an at-home dried blood spot collection kit and online survey
Source: BMC Med Res Methodol. 2024 May 27;24:120. doi: 10.1186/s12874-024-02245-y (PMC11131314; doi:10.1186/s12874-024-02245-y)
Supplement: Supplementary file 1 — Supplementary Material 1. [file 12874_2024_2245_MOESM1_ESM.zip › C. Results interpretation letter.pdf]

WHAT YOUR TEST RESULTS MEAN / SIGNIFICADO DE LOS RESULTADOS DE SU PRUEBA

Understanding COVID-19 Antibody Test Results

What is the COVID-19 antibody test?

This antibody test looks for coronavirus (the virus that causes COVID-19, called SARS -CoV-2) antibodies in your blood. Your body's immune system makes antibodies after you have been infected with a virus. Antibodies stay in your blood even after the virus is gone. Your body also makes antibodies after you have had a vaccine or shot. We do not know how long coronavirus antibodies stay in the blood.

How is this antibody test different from other COVID-19 tests?

This test looks for antibodies against the spike (S1) protein of the SARS-CoV-2 virus. The antibody test **does not** tell you if you are infected with the coronavirus (SARS-CoV-2) now. You do not need to take any additional steps after receiving the results of your antibody test.

This test is **not** a nasal swab, oral swab, or saliva test, which looks for the actual coronavirus (SARS-CoV-2). A positive nasal swab, oral swab, or saliva test means you are infected with the virus that causes COVID-19, and you may be able to spread it to others. If you have a positive test, you should isolate from others to prevent spreading the virus to them.

My test result says, "antibodies absent." What does that mean?

This negative test result can mean one of the following:

1. You have never been infected with the coronavirus (SARS-CoV-2) before. You can still get the virus in the future.
2. You got the virus in the last two weeks and your immune system has not had enough time to make antibodies yet.
3. You may have had the virus or gotten a vaccine in the past, but this test did not find any of the antibodies in your blood.
4. You may have gotten a vaccine but your body has not had enough time to make antibodies yet.

The test may be wrong for other reasons besides being done too early or too late after infection or vaccination. This kind of error is uncommon (only happens about 1 time out of 100 tests). Because this is the first time these kinds of tests have been used by people in their homes, we can't be 100% sure of the exact accuracy of these tests.

Interpretación de los resultados de la prueba de anticuerpos para COVID-19

¿Qué es la prueba de anticuerpos para COVID-19?

Esta prueba de anticuerpos busca los anticuerpos del coronavirus (el virus que causa el COVID-19, llamado SARS-CoV-2) en la sangre. El sistema inmunitario de su cuerpo crea anticuerpos después de que se ha infectado con un virus, y estos anticuerpos permanecen en la sangre incluso después de que el virus desaparece. El cuerpo también produce anticuerpos después de haber recibido una vacuna o una inyección. No sabemos cuánto tiempo permanecen los anticuerpos del coronavirus en la sangre.

¿En qué se diferencia esta prueba de anticuerpos de otras pruebas de COVID-19?

La prueba utilizada por el laboratorio busca anticuerpos contra la proteína de pico (S1) del virus SARS-CoV-2. La prueba de anticuerpos **no** le dice si actualmente está infectado con el coronavirus (SARS-CoV-2). No necesita adoptar nuevas medidas después de recibir los resultados de su prueba de anticuerpos.

Esta prueba **no** es una prueba de hisopado nasal, oral, o de saliva que busca el coronavirus (SARS-CoV-2). Un resultado positivo en la prueba de hisopado nasal, oral, o de saliva significa que está infectado con el virus que causa el COVID-19 y que puede contagiar a otras personas. Si su prueba es positiva, debe aislarse de los demás para evitar contagiarles el virus.

El resultado de mi prueba dice, "ausencia de anticuerpos." ¿Qué significa?

Este resultado negativo puede significar una de las siguientes razones:

1. Nunca antes ha sido infectado con el coronavirus (SARS-CoV-2). Todavía puede contraer el virus en el futuro.
2. Ha contraído el virus en las últimas dos semanas y su sistema inmunitario no ha tenido suficiente tiempo para crear anticuerpos.
3. Es posible que haya tenido el virus o haya recibido una vacuna en el pasado, pero en esta prueba no se encontró ningún anticuerpo presente en la sangre.
4. Es posible que haya recibido la vacuna, pero su cuerpo aún no ha tenido suficiente tiempo para crear anticuerpos.

La prueba puede ser incorrecta por otras razones además de haberse realizado demasiado pronto o demasiado tarde después de la infección o la vacuna. Este tipo de error es poco común (solo ocurre 1 vez de cada 100 pruebas). Debido a que esta es la primera vez que este tipo de pruebas es utilizado por personas en sus hogares, aún no podemos estar 100 % seguros de su exactitud.

My test result says, "antibodies present." What does that mean?

This positive test result can mean one of the following:

1. You were infected with the coronavirus (SARS-CoV-2) at some point in the past.
2. You may have had a COVID-19 vaccine.
3. The test may be wrong and you have never had the virus before. This kind of error is uncommon (only happens about 5 to 15 times out of 100 tests). Because this is the first time these tests have been used by people in their homes, we can't be 100% sure of the exact accuracy of these tests.

You should not change your behavior based on the results of this test.

El resultado de mi prueba dice "presencia de anticuerpos." ¿Qué significa?

Este resultado positivo puede significar una de las siguientes razones:

1. Usted estuvo infectado con el coronavirus (SARS-CoV-2) en algún momento del pasado.
2. Es posible que haya recibido la vacuna contra la COVID-19.
3. La prueba puede ser incorrecta y nunca antes haber tenido el virus. Este tipo de error es poco común (solo ocurre entre 5 y 15 veces de cada 100 pruebas). Debido a que esta es la primera vez que estas pruebas son utilizadas por personas en sus hogares, aún no podemos estar 100 % seguros de su exactitud.

No debe cambiar su comportamiento basándose en los resultados de esta prueba.

Is this test FDA-approved or cleared?

No. This antibody blood test for use at home has been designed and tested for public health purposes only and has not been approved or reviewed by the U.S. Food and Drug Administration (FDA). This means this test should not be used to make medical decisions. For more information on the test, visit https://www.enablebiosciences.com.

¿Esta prueba está aprobada o autorizada por la FDA?

No. Esta prueba de anticuerpos en sangre para uso en el hogar ha sido diseñada y probada con fines de salud pública únicamente y no ha sido aprobada o revisada por la Administración de Alimentos y Medicamentos (FDA) de los Estados Unidos. Esto significa que esta prueba no debe utilizarse para tomar decisiones médicas. Para obtener más información sobre la prueba, visite https://www.enablebiosciences.com.

How can I continue protecting myself and others from COVID-19?

1. Get vaccinated and boosted regardless of your test results.
2. Get tested if you're sick or if you've been exposed.
3. Wear a mask where required and on public transit.
4. Cover your coughs and sneezes with a tissue and wash your hands after you do.
5. Avoid poorly ventilated spaces.
6. Stay away from work, school, or other people if you become sick with respiratory symptoms like cough and fever.

For more current recommendations, visit https://covid19.ca.gov/safely-reopening/#what-to-do-now.

¿Cómo puedo seguir protegiéndome y protegiendo a los demás del COVID-19?

1. Vacúnese y reciba su dosis de refuerzo independientemente de los resultados de la prueba.
2. Hágase una prueba de detección si está enfermo o si ha estado expuesto.
3. Use una mascarilla donde sea necesario y en transporte público.
4. Cúbrase al toser y estornudar con un pañuelo de papel y lávese las manos después de hacerlo.
5. Evite los espacios mal ventilados.
6. Manténgase alejado del trabajo, la escuela u otras personas si se enferma con síntomas respiratorios como tos y fiebre.

Para más recomendaciones actuales, visite https://covid19.ca.gov/safely-reopening/#what-to-do-now.

No matter your result of this antibody test, you should continue to follow all other recommendations from your County Public Health Department.

For current recommendations for your county, visit https://covid19.ca.gov/get-local-information/

Sin importar el resultado de esta prueba de anticuerpos, debe continuar cumpliendo todas las demás recomendaciones del Departamento de Salud Pública de su condado.

Para conocer las recomendaciones actuales para su condado, visite https://covid19.ca.gov/get-local-information/
