# Supplementary material for: CalScope: methodology and lessons learned for conducting a remote statewide SARS-CoV-2 seroprevalence study in California using an at-home dried blood spot collection kit and online survey
Source: BMC Med Res Methodol. 2024 May 27;24:120. doi: 10.1186/s12874-024-02245-y (PMC11131314; doi:10.1186/s12874-024-02245-y)
Supplement: Supplementary file 1 — Supplementary Material 1. [file 12874_2024_2245_MOESM1_ESM.zip › D. Test Kit Letter.pdf]

READ ME FIRST

Follow the steps below to complete your survey(s) and collect your blood sample(s).
A child participating in the study will need help from an adult.

Before you begin, find the sticker on the inside of your test box and copy your 6-character activation code you will use for your online survey:

C ___ ___ ___ ___ ___

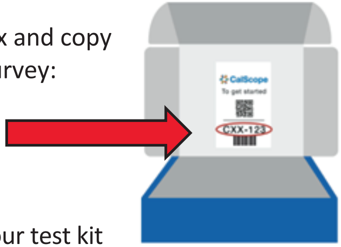

Step 1: Access & complete your online survey(s)*

- Go to CalScope.org or scan the QR code on the inside of your test kit with your smartphone camera. Click on "I GOT A TEST KIT".
- Enter your activation code and 5-digit ZIP code to activate your test kit and complete your online survey(s).
- At the end of the survey(s), choose if you want to receive your gift card(s) by email or mail. Mail deliveries can take up to 4 weeks.

Call us if you don't have Internet or need any help with this part!

Step 2: Collect your blood sample(s)

- Follow the directions in the folded *Step-by-Step Guide,* or view video instructions online at CalScope.org to collect the finger-prick blood sample(s).

Step 3: Mail back your test kit(s) within 2 weeks using the pre-paid USPS mailer bag.

What's next? Get your COVID-19 antibody test results by USPS mail 6-8 weeks after the lab receives your blood samples.

Questions? Email **calscope@cdph.ca.gov** or call **1-833-580-1333.**

KEEP THIS FOR YOUR RECORDS

LÉAME PRIMERO

Siga los pasos para completar su(s) encuesta(s) y tomar su(s) muestra(s) de sangre.
Los menores que participen en el estudio necesitarán ayuda de un adulto.

Antes de empezar, busque la pegatina que se encuentra dentro de la caja de las pruebas y escriba el código de activación de 6 caracteres que utilizará para completar la encuesta en línea:

C __ __ __ __ __

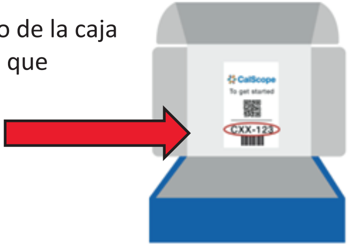

Paso 1: Acceda y complete la(s) encuesta(s) en línea*

- Visite CalScope.org o escanee el código QR en el interior del kit de prueba con la cámara de su teléfono. Haga clic en "Tengo un kit de prueba".
- Ingrese el código de activación y su código postal de 5-dígitos para activar el kit de prueba y completar su(s) encuesta(s) en línea.
- Al final de la(s) encuesta(s), elija si desea recibir su(s) tarjeta(s) de regalo por correo electrónico o postal. Las entregas por correo postal pueden demorar 4 semanas.

¡Llámenos si no tiene Internet o necesita ayuda con esto paso!

Paso 2: Tome su(s) muestra(s) de sangre

- Siga las instrucciones en la *Guía Paso-a-Paso* o vea el video de instrucciones en CalScope.org para tomar la(s) muestra(s) de sangre.

Paso 3: Envíe por correo postal su(s) kit(s) de prueba dentro de 2 semanas con la bolsa de envío del USPS prepaga.

¿Qué sigue? Reciba los resultados de su prueba de anticuerpos de COVID-19 por correo postal entre 6-8 semanas después de que el laboratorio reciba sus muestras de sangre.

¿Tiene preguntas? Envíe un correo electrónico a **calscope@cdph.ca.gov** o llame al **1-833-580-1333**.

GUARDE ESTA TARJETA PARA SUS REGISTROS

BASAHIN MO MUNA AKO

Sundin ang mga hakbang sa ibaba upang kumpletuhin ang iyong (mga) survey at kumolekta ng iyong (mga) blood sample. Ang isang bata na nakikilahok sa pag-aaral ay kailangan ng tulong mula sa isang adulto.

Bago ka magsimula, hanapin ang sticker sa ilalim ng iyong test box at kopyahin ang iyong 6-karakter na activation code na iyong gagamitin para sa iyong online na pagsisiyasat:

C __ __ __ __ __

Hakbang 1: I-access & kumpletuhin ang iyong (mga) online survey*

- Magpunta sa CalScope.org o i-scan ang QR code sa loob ng iyong test kit gamit ang iyong smartphone na camera. I-click ang "I GOT A TEST KIT".
- Ipasok ang iyong activation at 5-numero na ZIP code upang gawing aktibo ang iyong test kit at kumpletuhin ang iyong (mga) online survey.
- Sa huli ng iyong (mga) survey, pumili kung nais mo na tumanggap ng iyong (mga) gift card sa pamamagitan ng email o sa koreo. Ang pagpapadala sa koreo ay maaaring umabot sa hanggang 4 na linggo.

Tawagan kami kung wala kang Internet o kailangan mo ng anumang tulong sa bahaging ito!

Hakbang 2: Kolektahin ang iyong (mga) sample ng dugo.

- Sundin ang mga direksyon sa nakatiklop sa Hakbang sa Patnubay, o tingnan ang mga instrusyon sa video sa CalScope.org upang kolektahin ang (mga) sample ng dugo sa naiturok na daliri.

Hakbang 3: Ipadala sa koreo ang iyong (mga) test kit sa loob ng 2 linggo gamit ang pre-paid USPS mailer bag.

Ano ang susunod? Kunin ang iyong mga resulta sa pagsusuri sa COVID-19 sa koreo ng 6-8 na linggo pagkatapos na matanggap ng lab ang iyong mga sample ng dugo.

Mga katanungan? Mag-email **calscope@cdph.ca.gov** o tumawag sa **1-833-580-1333**.

ITAGO ANG KARD NA ITO PARA SA IYONG MGA REKORD

先读我

按照以下步骤填写调查并采集血样。 **参与研究的孩子需要成人的帮助。**

开始之前，先找到检测盒内的贴纸，然后复制 6 **个字符激活**码用于在线调查：

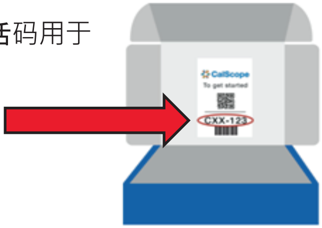

C __ __ __ __ __

第 1 步：访问并完成在网上调查。

- 访问 CalScope.org 或使用智能手机相机扫描检检测剂盒内的快速响应 (QR) 码。单击"我收到试剂盒了 (I GOT A TEST KIT)"。
- 输入激活码和 5 位邮政编码，激活检测试剂盒并填写在线调查。
- 调查结束时，选择希望通过电子邮件还是邮寄方式接收礼品卡。邮寄可能需要长达 4 周的时间。

***如果无法上网或需要这部分的帮助，请打电话！**

第 2 步：采集血样。

- 按照折叠的分步指南中的说明进行操作，或在 CalScope.org 网站上查看视频说明以采集指尖点刺血样。

第 3 步：使用邮资已付的美国邮政 (USPS) 邮袋**在** 2 **周内寄回**检测试剂盒。

下一步? 在实验室收到血样后，COVID-19 抗体检测结果会在 6-8 周寄到。

疑问吗？电邮 **calscope@cdph.ca.gov** 或致电 **1-833-580-1333**。

保留这张卡以作个人记录
