# Supplementary material for: CalScope: methodology and lessons learned for conducting a remote statewide SARS-CoV-2 seroprevalence study in California using an at-home dried blood spot collection kit and online survey
Source: BMC Med Res Methodol. 2024 May 27;24:120. doi: 10.1186/s12874-024-02245-y (PMC11131314; doi:10.1186/s12874-024-02245-y)
Supplement: Supplementary file 1 — Supplementary Material 1. [file 12874_2024_2245_MOESM1_ESM.zip › E. Test Kit Manual.pdf]

CalScope

COVID-19 Antibody Test
Step-by-Step Guide

Test Kit Return Checklist:

- ☐ Online test kit survey(s) were completed at CalScope.org.
  You must complete this step to get your gift card(s).
- ☐ Your age, sex, race, and collection date are written on the blood spot card.
- ☐ Blood spot card is fully dry with at least one full spot.
- ☐ Blood spot card is in the zipper bag and inside the test box.
- ☐ Test box is in the sealed pre-paid USPS return mailer bag.

Stanford MEDICINE   CDPH PublicHealth   ENABLE BIOSCIENCES

1

BEFORE YOU BEGIN: Find the blood spot card in your test box. If both an adult and child will be collecting blood spots, make sure each person uses the correct card.

The adult will use the BLUE card "ADULT | 1".

Write the adult's AGE, SEX, RACE and collection date on the BLUE card.

The child will use the PINK card "CHILD | 2".

Write the child's AGE, SEX, RACE and collection date on the PINK card.

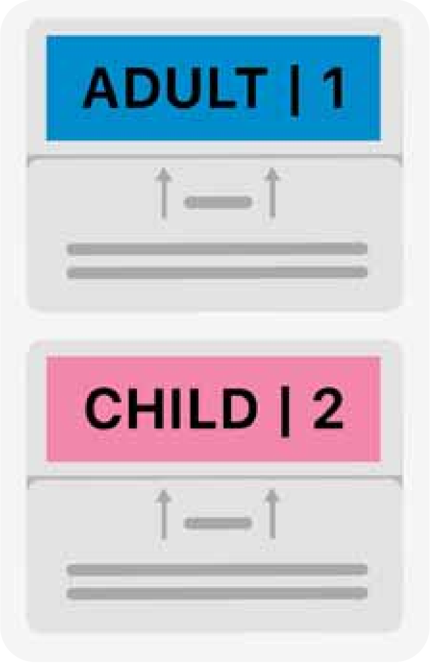

This study is anonymous. All information will be kept secure.

2

Open all packets and lay out the items on a clean surface (e.g. a towel or placemat).

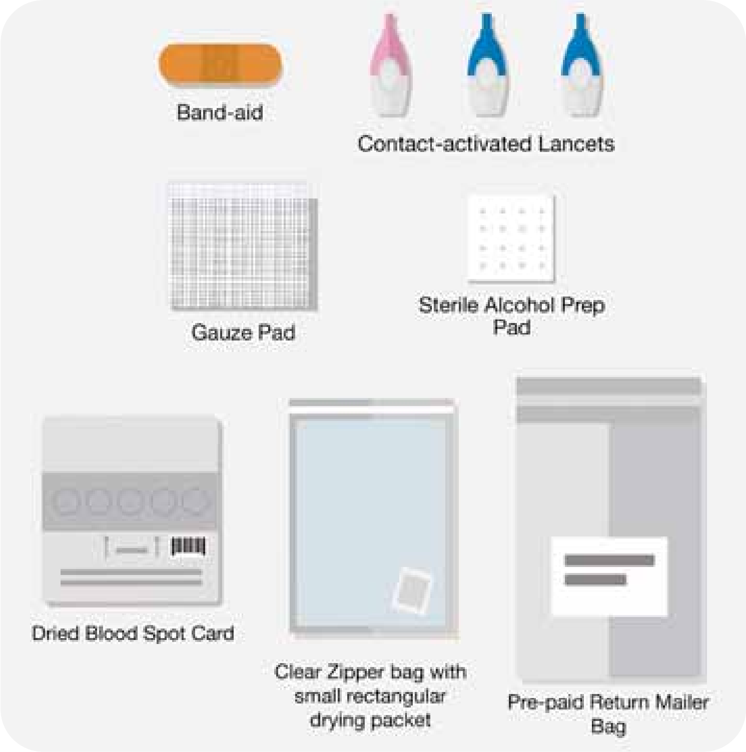

3

Wash your hands in warm water and clap or wave them dry. You should also shake your hands below your waist for about 30 seconds to get your blood pumping!

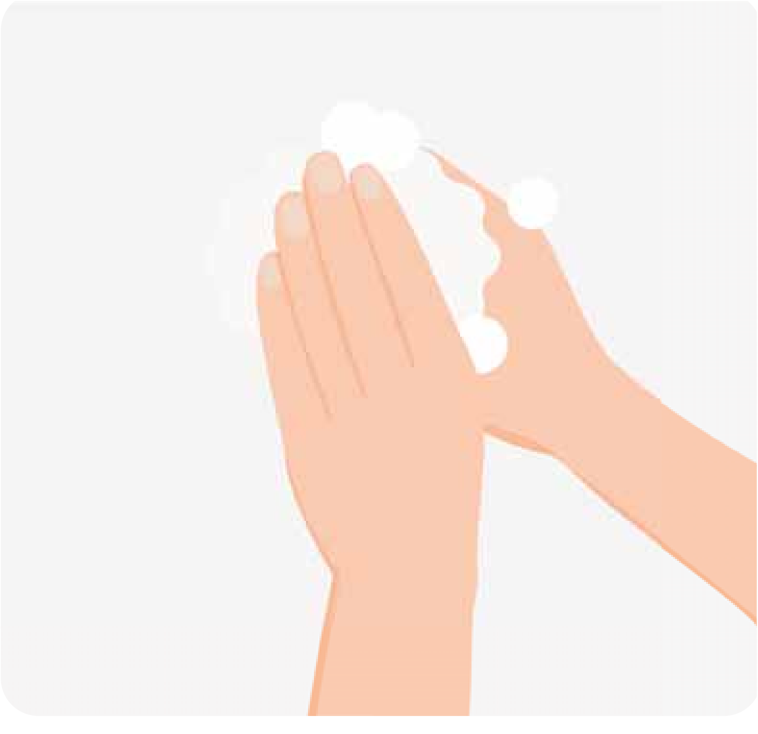

4

Clean the tip of the ring finger (the finger next to your pinky) of your non-writing hand with the alcohol pad.

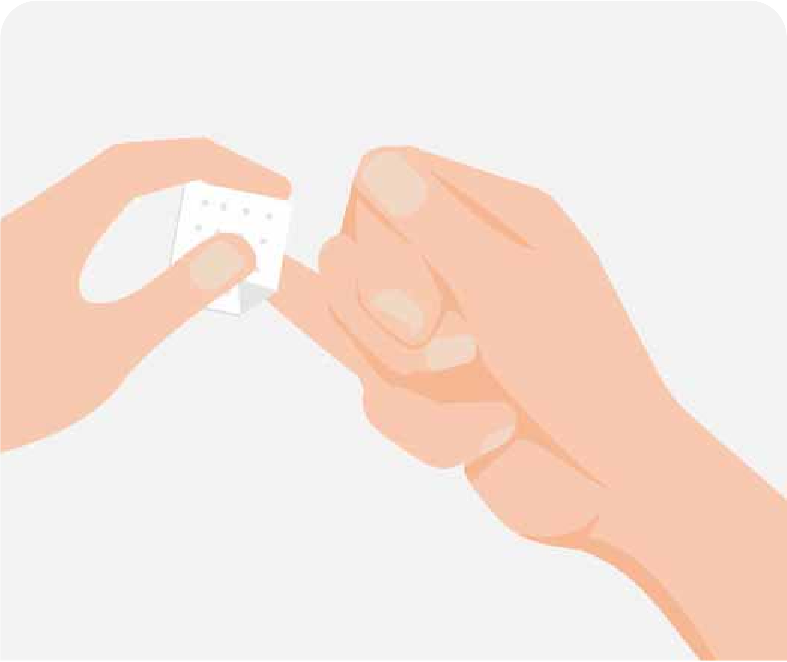

Wait for the finger to dry.
Do not blow on the finger with your mouth.

5

Twist off the small end tip from the lancet.

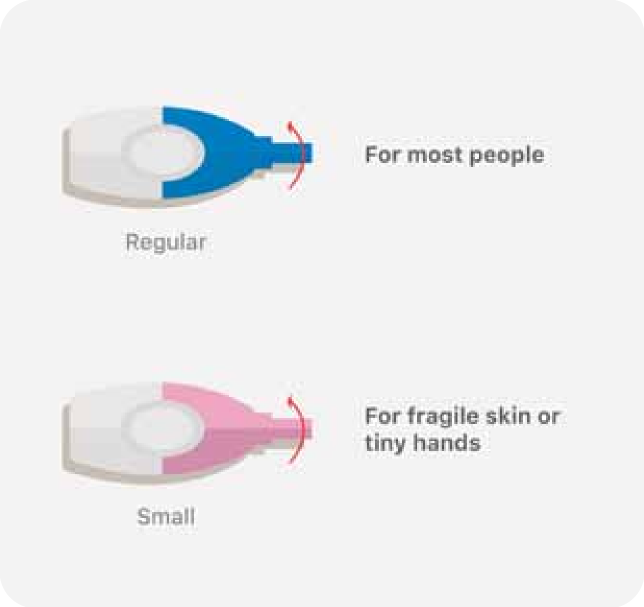

A lancet contains a small needle inside that is used to quickly prick your finger and collect small drops of blood. People with diabetes regularly use lancets to check blood sugar levels at home.

6

Rest your hand on a flat surface like a table.
Place the lancet on the tip of your ring finger, on the side that is closest to your pinky finger.

Press the lancet firmly into your finger until it clicks.

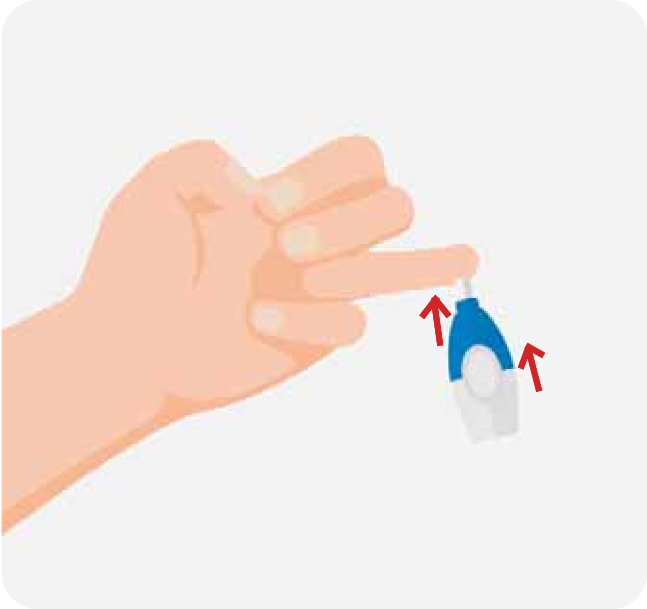

Someone else can help you with this if you find it hard to do.

7

Wipe away the first drop of blood with a gauze pad. Gently squeeze your finger toward the tip to help blood flow.

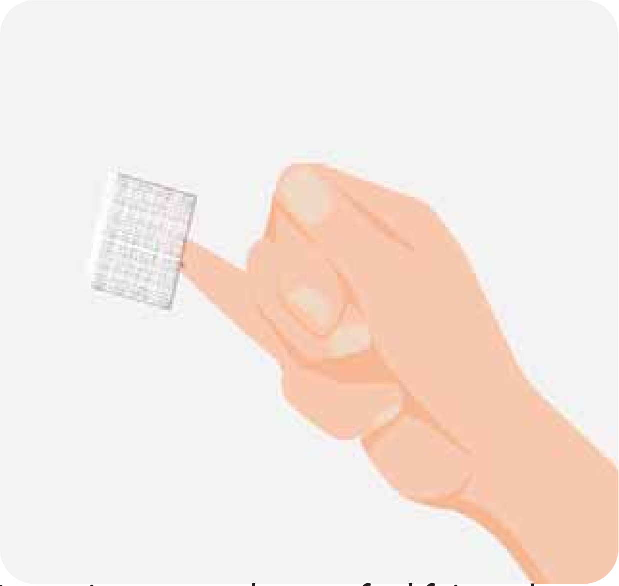

Sometimes people may feel faint when seeing blood. If this happens, stop the test and lie down on the floor until you feel better. Have someone else in your household help you with the blood collection while you are lying down.

8

Without touching the blood spot card, allow drops of blood to fall on each circle until the circle fills. Once you have filled as many circles as you can (at least 1), press the gauze pad onto your finger until bleeding stops.

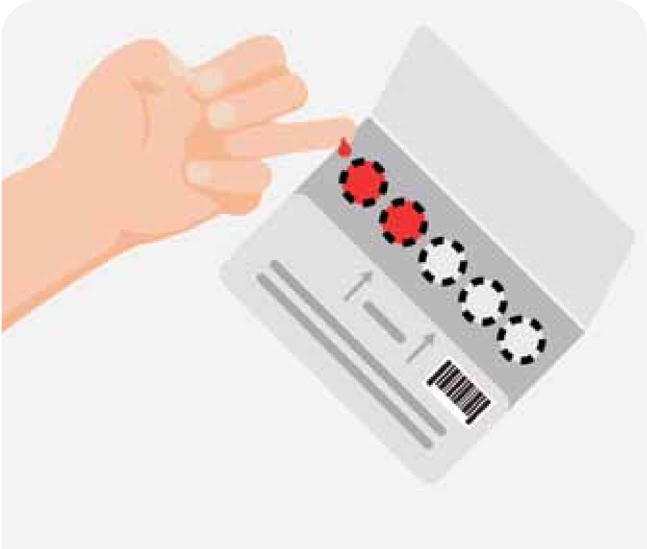

If you did not get enough blood, try using a second lancet on a different finger (like your middle finger) and press it more firmly against the finger.

9

Leave the card open for 4 hours to dry, then tuck in the cover of the card. Place the dried blood spot card and used lancets in the clear zipper bag with the small rectangular drying packet.

Close the zipper bag.

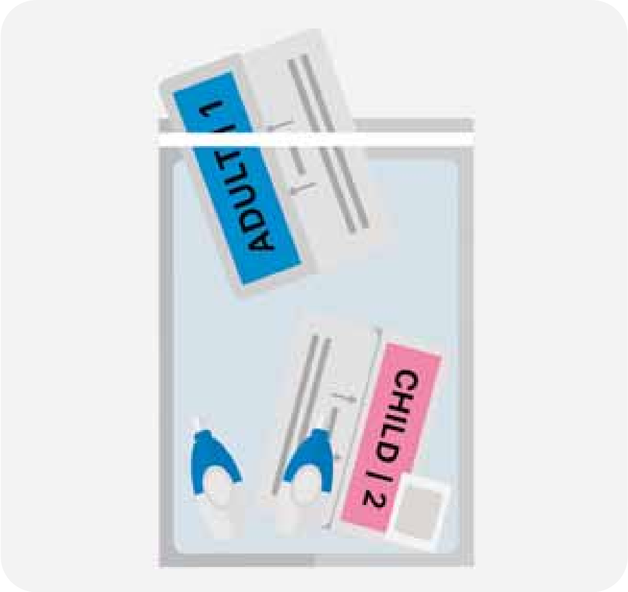

10

Place all unused lancets in the test box. Put the clear zipper bag with the dried blood spot cards and used lancets into the box too. Then, put the whole box into the pre-paid return mailer bag and drop it in a USPS mailbox or at the Post Office.

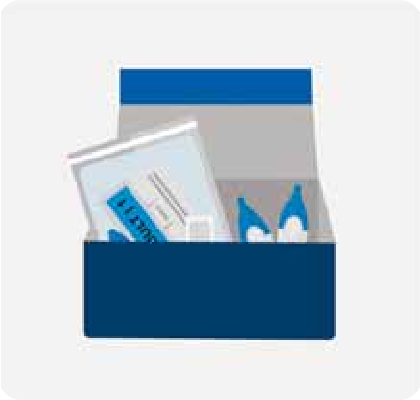

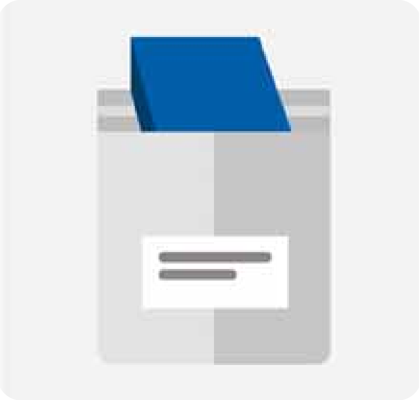

YOU'RE DONE!

RESULTS WILL BE MAILED BACK IN 6-8 WEEKS.

REMINDER

Did you finish the online survey(s) for your blood samples?
You must complete the survey in order to get the gift card(s).
The registration form you filled out to order your test kit is NOT the study survey.

Activate your test kit and finish your survey by going online to www.CalScope.org/#testkit.
In the I GOT A TEST KIT portal, enter in your 6-character activation code printed on the inside of your test box.

I got a test kit

Please enter the activation and zip code below.

Enter Activation Code

Enter Your Zip Code

Confirm

Please check the box below to proceed

I'm not a robot

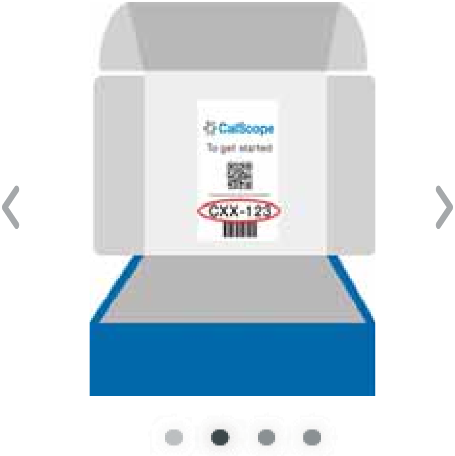

Call us at 1-833-580-1333 if you don't have Internet or need help!
