# Supplementary material for: CalScope: methodology and lessons learned for conducting a remote statewide SARS-CoV-2 seroprevalence study in California using an at-home dried blood spot collection kit and online survey
Source: BMC Med Res Methodol. 2024 May 27;24:120. doi: 10.1186/s12874-024-02245-y (PMC11131314; doi:10.1186/s12874-024-02245-y)
Supplement: Supplementary file 1 — Supplementary Material 1. [file 12874_2024_2245_MOESM1_ESM.zip › F. Call center protocol.pdf]

CalScope

California Department of PublicHealth

Protocols for Call Center and Participant Communications

CONTENTS

CALSCOPE CONTACT & ACCOUNT INFORMATION
OVERVIEW
RESPONSIBILITIES FOR CALL CENTER STAFF
STUDY SUMMARY
PARTICIPANT FLOW DIAGRAMS
PHONE COMMUNICATIONS
E-MAIL COMMUNICATIONS
DOCUMENTATION
REGISTRATION & SURVEY(S)
ACCESS & KIT ACTIVATION CODES
FAQS
REFERENCE MATERIALS AND LINKS

CALSCOPE CONTACT & ACCOUNT INFORMATION

**PUBLIC FACING**	
Website	www.Calscope.org
E-mail address	calscope@cdph.ca.gov
Toll-free number (outgoing only)	
Twilio Interactive Voice Response (IVR) phone number	
**INTERNAL CONTACT/ACCOUNT INFORMATION**	
Internal phone number associated with toll-free number	
Internal voicemail access phone number	
Phone number to access language line services	TBD

OVERVIEW

RESPONSIBILITIES FOR CALL CENTER STAFF

1. RESPOND TO SUPPORT REQUESTS RECEIVED THROUGH EMAIL, PHONE, AND WEBSITE
2. ASSIST PROSPECTIVE PARTICIPANTS OR PARTICIPANTS WITH:
    - Study registration
    - Survey completion
    - Specimen collection process
    - Participant follow-up and troubleshooting
        i. Receiving results
        ii. Specimen resubmission
        iii. Gift card issues
3. DOCUMENTATION OF FOLLOW-UP ACTIVITIES THROUGH REDCAP FOLLOW-UP FORM
4. REFER PROSPECTIVE PARTICIPANTS OR PARTICIPANTS TO OTHER RESOURCES AS NECESSARY

STUDY SUMMARY

Random households within 7 California counties (Alameda, EL Dorado, Kern, Los Angeles, Monterey, San Diego, and Shasta) will receive an invitation letter or postcard asking up to 2 members (1 adult and 1 child) to participate in the study by answering surveys and testing for COVID-19 antibodies using an at-home test kit. Children must be between 1 to 17 years old. Each survey and blood test will be compensated with a $20 gift card, for a maximum total of $80 per household if both an adult and child fully participate in the study.

If multiple adults and children are willing to participate in the study, the person with the next upcoming birthday should join the study.

To sign up for the study and to order testing kits (up to 1 adult and/or 1 child), an adult from the household will use the unique 8-digit access code provided in the invitation letter or postcard (circled in red) and their zip code to register online at CalScope.org through the "I got an invitation" portal.

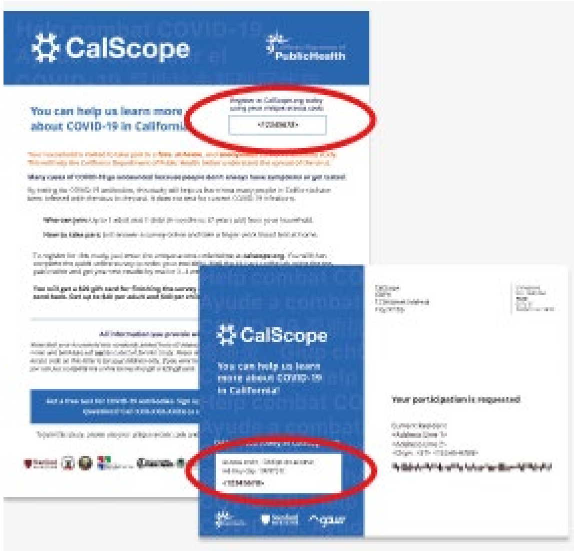

Figure 1. Invitation letter and post card with highlighted access codes

The registration survey will ask participants to:

- Count the number of adults, children (6 months to 17 years old), and infants (less than 6 months old).
- Order kits for up to 1 adult and/or 1 child in the household.
- Provide contact information for study updates and communications.

Households are also allowed to answer just the survey if an adult and/or child does not want to participate in the blood collection phase of the study. However, if no kit is ordered at all for a household with children, the household will only be given the option of answering the adult survey only- not both the adult and child survey. Gift cards will be disbursed to participants at the end of the survey based on the disbursement option chosen by the participant at the end- either by email, text, or mail. Electronic gift cards will be sent as Tango gift card links, which is redeemable under a variety of brands. Physical cards are only available to be redeemed as Amazon, Target, or Walmart gift cards.

Testing kits will be shipped to the participant's home address within 1-2 weeks.

In summary, households may only complete the survey in the following situations:

- Without children
    - The adult may answer just the survey if no adult test kit is ordered for the household.
- With children
    - The adult may answer just the survey if no adult and child test kit is ordered for the household.
    - The adult may answer just the survey if only a child test kit is ordered for the household.
    - The child may answer just the survey if only an adult test kit is ordered for the household. If appropriate, the adult may answer the questions for the child.

To activate the COVID-19 testing kit, the household participant will use the 6-character activation code (beginning with a "C") provided on the underside of the test box lid and their zip code to register the kit online at Calscope.org/#gotkit through the "I got a test kit portal". They may also be taken to the website directly by scanning the QR code provided on the label.

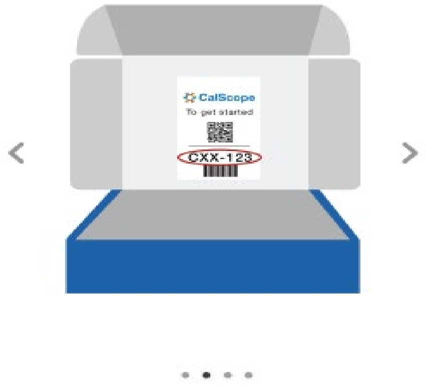

Figure 2. Test kit box with label showing highlighted activation code underneath the lid

Once registered, participants will be taken to the household landing page, which will list the surveys (adult and/or child) to be completed as well as the blood collection video that participants can watch.

The household landing page may contain the following items:

- Adult survey
    - Includes questions on household and adult participant's demographics, income, occupation, medical and COVID-19 disease history, and COVID-19 risk factors.

- Child survey
    - Includes questions on child participant's demographics, medical and COVID-19 disease history, and attendance in school and other social events/activities.
    - May be completed by an adult if appropriate.

- Blood collection instructional video (the link to view the video only appears when at least 1 survey is completed).

After completion of all household surveys as well as blood collections, the completed test kits will be mailed back to the laboratory using the enclosed pre-paid mailer bag.

Gift cards will be disbursed to participants when the lab the completed test kits, based on the method that was chosen by participants at the completion of the survey(s).

PARTICIPANT FLOW DIAGRAMS

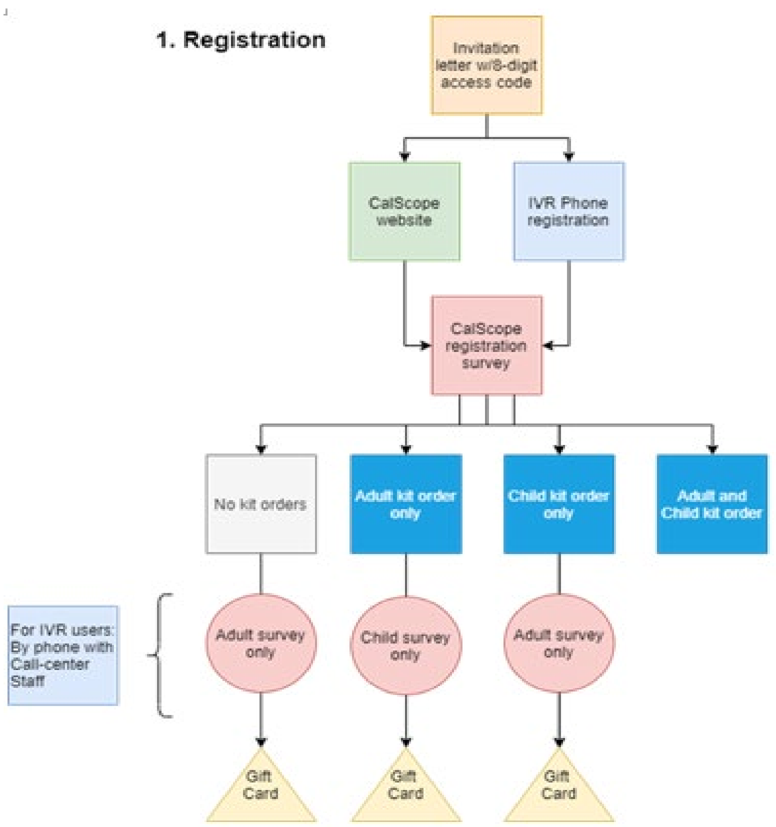

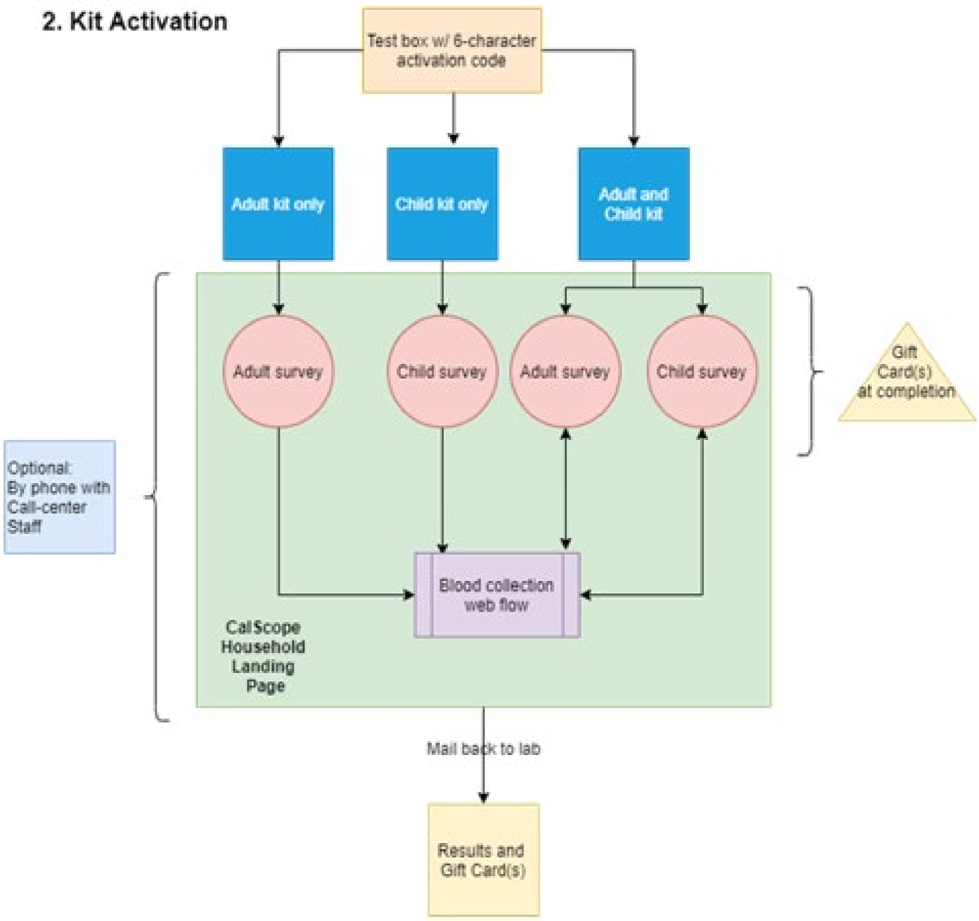

PHONE COMMUNICATIONS

MAKING OUTGOING CALLS TO PARTICIPANTS

All outgoing calls should be made using the shared AT&T virtual office line so that calls are masked and will appear to originate from "CDPH CalScope" instead of caller's personal cell/desk phone. Use of the virtual office will require connection to a staff member's mobile or landline but calls to participants will appear to originate from "CDPH CalScope".

1. VIA AT&T VIRTUAL OFFICE

a. Log in to AT&T Premier Care website: www.wireless.att.com/premiercare [Use Log in info on Page 1]
b. Note: when logging in, you may be asked to "Verify your identity" by sending and authentication account email address. Select Email (calscope@cdph.ca.gov). The one-time passcode will be sent to calscope@cdph.ca.gov inbox
c. See CDPH CalScope User Portal Instructions for detailed instructions.

** **IMPORTANT: Before each person begins a new "shift" making outgoing calls to participants, they must enable connection to their own mobile/landline in the Virtual Office and make sure that other staff members' lines are disabled.**

RETRIEVING VOICEMAIL MESSAGES TO THE OUTGOING ONLY NUMBER

Although participants will not be directed to contact the study team through the CalScope toll-free number, some follow-up participants may use the Caller ID number to try and contact the team. In anticipation of these cases, the following should be observed:

2. VIA CALSCOPE@CDPH INBOX (PREFERRED)

All phone calls to the CalScope toll-free number will be sent straight to voicemail and will be sent as .wav files to calscope@cdph.ca.gov. Subject line will read: "Voicemail from [CALLER NUMBER] to XXX"

3. VIA PHONE

a. Call the VM Access number

b. Enter the 10-digit phone number

c. Enter the VM PIN
Reference additional voicemail retrieval instructions and voicemail navigation map as needed

4. VIA AT&T PREMIER CARE WEBSITE

See CDPH CalScope User Portal Instructions for details

****Do not delete any voicemails from CalScope@cdph inbox or AT&T voicemail message system**

USING LANGUAGE LINE SERVICES

For interpreters, CalScope team members may utilize the language line services available through the CDPH testing triage line at XXX. For information on the detailed procedures and processes, refer to the Language line protocol.

Figure 3. Enable/Disable staff member lines as needed for use during shifts.

# E-MAIL COMMUNICATIONS

E-MAIL COMMUNICATIONS TO AND FROM STUDY INBOX (CALSCOPE@CDPH.CA.GOV)

All e-mail communications between participants and CalScope team should be made using the CalScope@cdph.ca.gov communal inbox. When sending e-mails from CalScope@cdph, verify they are coming from CalScope@cdph and not your individual email address:

- In Outlook: Options tab → Select "From"
- Select calscope@cdph.ca.gov. If the option does not appear, select "Other email address" → enter "calscope@cdph.ca.gov" → ok

SUBJECT LINE

Make sure to reference the REDCap Support Module Record ID when communicating with participants by email to make sure that all communications are being logged correctly and previous conversations can be found in the inbox appropriately.

- Format: CalScope Study – [Support ID: XXX] [brief summary of issue]
- Example: CalScope Study – [Support ID: 001] Trouble signing up online

EMAIL SIGNATURE

California Department of Public Health
COVID-19 Response
CalScope Team
calscope@cdph.ca.gov
Note: This e-mail is only monitored from M-F 9AM-5PM

DOCUMENTATION

The Support Module project on REDCap will be used to consolidate all support requests and will be used to track all follow-up communications with participants by phone or email.

NEW INQUIRIES

Participants able to access the online website will have the option of submitting questions and comments through the "Contact Us" link on the calscope.org website. This link will take participants directly to the REDCap Support Module, creating a new record in the project.

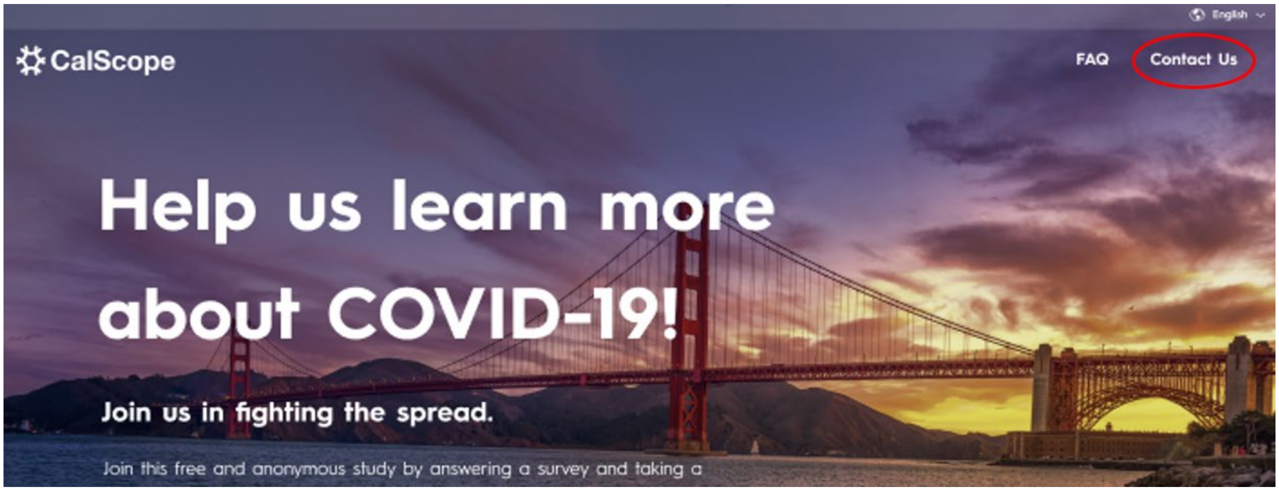

Figure 4. Contact Us CTA on main page of CalScope.org

Participants will be able to choose what type of support is needed and provide additional information such as access/activation codes, home addresses, and contact information that may allow study staff to validate if the user is a participant from an invited household, or a member of the general public that has not received an invitation letter/postcard.

Support Form

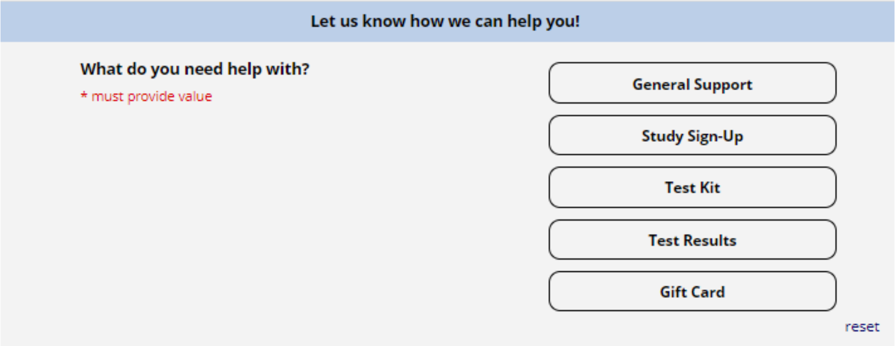
Let us know how we can help you!

What do you need help with?
* must provide value

General Support
Study Sign-Up
Test Kit
Test Results
Gift Card

reset

Figure 5. Support Module survey on REDCap accessible through Contact Us CTA on main page of CalScope.org

SUPPORT FOLLOW-UP

The **"Follow-up #1"** excel on the shared CalScope Support Follow-Up folder will list new support forms that have not yet been addressed by study staff. The results are ordered first by language and date/time of submission from oldest to newest submission.

	A	B	C	D	E	F	
1	Record ID	Date and Time of !	Which lan	What do you nee	What is th	What is th	W
2	20	2/8/2021 10:39	English	General Support			
3	21	2/8/2021 10:41	English	Study Sign-Up			
4	22	2/8/2021 13:52	English				
5	26	2/16/2021 15:16					
6	34	3/22/2021 8:53	English	General Support			
7	35	3/24/2021 15:24	English	General Support			
8	38	4/6/2021 10:43	Filipino	Test Kit	468	546	
9	43	4/8/2021 14:12	English	Gift Card			

The **"Follow-up #2"** file contains support requests that have already been followed-up once but did not have the issue or questions resolved and needs a second additional follow-up. The records in this excel file is ordered first by the requested date of follow-up #2, name of staff member for follow-up #1, and language.

The **"Follow-up #3"** file contains support requests that have already been followed-up twice but did not have the issue or questions resolved and needs a third additional follow-up. The records in this excel file is ordered first by the requested date of follow-up #3, name of staff member for follow-up #2, and language.

The **"Ongoing Follow-Up"** file contains support requests that have already been followed-up at least 3 times but did not have the issue or questions resolved and needs an additional follow-up. The records in this excel file is ordered first by the date of previous follow-up #3 and language.

FOR EACH FOLLOW-UP ACTIVITY (by phone or email), make sure to document the following in the **REDCap Follow-Up** form available at https://redcap.link/XXX.

For each form, the following information will need to be entered:

1. **RECORD ID** of the support request that is being followed-up
2. **Type of Follow-Up**: Follow-Up #1, Follow-Up #2, Follow-Up #3, Ongoing Follow-Up
3. **Date and Time** of Follow-Up (D-M-Y H:M)
4. **Method of Communication**: Email or Phone
   a. If by phone:
      i. Did you use the interpreters through language line?
      ii. Did the participant answer the call?
5. **Staff Member** making follow-up contact
6. **Notes**
7. **Issue resolved?** Complete or Need to Follow-Up
   a. If need to follow-up:
      i. By what date and time?
      ii. How do they want to be contacted?

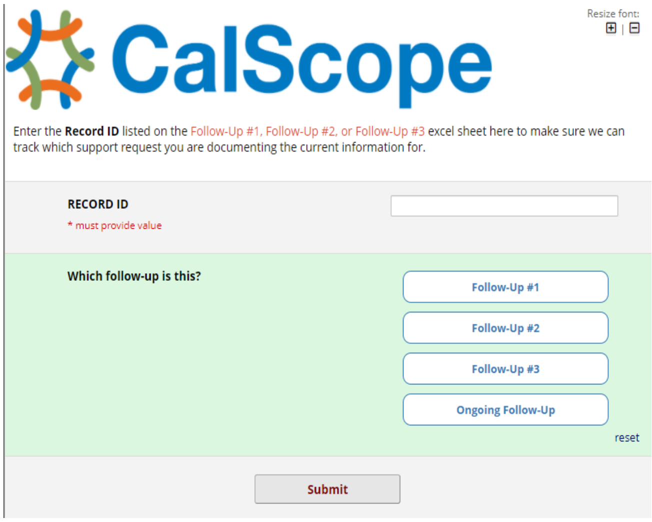

In consideration of other callers who may also be working off of the same spreadsheet- make sure to record your name and date/time of follow-up on the appropriate excel sheet record so that other callers do not try to contact the same participant again.

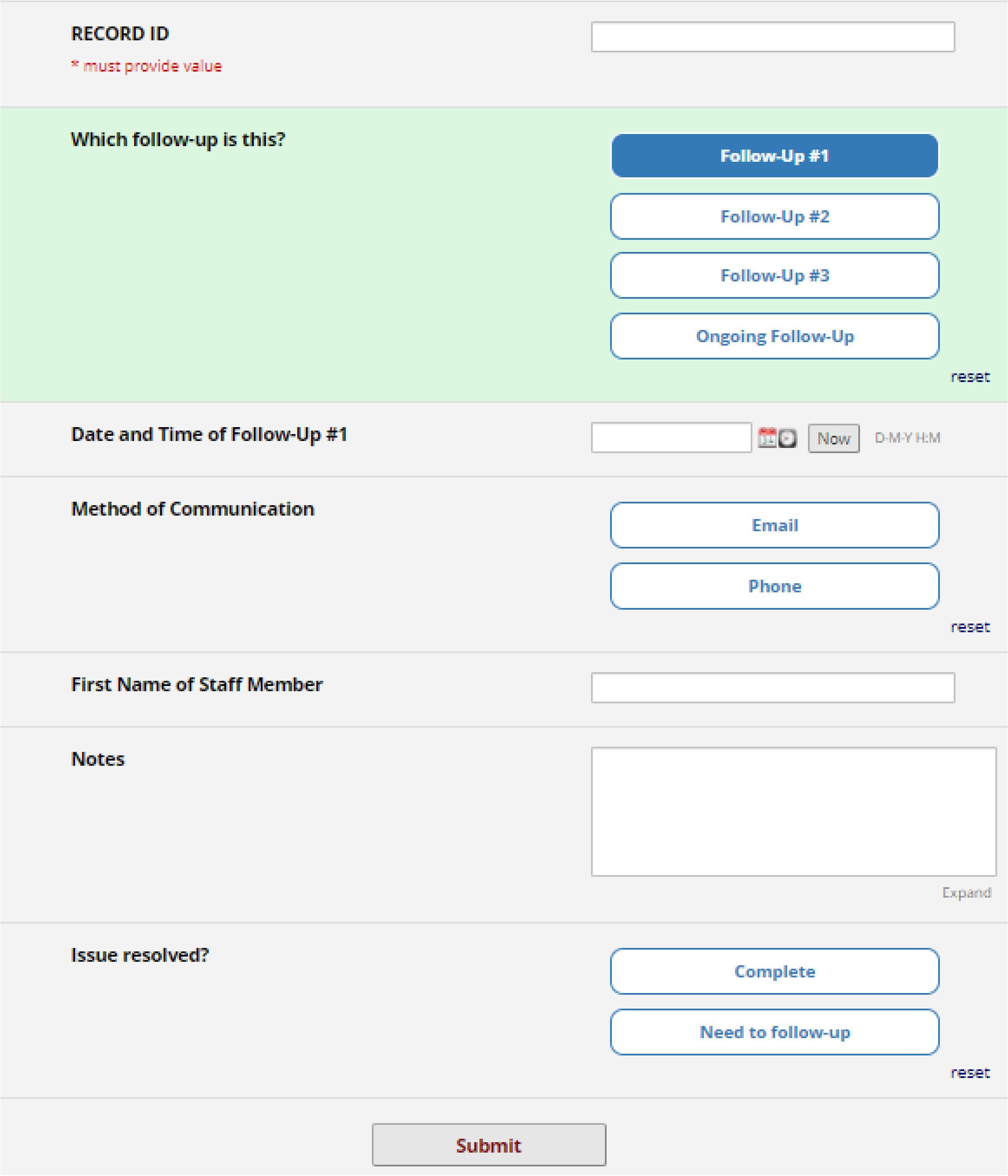
RECORD ID
* must provide value

Which follow-up is this?
Follow-Up #1
Follow-Up #2
Follow-Up #3
Ongoing Follow-Up
reset

Date and Time of Follow-Up #1
Now D-M-Y H:M

Method of Communication
Email
Phone
reset

First Name of Staff Member

Notes
Expand

Issue resolved?
Complete
Need to follow-up
reset

Submit

Figure 6. Screen shot of REDCap Follow-Up form

REGISTRATION AND SURVEYS

The REDCap registration and kit activation surveys can be accessed through the CalScope.org website.

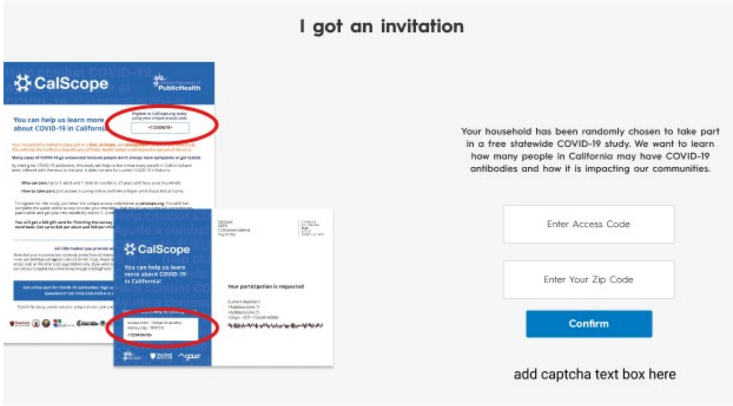

Figure 7. I got an invitation section used for study registration

When participants receive their invitation letter or postcard, they can register for the study directly by phone keypad or verbally over the phone with study staff, if they cannot access the online website.

To register directly by phone, participants will call into the study 1-833 number and select option #1 to sign up for the study. Answers will then be submitted using the phone's keypad. To register verbally with the help of study staff, participants may call into the study 1-833 number and leave a voice message (options 2 or 9) for staff to return the call and help.

When assisting participants verbally over the phone for study sign-up, staff will need to complete the online registration for the participant by entering in the access and zip codes for the participant online. Staff will then complete the registration survey by repeating the questions and entering in the participant's answers. If the participant elects to order no kits for eligible adults and/or children in the household and instead chooses to only participate in the survey, the staff member should verbally complete the corresponding survey over the phone.

Participants directly completing registration by phone keypad may also need to be followed-up by study staff for verbal completion of the optional adult/child survey if they elect to complete one without ordering the corresponding test kit.

When participants receive their ordered test kit(s) by mail, they can only activate the kit by leaving a message on the study 1-833 number (options 4 or 9) or sending an email to calscope@cdph.ca.gov asking for assistance, if they cannot access the online website.

When assisting participants verbally over the phone for kit activation, staff will need to complete the kit activation process by entering in the activation and zip codes for the participant online through calscope.org/#gotkit.

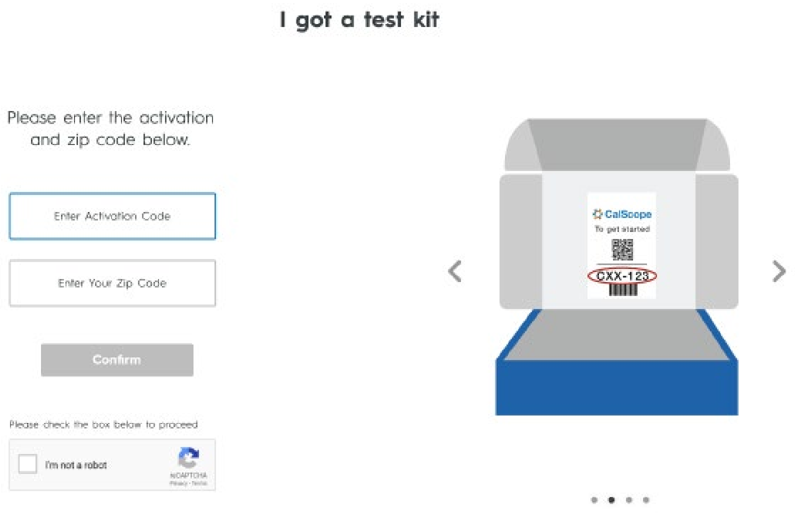

Figure 8. I got a test kit section used for kit activation. Directly accessible by scanning the QR code on the test box label or typing in CalScope.org/#gotkit in the Internet browser.

The landing page will look differently based on the types of test kits that were ordered by the household:

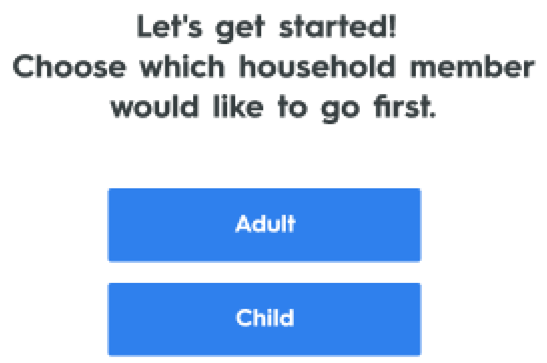

Figure 12a. Landing page for households that order both an adult and child test kit

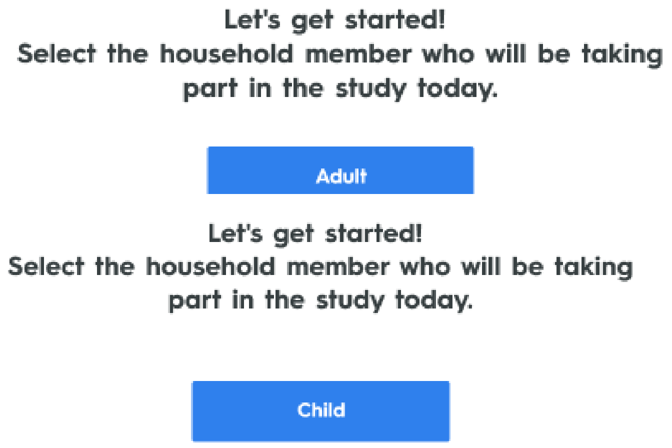

Figure 9b. Landing page for households that order only an adult or child test kit

Staff will then complete the adult and/or child survey by repeating the questions and entering in the participant's answers.

When at least one survey has been completed, the link to view the video instructions for the dried blood spot collection will appear. For households with 2 surveys, the link to the other survey will also appear alongside the link to the video instructions.

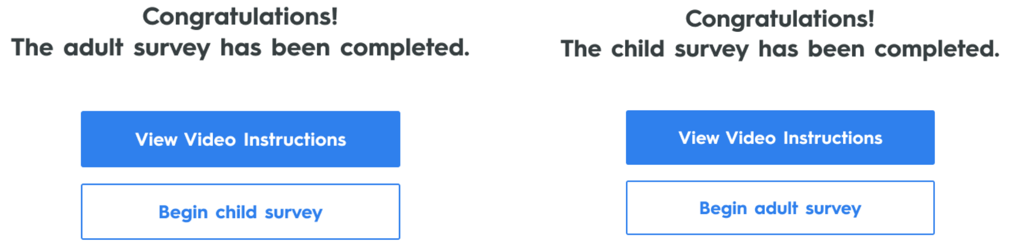

Figure 10a. Link to view the video instructions will appear as soon as at least 1 survey is complete.

Participants may choose to complete the other survey or complete the blood collection for COVID-19 antibody testing. Since the video instructions are similar to the written instructions, staff may read through the video instructions and have the participants follow along with the enclosed instruction manual. The video instructions also have no audio and are stepwise instruction panels that need to be clicked through, so staff should be able to read through the panels without any issues.

There are no limits to how many times the video instructions can be viewed so participants will be able to access it as much as they want.

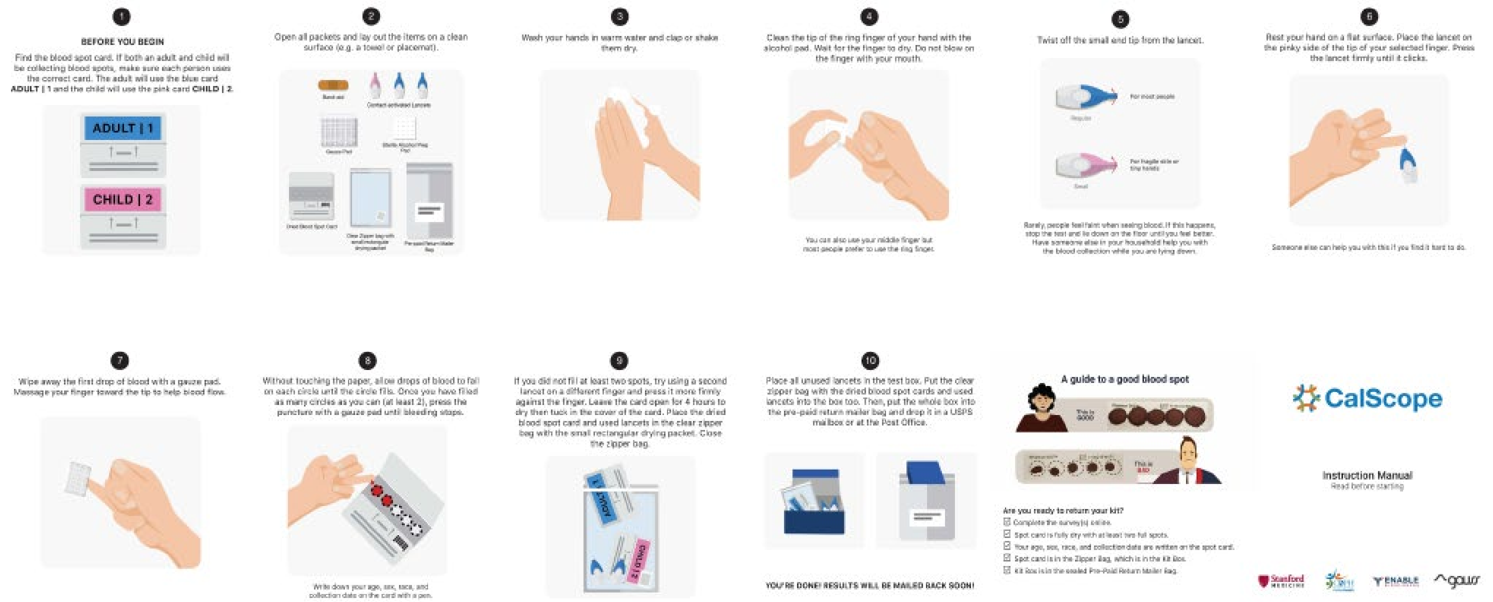

Figure 11. The written instructions provided in the test kit's instruction manual

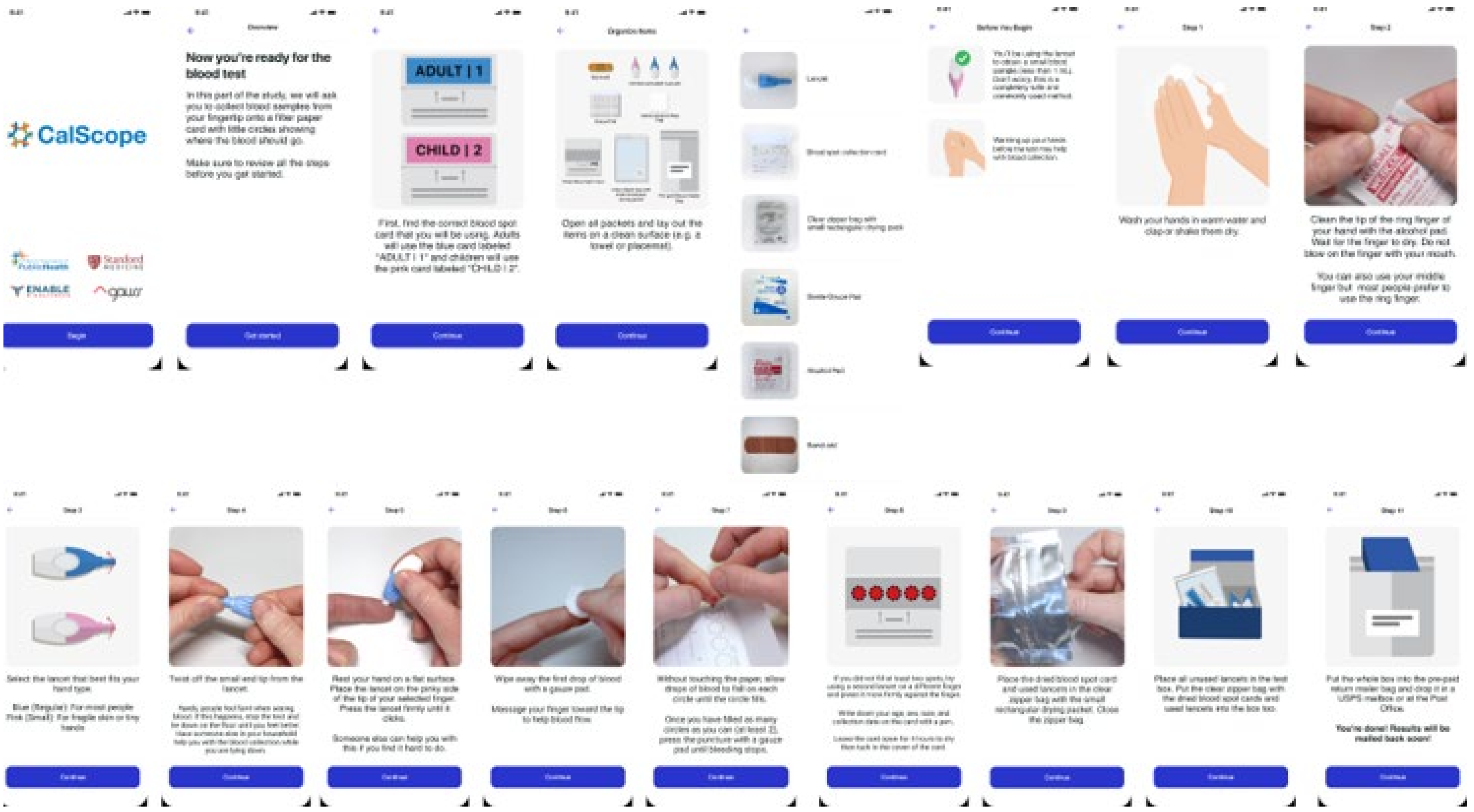

Figure 12. Video instructions provided through household landing page

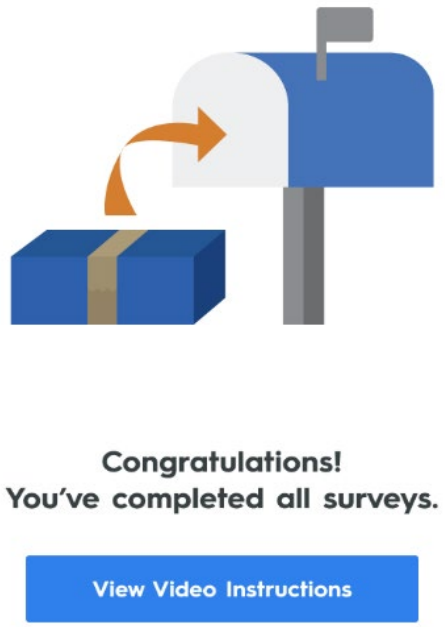

Once all the surveys are completed, participants can be walked through the blood collection process again as needed and instructed to return all completed dried blood spots together in the same test kit box by USPS mail using the enclosed pre-paid mailer bag.

Gift cards will be disbursed to participants when the lab receives the completed test kits and will be delivered through the same method that was chosen by the participant for the survey gift card. All test results will be returned by mail within 3-4 weeks.

ACCESS AND ACTIVATION CODES

Both files are located in the Codes folder under Support Follow-Up and can be viewed as needed when following-up with support requests from participants. The files are updated daily.

ACCESS CODES

The "Access_Codes" excel file lists all of the addresses that have been sent an invitation letter with printed access code. It also contains the following variables:

- **[Participant Used By]**- RECORD ID created in the main database after participant successfully completed registration. If this field is blank, it means that the access code for the address has not been used for registration through the website or IVR system yet.
- **[Participant Used By Date]-** Date that the access code was used for registration. If this field is blank, it means that the access code for the address has not been used for registration yet.
- **[Usage Attempts]**- The number of times the access code was entered in the website for registration.

ACTIVATION CODES

The "Activation_Codes" excel file lists all of the addresses that have ordered a test kit and has been assigned an activation code. It also contains the following variables:

- **[1 Adult Kit]-** Did the household order 1 adult test kit? If this field is blank, no adult test kit was ordered.
- **[1 Child Kit]-** Did the household order 1 child test kit? If this field is blank, no child test kit was ordered.
- **[Kit Shipped Date]-** Date the test box with 1 or 2 test kits were shipped out to the address. If this field is blank, no test box has been shipped out to the address yet.
- **[Test Box Received by Enable]-** Date the test box was received by the lab for testing. If this field is blank, no test box was received by the lab yet.
- **[Adult DBS Received at Lab Date]-** Date the adult DBS card was received by the lab for testing.
- **[Child DBS Received at Lab Date]-** Date the child DBS card was received by the lab for testing.

If an address cannot be found on this excel sheet, it means that the order is still processing, and no test kits have been shipped out to the address yet.

CALSCOPE INTERNAL FAQS

Public-facing FAQs are available on the CalScope website at www.calscope.org in all 4 study languages.

Add additional detailed FAQs internal to team as they arise

REFERENCE MATERIALS AND LINKS

- Call Center FAQs
- Study flow diagrams
- CDPH CalScope User Portal Instructions
- Call center coverage calendar/schedule
- Telephone Call Scripts
- Language line protocol
- REDCap Follow-Up Form_Codebook and Instructions
