# Supplementary material for: CalScope: methodology and lessons learned for conducting a remote statewide SARS-CoV-2 seroprevalence study in California using an at-home dried blood spot collection kit and online survey
Source: BMC Med Res Methodol. 2024 May 27;24:120. doi: 10.1186/s12874-024-02245-y (PMC11131314; doi:10.1186/s12874-024-02245-y)
Supplement: Supplementary file 1 — Supplementary Material 1. [file 12874_2024_2245_MOESM1_ESM.zip › G. Communications Toolkit.pdf]

Dear Local Health/Community Partner:

As a program committed to promoting health in your area, you have the expertise and partnerships to reach members in your community about important health issues. Our team at the California Department of Public Health (CDPH) is reaching out to ask for your help in building awareness about CDPH's COVID-19 study called **CalScope**.

COVID-19 continues to be a health risk to Californians. **CalScope** is a population-based serosurvey designed to help CDPH learn how many Californians have antibodies (from a past infection or vaccination) to SARS-CoV-2, the virus that causes COVID-19. This study will also help CDPH learn how population immunity to COVID-19 is changing over time throughout our state. CalScope consists of three phases of data collection: Wave 1 conducted April – August 2021, Wave 2 conducted October 2021 – January 2022, and Wave 3 planned for April – July 2022.

Your county is 1 of 7 counties selected to participate in the CalScope study to represent California. Thousands of households in your county are being randomly selected and invited to take a **free**, anonymous, at-home COVID-19 antibody test and survey. Each selected household will receive an invitation in the mail from CDPH with a unique access code for up to one adult and/or one child (ages 1-17) to sign up online or over the phone. Each participant will receive a $20 gift card for completing the survey and a $20 gift card for returning the blood test sample.

Only those randomly selected households who receive an invitation in the mail are eligible to participate, but **our goal—and ask of you—is to encourage as many invited households as possible to participate.** As a trusted member of your community, you can help others learn more about CalScope and encourage them to participate for the benefit of your county and California as a whole.

To provide further details about the study and assist your efforts in engaging participants, we have a toolkit available that includes:

- **CalScope Fact Sheet for Local Partners** providing an overview of the study
- **CalScope FAQ** for agencies to use to respond to questions from community members
- **CalScope Flyer** with details on study participation
- **CalScope Infographic** with steps for participation
- **Communications Plan** to assist your outreach
- **Social Media Calendar** complete with graphics and messaging and suggested timeline for sharing on social media
- **Customizable Digital Ad Template**

Additional information is also available on our study website: www.CalScope.org. Thank you for collaborating with CDPH to learn more about COVID-19 in California!

Sincerely,

CalScope Study Team
California Department of Public Health
Email: CalScope@cdph.ca.gov
Phone: 1-833-580-1333

CalScope – California's COVID-19 Antibody Study

Fact Sheet for Local Partners

What is CalScope?

CalScope is a population-based serosurvey that will help the California Department of Public Health (CDPH) learn how many Californians have antibodies to SARS-CoV-2, the virus that causes COVID-19. This study will also help CDPH learn how population immunity to the COVID-19 virus may change over time. The overall goal of this study is to understand how many Californians have been infected with SARS-CoV-2 or received the COVID-19 vaccine and how this varies across population subgroups (i.e., by region, age, race, and ethnicity, etc.).

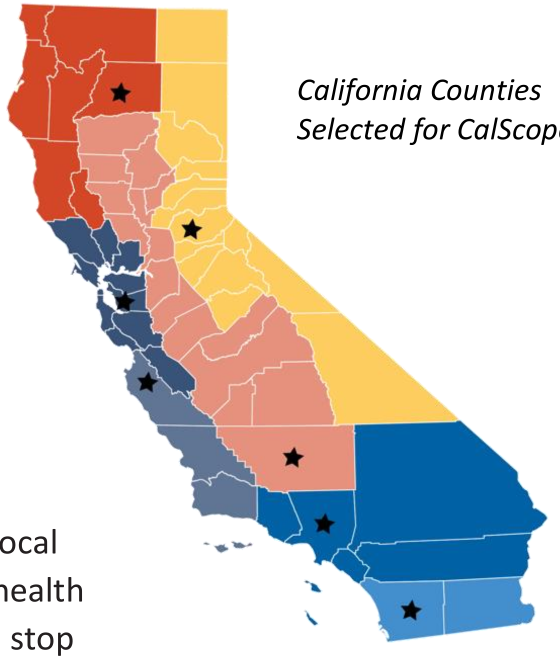

California Counties Selected for CalScope

This study is intended to complement the current efforts of local health programs. Information from this study can help local health programs in their current efforts to promote vaccination and stop the spread of COVID-19 in their communities.

Why is this study important?

Data from antibody studies like CalScope complement state data on COVID-19 to help public health leaders understand how the virus has spread throughout the state and which communities remain at-risk for COVID-19 in California. Understanding how COVID-19 antibodies may differ across groups can also help us identify which populations could benefit from additional resources. Read more about California's commitment to health equity.

Who is involved?

To lead the CalScope study in California, CDPH has partnered with Stanford University School of Medicine and seven local health departments in Alameda, El Dorado, Kern, Los Angeles, Monterey, San Diego, and Shasta counties. Households in these counties are being randomly selected and invited to participate in CalScope.

For more information, email CalScope@cdph.ca.gov.

How does CalScope work?

Up to one adult and/or one child from each household will be invited to self-collect a blood sample at home and complete an anonymous survey about household and individual demographics, behaviors, and impacts of the COVID-19 pandemic. Participants are eligible to receive a $20 gift card for each completed blood sample test and a $20 gift card for each completed survey.

Participation in this study is free and confidential. This study will not ask about citizenship status. Individual information cannot be shared with any other governmental program. Participants will not be asked to provide personal information, such as name or date of birth, and any information collected will be used for the purposes of this study only. If participants in your communities have questions about taking part in CalScope, you can share the CalScope FAQs with them to help answer common questions.

How will this study help my community?

CalScope aims to meet people where they are: at home. This study collects anonymous information from participants even if they cannot access a healthcare facility. The study collects information about infections that would otherwise be missed.

We understand that representation matters. Higher enrollment across different groups in your community will help us gather more accurate data that represent the diverse population of your county.

Results from this study can help community organizations like yours.

- Estimate how many people in the community have COVID-19 antibodies (from vaccination or from a previous infection).
- Identify areas or populations with high amounts of undiagnosed SARS-CoV-2 infection.
- Identify subgroups that might need additional resources.

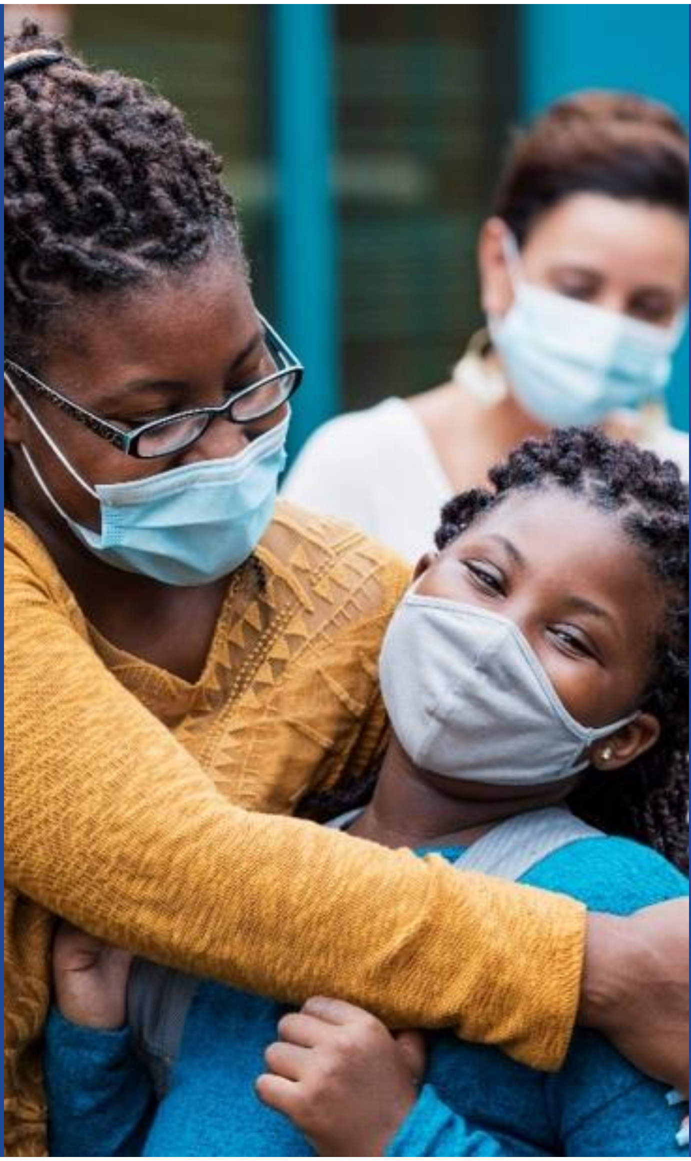

© 2022, California Department of Public Health

CalScope – California's COVID-19 Antibody Study

Frequently Asked Questions – For Public Distribution

What is the goal of the CalScope study?

The goal of this study is to learn how many people in California have antibodies to the virus that causes COVID-19 and how this number may change over time. Information collected from this study will help the California Department of Public Health (CDPH) and local public health departments understand how many Californians have been infected or received the COVID-19 vaccine and how this information varies across different groups in California (e.g., groups by region, age, race and ethnicity, etc.).

Why does CDPH want to know how many people have antibodies to COVID-19?

This information may help identify groups at high risk for infection and help estimate the future impact of COVID-19 in California. Information and data from this study will only be used by the CalScope study team, and does not contain personal information that can identify you.

Why should I participate?

Your participation helps to ensure a large and diverse group of Californians is included in this study so we can understand the impact of COVID-19 across different communities. Your household was randomly selected from millions of other addresses to represent your county. Your participation will help local health programs get a better idea of how a population's immunity may differ across groups including children, working adults, and older adults with underlying medical conditions.

Why can't anyone join this study?

Thousands of households were randomly selected to help ensure information is gathered from a large and diverse group of people—reflecting the diversity of California. This randomized participation will help to understand how population immunity to COVID-19 is changing throughout regions in California and how this impacts different communities.

Is participation in this study confidential?

YES. The survey will not ask for your personal information or citizenship status. All survey and blood test results are collected anonymously (meaning we do not ask for your name) to protect your identity. If you would like study updates, we will ask you to provide your phone number and/or email address, but you don't have to share this information if you don't want to. As soon as your participation is completed or if you withdraw from the study, any contact information that you shared with us will be erased.

For more information, email CalScope@cdph.ca.gov.

March 2022 • © 2022, California Department of Public Health

What will you do with participant information?

Information collected from this study is stored on a secure server and is password protected. All the researchers who are part of this study must receive training and certifications to make sure they know how to properly handle data and protect the privacy of all participants. Any survey responses or test results will NOT be shared with other groups that are not involved in this study.

Information from this project will be analyzed and reported as a group for overall trends. This means that there is no way someone will be able to find or see your specific survey or test results and be able to identify you. Since all survey responses and test results are collected anonymously (without names), your personal information cannot and will not be shared with any other governmental program.

Who is leading this study?

CalScope is a study run by CDPH, together with Stanford University School of Medicine and seven local county health departments (in Alameda, El Dorado, Kern, Los Angeles, Monterey, San Diego, and Shasta counties).

Do I need to speak English or be a US citizen to join the study?

NO. All study materials and surveys will be available in English, Spanish, Tagalog, and simplified Chinese. Assistance is also available for other languages. Our study does NOT ask about citizenship status.

Do I need a computer with internet access to participate?

NO. You can sign up using your smartphone or mobile phone with a data plan that allows you to access the internet. If you don’t have access to the internet, you can register and take the survey by phone at 1-833-580-1333.

Can I join the study if I have been vaccinated?

YES. You can join the study whether or not you have had COVID-19 before or have been vaccinated. It is important to have a diverse group of participants for this study, whether you have been vaccinated or not.

Can I participate again if I have joined the study before?

YES. If you already completed this study in spring or fall of 2021, and your household received another invitation, you can choose to participate again. Up to 1 adult and/or 1 child per household can join the study.

Can more than one adult participate per household?

NO. Only 1 adult (ages 18+) and/or 1 child (ages 1 to 17) can join the study from each household. If there are no children in the household, you are still only allowed to have 1 adult from the household join the study.

© 2022, California Department of Public Health

Do I have to complete both the survey and the blood sample to be part of the study?

NO. Although we would like to collect both the survey and a blood sample from all participants, we understand that it may not be possible for everyone. You can choose to only complete the survey and not complete the blood/sample test kit.

Will I receive my blood test results?

YES. If you submit a finger-prick blood sample using the at-home test kit, you will receive your results by mail in 6-8 weeks. Your results will tell you if you have SARS-CoV-2 antibodies or not.

Will the antibody test tell me if I am currently infected?

NO. The free antibody test will not be able to tell if you are currently infected with the virus that causes COVID-19 (called SARS-CoV-2).

Will I receive any payment for taking part in the study?

YES. To thank you for your time, you will receive a $20 gift card for finishing the survey and a $20 gift card for returning a completed test kit. You can choose to receive an electronic gift card by email or a physical gift card by United States Postal Service (USPS) mail. You can get up to $40 for one adult and $40 for one child in your household.

March 2022 • © 2022, California Department of Public Health

Help us learn more about COVID-19!

CalScope

Did you get an invitation in the mail from the California Department of Public Health (CDPH) asking you to join the CalScope COVID-19 antibody study?

CDPH California Department of Public Health

Stanford MEDICINE

[county logo here]

Your household could earn up to $80!

CDPH has randomly selected households in your county to understand how many Californians have antibodies (from past infection or vaccination) to the virus that causes COVID-19.

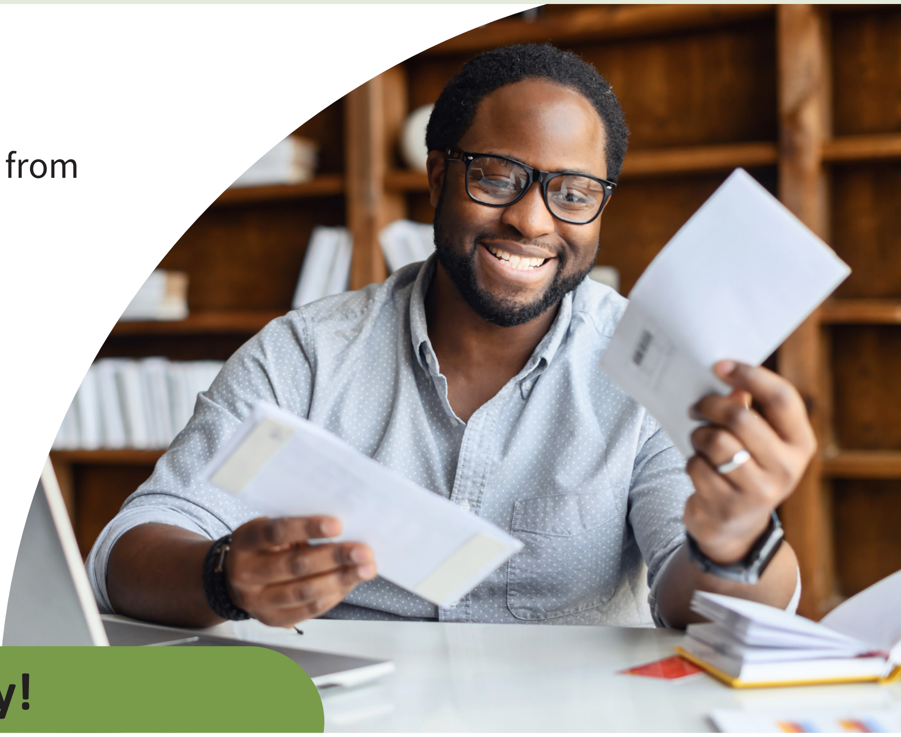

Who can join?

1 adult (18+ years) and 1 child (1-17 years) from invited households.

What do I need to do if I sign up?

1. Take an anonymous survey with questions on COVID-19.
2. Take a free and anonymous COVID-19 antibody test using a finger-prick blood test sent to your home.

Represent your community!

If you received an invitation, visit www.CalScope.org and click "I got an invitation" or call 1-833-580-1333 to sign up today!

Your household must be selected to participate in the study. Questions? Call 1-833-580-1333 or email CalScope@cdph.ca.gov.

Have you been invited to join CalScope?

CalScope is a study from the California Department of Public Health (CDPH) that will help us learn how many Californians have antibodies (from a past infection or vaccination) to the virus that causes COVID-19. Your participation can help prevent and stop the spread of COVID-19.

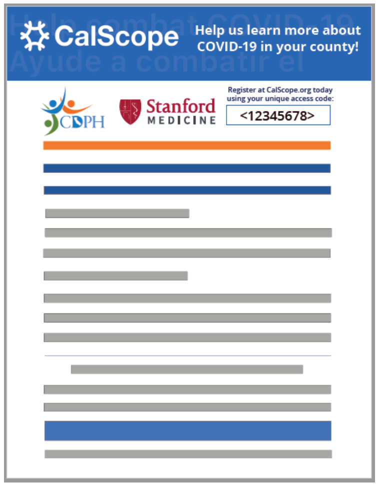

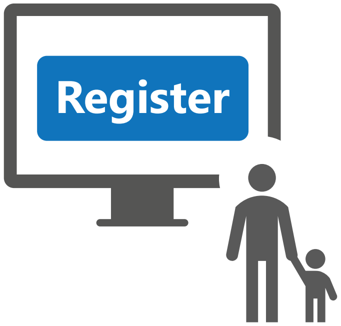

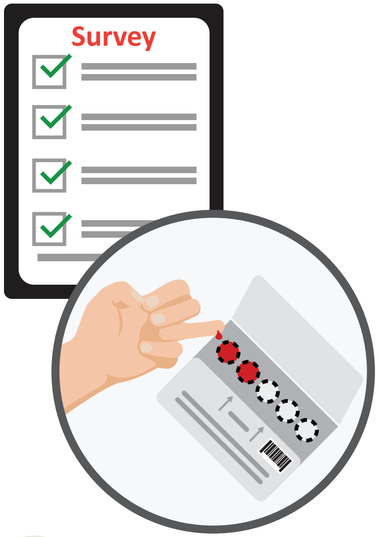

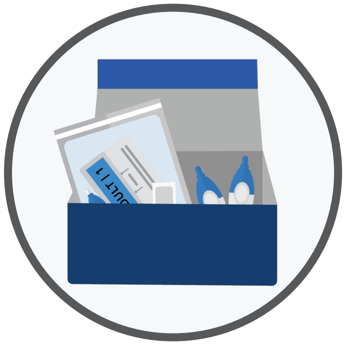

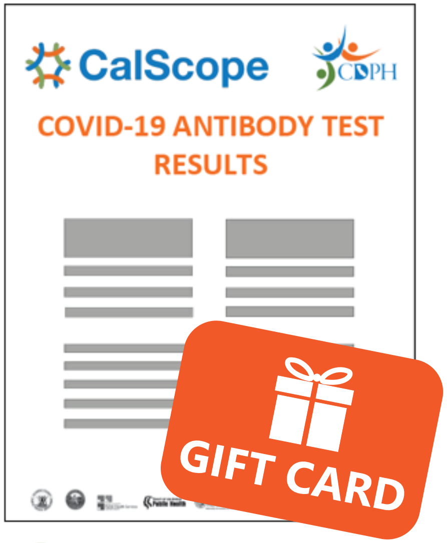

1 CDPH mails invitation letters to randomly selected households in your county.

2 Up to 1 adult and 1 child per household register online or over the phone and order at-home antibody test kit(s).

3 Adult and/or child complete online survey(s) and finger-prick blood sample(s).

4 Return completed test kit(s) with blood sample(s) by mail.

5 Receive your results by mail in 6-8 weeks. Get a $20 gift card for each completed survey and each kit. Up to $80 per household!

For more information, email **CalScope@cdph.ca.gov** or visit **www.CalScope.org**

CalScope – California's COVID-19 Antibody Study

Communications Plan for Local Partners

Goal of CalScope

CalScope is a study run by the California Department of Public Health (CDPH) that aims to learn how many people in California have antibodies to the virus that causes COVID-19. Antibodies can be acquired by either past infection or vaccination. Over the course of CalScope's three waves of data collection, CDPH is learning how population immunity to COVID-19 is changing over time in California and assessing more effective ways to stop the spread.

Learn more about the importance of CalScope in the CalScope Fact Sheet for Local Partners.

How Your Organization Can Help

As a trusted source in your community, your organization can help invited participants learn more about CalScope and encourage them to participate. Only individuals who receive an invitation can participate in the study. Your organization can help engage community partners and participants both before and during Wave 3, which begins April 1, 2022. The last day to sign-up is July 29, 2022.

Promotion Timeline

Wave 3 of CalScope will be divided into three smaller sub-waves. CDPH will send initial invitations for the first sub-wave of the study in early April. The second sub-wave will begin in early May and the last sub-wave will begin in early June. Reminder postcards to encourage participation will be sent out about 6 weeks following the start of each sub-wave. The last day to join the study is July 29, 2022. Promotion activities include connecting with local partners, promoting the study online, and sharing information. You can schedule social media outreach using the CalScope social media calendar.

How Can My Organization Share Information about the CalScope Antibodies Study?

1. **Connect with local partners** who may already be sharing information about COVID-19, or partners who can help reach underserved populations.
   - **Activity**: Share introductory outreach email and a link to our CalScope communications toolkit with local partners, or share slides about the project. Even easier, connect them directly with the CDPH CalScope team! Help us educate local partners about CalScope and encourage them to help increase participation.
   - **Stakeholders**: May include county vaccine taskforces, testing sites, community clinics, schools and universities, local libraries, promotora groups, chambers of commerce, nonprofits serving vulnerable populations, service organizations, immigrant serving organizations, community foundations, etc.

For more information, email CalScope@cdph.ca.gov.

 © 2022, California Department of Public Health

2. **Promote CalScope online and through your social media channels**
    - **Activity**: Post a logo/image graphic on your webpage to connect community members to the CalScope website, share and link information in your e-newsletters to CalScope website, and use our social media calendar from the toolkit to post information about CalScope.
    - **Stakeholders**: May include local public health departments and community-based organizations, web contributors and social media managers.
3. **Share information about CalScope in high traffic community areas**
    - **Activity**: Identify high-traffic areas/venues in the community to post print materials or ads, print and distribute CalScope flyers, connect with local radio stations or newspapers to run ads.
    - **Stakeholders**: Venues sharing COVID information like testing sites or vaccine sites. High-traffic public places like grocery stores/markets, laundromats, community clinics, churches, libraries, food pantries, city halls, popular radio stations, public transportation, etc.

Key Points

- CalScope is intended to complement existing COVID outreach and messaging, not replace current efforts to promote vaccination/testing.
- Results from CalScope can be utilized by local health departments to determine how additional COVID outreach may be needed in their counties.
- Understanding how COVID-19 antibodies may differ across groups including children, working adults, and older adults with underlying medical conditions can also help public health identify which populations could benefit from additional resources.
- Only individuals who received a CalScope invitation can participate in the study. While we want the public to be aware of CalScope and know that it’s backed by trusted partners, the opportunity to participate is only available to people who are randomly selected.
- The CDPH CalScope team is available to answer any questions, as well as provide outreach to local partners on behalf of the local health department. Share feedback on how this study can best address your community’s needs. Direct questions about this project from your local community to the CalScope study team at **CalScope@cdph.ca.gov**. Our team will work with local public health departments in partnering counties to answer questions and address any concerns. We’re here to help!

CalScope - California's COVID-19 Antibody Study

Social Media Calendar for Local Partners

CalScope's wave 3 will begin in April 2022. You can follow along with this social media calendar to post messages and graphics about CalScope during key times. Dates are approximate.

<table>
<tr><th>Week of</th><th>Image</th><th>Messages</th></tr>
<tr><td>3/21</td><td>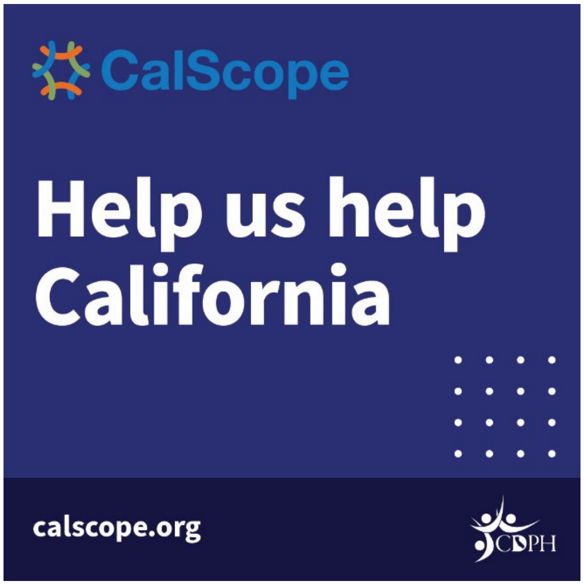
</td><td>Wave 3 of the #CalScope study is coming! What is CalScope? @CAPublicHealth and @Stanford have developed a study to learn more about 1) how many people in California have antibodies to the virus that causes COVID-19 (either from past infection or vaccination), 2) how population immunity is changing throughout our state, and 3) how this impacts our county. Through CalScope, households in our county are randomly picked to complete a survey and take a free, at-home blood test for COVID-19 antibodies — and your household could earn up to $80! If you receive an invite, you can help fight COVID-19 in CA! Learn more: www.CalScope.org

(Facebook only)</td></tr>
<tr><td>3/28</td><td>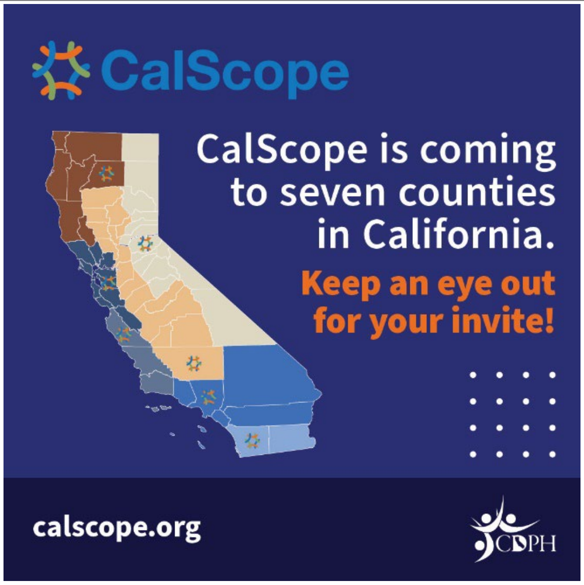
</td><td>In April, @CAPublicHealth is randomly sending #Calscope study invites to homes in our county to take an anonymous survey and at-home COVID-19 antibody test to help us learn how many in CA have COVID-19 antibodies and how this can change over time. Learn more: www.CalScope.org</td></tr>
</table>

CalScope - California's COVID-19 Antibody Study

Social Media Calendar for Local Partners

4/4	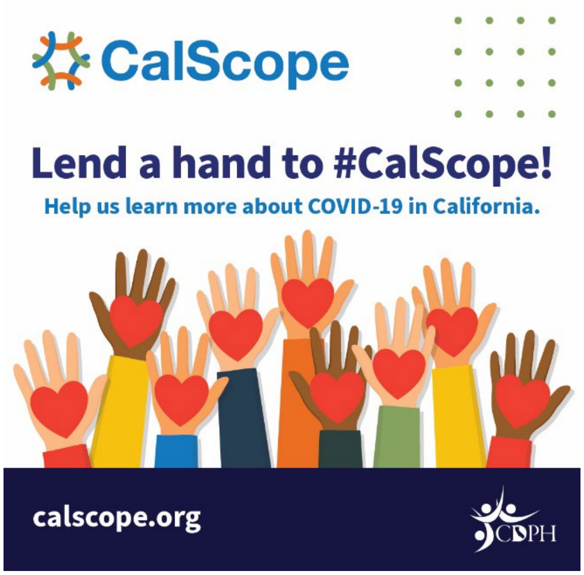	Dropping this week! Look out for your #CalScope study invite in the mail from @CAPublicHealth. Help us learn about COVID-19 antibodies in CA and our county. Households are randomly picked to join. Your participation is free and anonymous. Learn more: www.CalScope.org
4/11	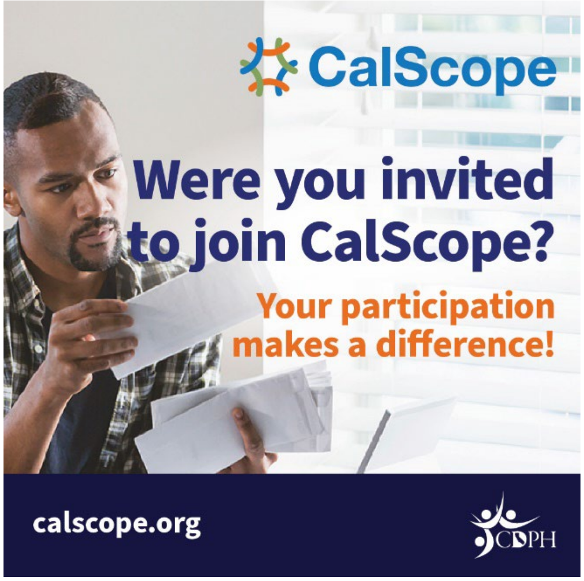	Did you get an invite from #CalScope? If so, you can help CDPH learn how many in CA have antibodies to COVID-19. Households from our county are randomly picked to join – your participation is free, anonymous, and your household could earn up to $80! Learn more: www.CalScope.org
4/18		--Skip Week--

4/25	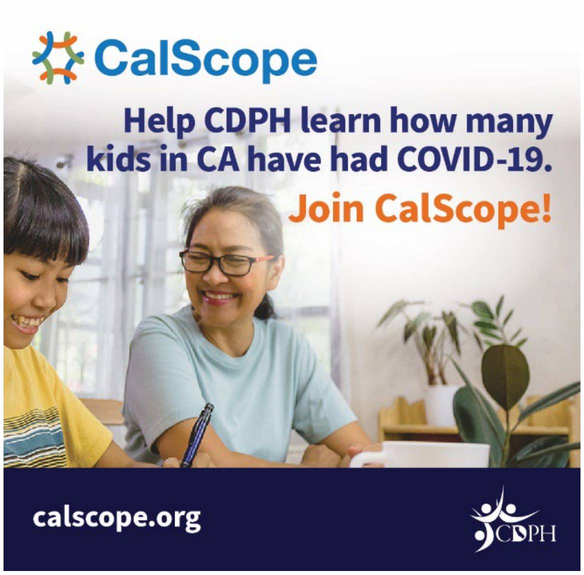	Did you know kids can also participate in the #CalScope study? Households that are randomly picked to join can sign up 1 child to take an anonymous survey and free COVID-19 antibody test. Help us learn how many kids in CA have had COVID-19. Learn more: www.CalScope.org
5/2	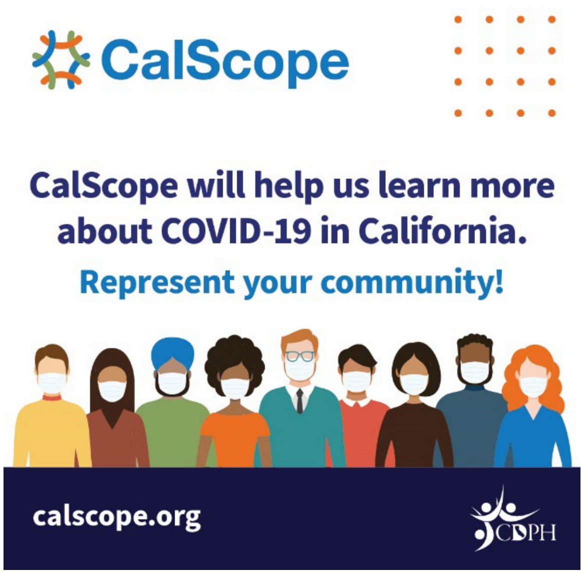	@CAPublicHealth is working with our county for the #CalScope study to learn how many kids & adults in CA have COVID-19 antibodies. Households in our county will be randomly picked to join. If you get a CalScope invite, help us in the fight against COVID-19! www.CalScope.org

CalScope - California's COVID-19 Antibody Study

Social Media Calendar for Local Partners

5/9	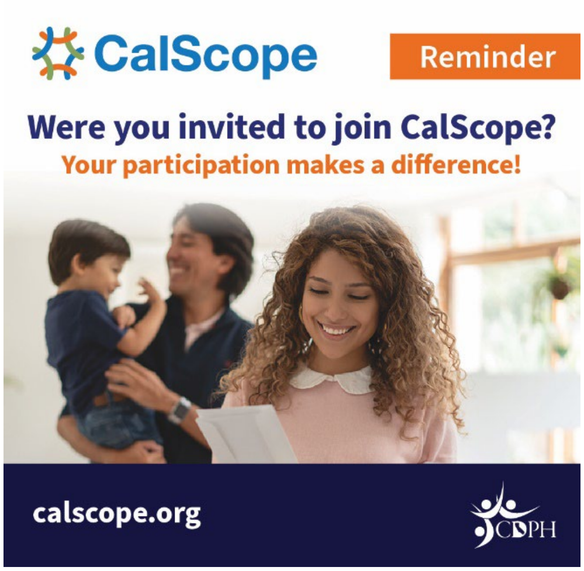	Your participation matters! Did you receive an invite in the mail from #Calscope? If so, here's your reminder to complete your survey and free at-home COVID-19 antibody test. We can't do this without you! Learn more: www.CalScope.org
5/16		--Skip Week--
5/23	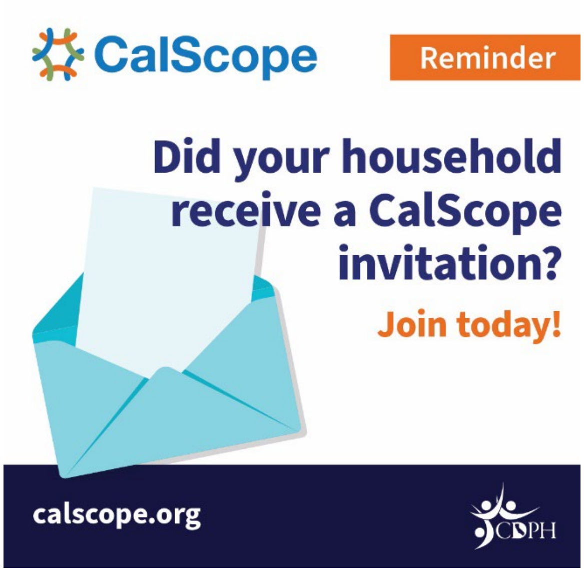	Reminder! Did you receive an invite from #Calscope? If so, here's your reminder to help us understand how COVID-19 immunity is changing throughout our state and how this impacts our county. Plus, your household could earn up to $80! Learn more: www.CalScope.org

5/30	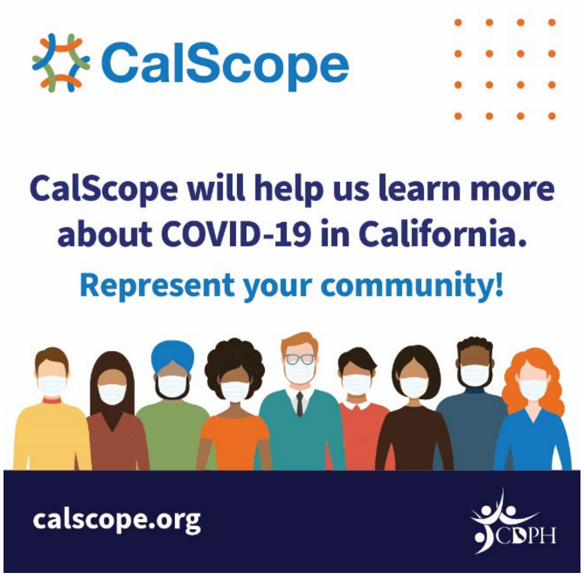	Did you receive an invite from #Calscope? Your participation matters! You can help CDPH understand how immunity to COVID-19 has changed following the Omicron variant. Learn more: www.CalScope.org.
6/6	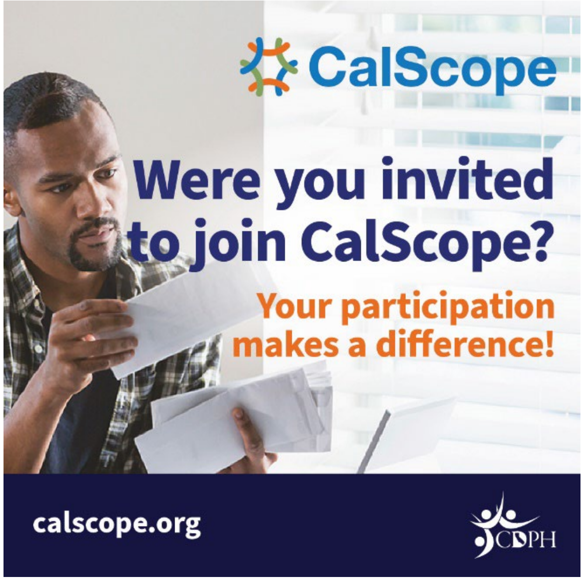	Did you get an invite from #CalScope? You can help CDPH learn how many people in CA have antibodies to COVID-19. Households from our county are randomly picked to join – your participation is free, anonymous, and your household could earn up to $80! Learn more: www.CalScope.org

6/13	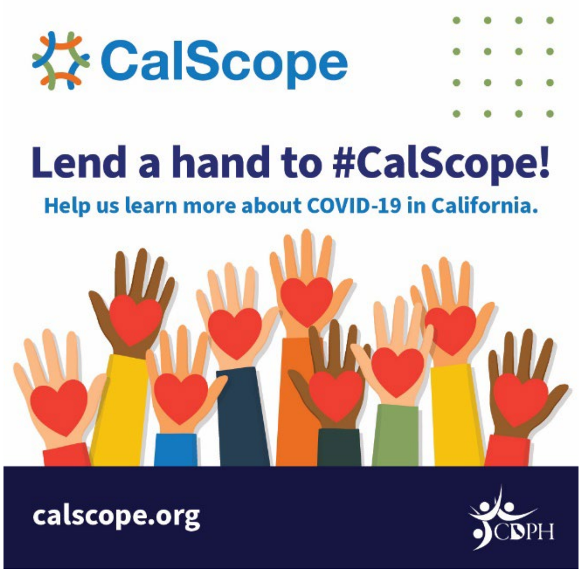	Reminder! Invited to #Calscope? If so, here's your reminder to help us understand how COVID-19 immunity is changing throughout our state and how this impacts our county. Plus, your household could earn up to $80! Learn more: www.CalScope.org
6/20	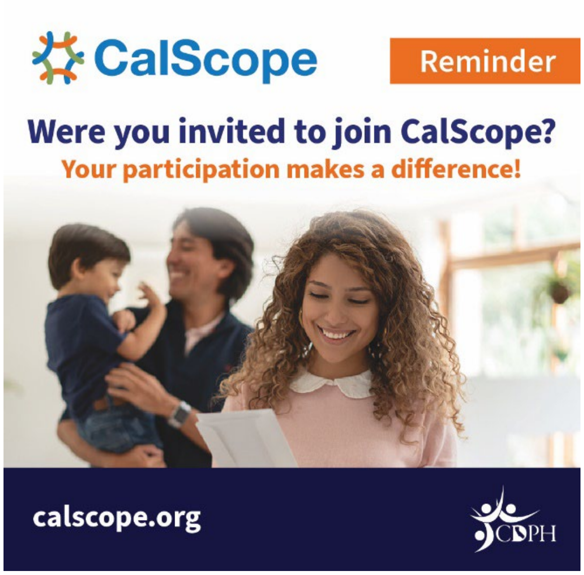	Help us understand how Omicron has affected immunity to COVID-19 in California! If you received an invite from #CalScope, your participation matters. Learn more: www.CalScope.org.

<table>
<tr><td>6/27</td><td>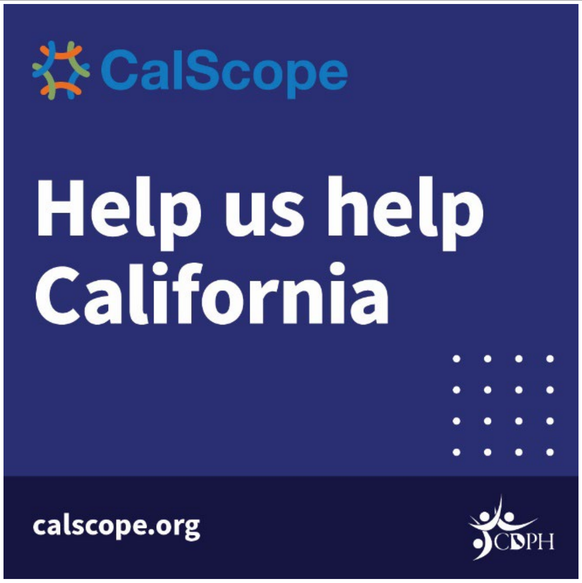
</td><td>Your participation matters! Did you get an invite from #CalScope? Help us learn about COVID-19 antibodies in CA and our county. Households from our county are randomly picked to join and your household could earn up to $80! Learn more: www.CalScope.org</td></tr>
<tr><td>7/4</td><td></td><td>--Skip Week--</td></tr>
<tr><td>7/11</td><td>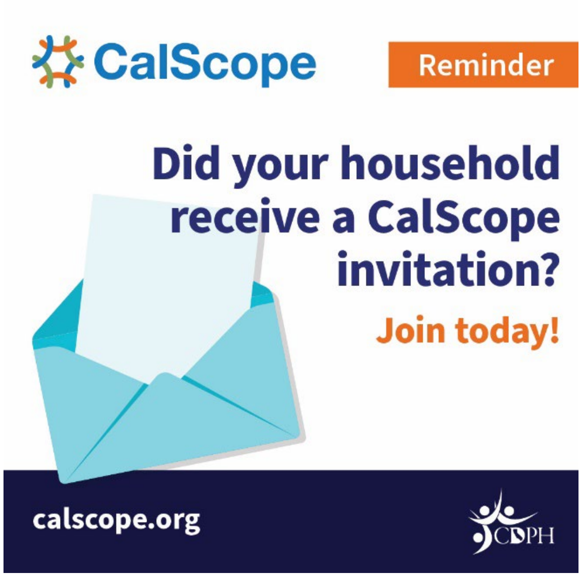
</td><td>Here’s your reminder to participate in #CalScope. Help us understand how immunity to the COVID-19 virus is changing throughout California and how this impacts our county. We need your help! Learn more: www.CalScope.org</td></tr>
</table>

7/18	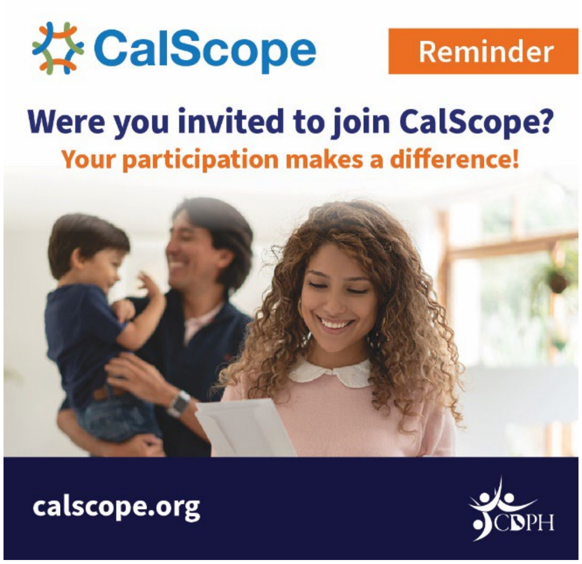	If you received an invite from #Calscope, the deadline to sign up is July 29. Help us understand how immunity to the COVID-19 virus is changing throughout California and how this impacts our county. Plus, your household could earn up to $80! Learn more: www.CalScope.org
7/25	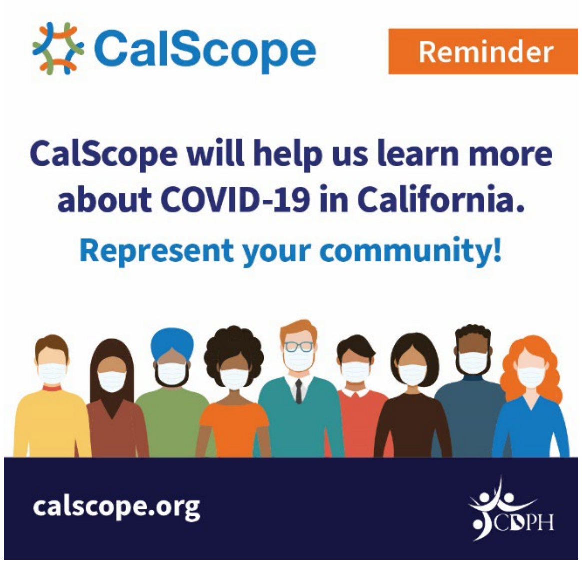	Last chance to represent our county in the #CalScope study! Enrollment closes this week! Help us understand how immunity to the COVID-19 virus is changing throughout our state and how this impacts our county. Plus, your household could earn up to $80! Learn more: www.CalScope.org

Have you been invited to join CalScope?

Learn how you can help fight COVID-19 in California!

calscope.org

CalScope

CDPH

Have you been invited to join CalScope?

Learn how you can help fight COVID-19 in California!

calscope.org

CalScope

CDPH
