# Supplementary material for: CalScope: methodology and lessons learned for conducting a remote statewide SARS-CoV-2 seroprevalence study in California using an at-home dried blood spot collection kit and online survey
Source: BMC Med Res Methodol. 2024 May 27;24:120. doi: 10.1186/s12874-024-02245-y (PMC11131314; doi:10.1186/s12874-024-02245-y)
Supplement: Supplementary file 1 — Supplementary Material 1. [file 12874_2024_2245_MOESM1_ESM.zip › I. REDCap External Modules.pdf]

F. REDCap External Modules

Customizable software packages called external modules (EM) maintained inter-REDCap communications to regularly update main study records with access code, gift card, and IVR registration information based on conditional logic. The 'AutoContinue Logic'[6] EM determined which language version of each form appeared in a survey queue based on the response submitted in the first language selection form. For survey queues not completed in English, the 'Copy Data on Save'[7] EM copied each variable to the corresponding English variable, consolidating all responses into a single English form to use for analysis and streamline the logic used to determine survey completion for a record. This module was also used to claim the next available gift card record and copy over the information to the appropriate record for disbursement. Within each survey, the 'Shazam'[8] EM allowed incorporation of CSS stylizations for reminders and warning messages.

The 'Stand Alone REDCap + Twilio IVR'[9] EM managed a Twilio-enabled IVR script tree which used descriptive fields to hold audio recordings of each variable, followed by a multiple choice (radio) field to save keypad responses provided by the caller. Each variable was recorded in 4 different versions (English, Spanish, Filipino/Tagalog, Mandarin) and the script began with a single language selection recording (spoken in all four languages) which determined the next recording to play using a branching logic as it flowed top to bottom.
