# Supplementary material for: CalScope: methodology and lessons learned for conducting a remote statewide SARS-CoV-2 seroprevalence study in California using an at-home dried blood spot collection kit and online survey
Source: BMC Med Res Methodol. 2024 May 27;24:120. doi: 10.1186/s12874-024-02245-y (PMC11131314; doi:10.1186/s12874-024-02245-y)
Supplement: Supplementary file 1 — Supplementary Material 1. [file 12874_2024_2245_MOESM1_ESM.zip › J. Target recruitment rate.pdf]

E. Target recruitment rate

Based on conversations with academic partners in California who had conducted similar seroprevalence studies using a mailed invitation letter, we estimated that our target enrollment rate would be 5%. Furthermore, we estimated that a minimum of 500 samples from each region was needed per wave to ensure stable region-specific estimates of anti-SARS-CoV-2 antibody seroprevalence at each time point. Our sampling partner (Marketing Systems Group, Horsham, PA), also provided additional information that approximately 30% of households within each sample were likely to have at least one child in residence, submitting potentially two samples within the same household (1 adult and 1 child). As such, approximately 200,000 households per timepoint were invited, meeting the minimum requirement as well as staying within the budget which allowed for a maximum of 15,000 tests by the laboratory.
