# Supplementary material for: CalScope: methodology and lessons learned for conducting a remote statewide SARS-CoV-2 seroprevalence study in California using an at-home dried blood spot collection kit and online survey
Source: BMC Med Res Methodol. 2024 May 27;24:120. doi: 10.1186/s12874-024-02245-y (PMC11131314; doi:10.1186/s12874-024-02245-y)
Supplement: Supplementary file 1 — Supplementary Material 1. [file 12874_2024_2245_MOESM1_ESM.zip › H. Phone script_ESM.pdf]

Telephone Call Script: Return Call

Protocol Title: CalScope

Purpose:

To be used when returning support requests logged through the REDCap support module by phone (IVR), email, or the CalScope website's "Contact Us" form. The following script provides a general script for how to introduce yourself and answer questions or problems, but conversations do not have to or will go exactly as outlined below. Please refer to the CalScope FAQs and the Call Center Protocol for more specific information about the CalScope study.

Table of Contents

Returning a Support Request by Phone Call

Voicemail

Help with Study Sign-Up

Voicemail

Complete Surveys Over the Phone

Registration Phase

Kit Activation Phase

Voicemail

Scheduling Follow-Up Calls

Left-Field Questions and Possible Responses

I did not get a letter/kit box, how can I join the study?

Is there a direct number that I can call to speak with someone right away?

Common Questions

Privacy

Confidentiality

Will I get my blood test results back?

What does a positive result mean for me?

How long does the survey take?

How long is the study for?

Can the whole family participate?

What if I only want to answer the survey and not do the blood collection?

Do I need to answer all the questions on the survey?

What if I change my mind later and don't want to participate any longer?

Can my friend or neighbor participate in the study as well?

Why is this study important? How will I benefit from this study?

Should I still participate if: I had COVID-19 before, get regularly tested for COVID-19, have been vaccinated, never leave my house, etc.?

Returning a Support Request by Phone Call

Hello, my name is <<first name>> and I'm calling on behalf of the CalScope study from the California Department of Public Health.

How are you doing today?

<<Good or what is this about? Proceed>>

> *<<re: questions>>* I'm calling because we received your request for more information about <<question>> by <<method: phone, email, website>>. Is this a good time for me to try and go through this question with you right now?
>
> *<<OR>>*
>
> *<<re: problems>>* I'm calling because we received your request to get help with <<problem>> by <<method>>. Is this a good time for me to try and help you with this problem right now?

<<Pause>>

A. No- I am busy right now

That's alright, I'm sorry for calling you at an inconvenient time! Is there a better time today or tomorrow that I could call you back? <<collect date/time>> Okay, I will try to call you again <<date/time>>.

Thank you so much for your time and I will look forward to speaking with you later again. Goodbye!

B. Yes- I have time right now

Great! <<Explain question/issue/etc.>>

> *<<If additional follow-up is required, collect or communicate the following information:>>*
>
> *1. Method of communication: Email or Phone*
>
> *2. Date/Time of expected follow-up*
>
> *3. Best time for call during the day*
>
> > *<<If the staff member needs to find out more information before follow-up>>*
> >
> > I think I should be able to figure this out and get back to you by <<date>>. How does that sound? <<Record date>>
> >
> > Would you like for me to call you back at this same phone number? I can also send an email if that would be better for you. <<Record method of communication requested>>
> >
> > <<If by phone>> What time of day would be best for me to get a hold of you by phone again? <<Record time>>
> >
> > <<If the participant needs to find out more information before follow-up>>
> >
> > Okay, when should I try to follow-up with you again to see if you were able to work everything out? <<Record date>>
> >
> > Would you like for me to call you back at this same phone number? I can also send an email if that would be better for you. <<Record method of communication requested>>

<<If by phone>> What time of day would be best for me to get a hold of you by phone again? <<Record time>>

VOICEMAIL (ONLY FOR PARTICIPANTS WITH [MISSED_CALL]^=NO(2)):

Hello, I am calling from the CalScope study here at the California Department of Public Health. We received your <<question about.../request for help with...>>. I will try calling you again tomorrow at <<different time>>, but you can also send us an email to calscope@cdph.ca.gov for a quicker response if needed. Thank you!

Help with Study Sign-up

Hello, my name is <<first name>> and I'm calling on behalf of the CalScope study from the California Department of Public Health.

How are you doing today?

<<Good or what is this about? Proceed>>

<<re: help with study sign-up>> I'm calling because we received your request for help with signing up for the CalScope study <<online/by phone>>. Is this a good time for me to help you with registration right now?

1. No- I am busy right now

That's alright, I'm sorry for calling you at an inconvenient time! Is there a better time today or tomorrow that I could call you back? Registration should only take about 5 minutes to complete. <<collect date/time>> Okay, I will try to call you again <<date/time>>.

Thank you so much for your time and I will look forward to speaking with you later again. Goodbye!

2. Yes- I have time right now

Great! Do you have the invitation letter or postcard with instructions on how to register for the study that was mailed to your home? *<<Needed to indirectly confirm whether resident lives within a target address>>*

a. Yes- I have the letter/postcard with me right now

Great! Would you please read off the 8-digit access code printed on your <<letter/postcard>>, along with your zip code? <<Assist with registration survey online and confirm information- # of kits>>

<<if email or phone number provided>>
Alright, you should receive a confirmation of your registration by <<email/text>> at the <<email address/phone number>> you provided.

<<closing statement>>
You will be receiving your test kit(s) by mail within the next week or so. When you get your test kits, you will need to complete the surveys online with the activation code that's printed on your test box. If you have any issues with finishing those online, give us a call or send us an email to ask for help and we can help you complete those over the

phone. You can also ask for help if you have trouble doing the finger-prick blood collections.

We're available to help you every step of the way!

Did you have any questions that I could answer before I let you go?

<<Closing>> Well, thank you so much for your time and for your interest in the CalScope study. Please contact us again by email or phone if you have any other questions or issues!

b. No- I don't have the letter/postcard with me right now

That's alright. Would you be able to find it right now or should I give you a call again at a different time? We need the 8-digit access code that's printed on the <<letter/postcard>> to help you with your registration.

<<If yes- go to step 2a.>>
<<If no, call different date/time>>

I'm sorry for the inconvenience! What is a better date and time that I should try calling you again? Registration should only take about 5 minutes to complete. <<collect date/time>> Okay, I will try to call you again <<date/time>> at this same phone number.

Thank you so much for your time and I will look forward to speaking with you later again. Goodbye!

c. No- I lost the letter and/or postcard

That's alright. I can try and look up your access code based on your address. <<Collect address information to look up on database>>.

<<If address and access code found>>

Great, I see the access code that's been assigned to your mailing address. I can help you with your registration over the phone right now! <<If yes, proceed to 1a>>

<<OR>>

<<If address and access code not found>>

I'm sorry but I am not seeing your address come up as one of the randomly selected households in our system. Did you receive both the letter and postcard by mail at your address?

<<If letter but not postcard>> I will check with my team to try and figure out why we can't find your address in our system, but in the meantime you should also receive a postcard with your access code in a couple of days. If you get that before you hear back from us, please use the access code on the postcard to register. Is this the best number to get in contact with you? <<Collect any additional call back information- best times to call, etc.>>

<<Closing>> Well thank you so much for your time and interest in the CalScope study and we will make sure to get back to you as soon as possible! Goodbye.

<<If no letter at all>> If you never received a letter from the study with an access code, your household was not randomly chosen to join the study. Your household may be selected to join later as we plan to reach out to more households in the future, but right now your household cannot join the study at this time. Do you have any questions?

<<Closing>> Well thank you so much for your time and interest in the CalScope study! Please visit covid19.ca.gov if you have any general questions about COVID-19 in California. Goodbye.

<<If both letter and postcard is lost>> Let me check back with my team to figure out why your address is not showing up on our system. Can we circle back with you later? Is this the best number to get in contact with you? <<Collect any additional call back information- best times to call, etc.>>

<<Closing>> Well thank you so much for your time and interest in the CalScope study and we will make sure to get back to you as soon as possible! Goodbye.

Additional Responses:

a. **Closing statement- If participants want to end the conversation by saying that they are no longer interested in participating:**

   I'm sorry to hear that. Well, thank you for your time and please feel free to contact us if you ever change your mind down the line.

Voicemail (only for participants with [missed_call]=Yes(1)):

Hello, I am calling from the CalScope study here at the California Department of Public Health. We received your request for help with signing up for the study. I will try calling you again tomorrow at <<different time>>, but you can also send us an email to calscope@cdph.ca.gov for a quicker response if needed. Thank you!

Complete surveys over the phone:

Hello, my name is <<first name>> and I'm calling on behalf of the CalScope study from the California Department of Public Health.

How are you doing today?

<<Good or what is this about? Proceed with either scenario>>

1. Adult/Child Surveys without Blood Test (Registration Phase)

I'm calling to help you complete the online survey that you chose to do for the <<Adult/Child>> joining the study without collecting the blood sample. Is this a good time for me to help you complete that survey right now?

<<OR>>

2. Adult/Child Surveys with Blood Test (Kit Activation Phase)

I'm calling to help you complete the online survey for your test kits over the phone. Is this a good time for me to help you complete for the survey for the <<ADULT and/or CHILD>> right now?

<<Possible Responses>>

1. No- I am busy right now

I'm sorry for calling you at an inconvenient time! Is there a better time today or tomorrow that I could call you back? Registration should only take about 5 minutes to complete. <<collect date/time>> Okay, I will try to call you again <<date/time>> at this same phone number.

Thank you so much for your time and I will look forward to speaking with you later again. Goodbye!

2. Yes- I have time right now

Great! Do you have your test box with you right now?

a. Yes- I have the test box with me right now

Great! Would you please read off the 6-character activation code printed on the label inside your test box? It should be right underneath the lid and starts with the letter "C" as in California. <<Type in response to calscope.org/#gotkit>>. Can you also provide your zip code for me? <<Verify activation and confirm number of surveys (Adult and/or Child) to be completed. Assist with survey completion. Walk through blood collection process if needed. Make sure participant fills out DBS label, dries card for 4 hours, and knows how to return the completed test kit by USPS mail using the enclosed envelope.>>

i. <<Chose to receive gift card by email or text>>

Alright, you should receive your $20 gift card link by <<email/text>> in the next day or two. If you have any issues accessing your gift card, you can either contact us or Tango directly, that's the vendor that the gift card is coming through.

<<OR>>

ii. <<Chose to receive physical gift card by mail>>

Alright, you should receive your $20 <<brand>> gift card by mail in the next 1-2 weeks.

<<AND IF>>

<<For participants completing blood collection>>

We will also send the $20 gift card for the test kit by <<email/text/mail>> when the lab receives it in the mail, and it takes about the same time to get sent out from that date. The results will also be sent to your home address about 4 weeks after our lab receives the test kits.

<<closing statement>>

Did you have any questions that I could answer before I let you go?

b. No- I don’t have the test box with me right now

That’s alright. Would you be able to find it right now or should I give you a call again at a different time? We need the 6-character activation code that’s printed on the test box to help you with your online surveys. The code should start with the letter “C” as in California.

<<If yes- go to step 2a.>>
<<If no, call different date/time>>

I’m sorry for the inconvenience! What is a better date and time that I should try calling you again? Registration should only take about 5 minutes to complete. <<collect date/time>> Okay, I will try to call you again <<date/time>> at this same phone number.

Thank you so much for your time and I will look forward to speaking with you later again. Goodbye!

VOICEMAIL

Hello, I am calling from the CalScope study here at the California Department of Public Health. I called to help you complete the online <<Adult/Child>> survey over the phone as requested for the study! We will try calling you again tomorrow at <<different time>>. Thank you!

Scheduling a Follow-Up Call

If you need to re-schedule with a participant outside of your scheduled shift and the participant is currently on the phone-

- **SAME-DAY**:
    1. **CalScope/CalCONNECT Teams** communicate it with the team so someone else can make the follow-up call.
    2. **If no one confirms through Teams before end of your shift** email xxx@cdph.ca.gov or call/ text xxx-xxx-xxxx.
- **NEXT or DIFFERENT DAY:**
    1. **Document on REDCap Follow-Up form** with RECORD ID, follow-up date/time, and notes for the record.
    2. **If request time is before 9AM next day** communicate it with the team through Teams chat to make sure someone else can make the follow-up call.

Left-field Questions and Possible Responses:

1. I did not get a letter/kit box- how can I join the study?

Unfortunately, only randomly chosen households that receive a mailed letter or postcard from the study may join the study at this time. However, we hope to reach out to additional households in the future and you may receive an invitation at that time.

2. Is there a direct number that I can call to speak with someone right away?

No, unfortunately we don't have a phone line that is staffed with a person that can answer calls so we can only return voicemails and communicate by phone or over email. We apologize for the inconvenience.

Common questions:

Privacy

All information will be kept within a secure server and accessed using only password protected computers that only the research team directly involved in the study will use. All our researchers also must receive training and certifications to make sure that they know how to properly handle data to protect the privacy of all our participants. We will also not be collecting any personally identifying information from participants, and any contact information such as phone numbers and addresses will be kept separate from survey and test data.

Confidentiality

We will not be collecting any participant information such as names and date of births. All surveys and test samples will be tracked using a code. Your test results will only be returned to you by mail using the code of the test kit you used to collect the sample. All results will not be attached with your name or any other identifying information, other than age and/or address.

Will I get my blood test results back?

Yes! You will be able to receive the blood test results for your records within 4 weeks after our lab receives your blood spot cards.

What does a positive result mean for me?

A positive antibody test or a result that says "antibodies are present" means that the participant may have been infected with the coronavirus (SARS-CoV-2) in the past and has developed antibodies. An antibody test does not show if they have a current infection since it can take 1-3 weeks or longer to develop antibodies. The antibody test may also be positive if the participant has received a vaccination to protect from the coronavirus (SARS-CoV-2).

However, it is also possible that the test may be wrong, and the participant has never been infected with the virus in the past. This kind of error happens infrequently (about 5-15% of the time). Because this is the first time these tests have been used for home-based testing, we can't be 100% certain of the exact accuracy of these tests yet.

Furthermore, the antibody test used in this study is meant to be used for public health purposes only. Scientists do not know if having antibodies to the virus protects you from getting infected again. The test being used has been designed and validated for research but has not been approved by the FDA. The participant should not change their behavior based on their result and should continue to follow public health recommendations.

How long does the survey take?

The survey should take only about 10-15 minutes to complete for each person.

How long is the study for?

The study only requires the one-time collection of the survey and the blood spot for testing.

Can the whole family participate?

Only 1 adult and 1 child from each household can participate. If you have multiple adults and children in the family who want to participate, the person with the next upcoming birthday should be chosen to join the study.

What if I only want to answer the survey and not do the blood collection?

That's not a problem! Although we would like to collect both the survey and a blood sample from all participants, we understand that it may not be possible for everyone, especially young children who may be afraid of needles. If so, you can choose to just answer the survey. However, if your household is not ordering any test kits and there are children in the household, you may only be allowed to complete the survey for the adult and not for both the adult and child.

Do I need to answer all the questions on the survey?

Your participation is voluntary so you may choose to skip a question if you do not feel comfortable answering a question.

What if I change my mind later and don't want to participate any longer?

Your participation is voluntary. You may change your mind about participation at any time. Please contact the study team if you no longer wish to participate in the study after registration so that you will stop receiving reminder texts/calls/emails from study staff.

Can my friend or neighbor participate in the study as well?

Each household is selected randomly to participate in the study; therefore, each registration code is specific to the address. This means that invitations and registration codes cannot be shared or given to a different household for participation.

Why is this study important? How will I benefit from this study?

The goal of this study is to learn more about how many people in California have antibodies to the virus that causes COVID-19, either because of a past infection or vaccination. This information will help us learn more about how the virus that causes COVID-19 has spread in California. Information collected from this study will also help the California Department of Public Health and local public health departments as we work to prevent and stop the spread of this virus.

As for benefits, you would receive free antibody testing through this study, which we hope you will use in addition to information on our study website and materials to learn more about COVID-19 and keep up to date with the latest research and findings.

Should I still participate if: I had COVID-19 before, get regularly tested for COVID-19, have been vaccinated, never leave my house, etc.?

Yes, as long as your household has been selected and you are willing and able to participate, please do so. Your household has been randomly selected to participate, and in order to understand how many people in California have antibodies to COVID-19, we ask that you participate regardless of your COVID-19 testing or vaccination history, or whether you think you have had COVID-19 in the past or not.
